# Agrivoltaics can reduce political polarization and local opposition to solar energy on land

**DOI:** 10.1038/s41467-026-77141-8

**Published:** 2026-08-27

**Authors:** Lukas Fesenfeld, Leon Sistek, Simon Montfort, Dionis Anderegg, Jürg Rohrer, Tobias Schmidt

**Affiliations:** 1 Einstein School of Public Policy, ETH Zürich, Zürich, Switzerland; 2 ETH Zürich, Political Economy of Technology Group, Zürich, Switzerland; 3 ETH Zürich, Energy and Technology Policy Group, Zürich, Switzerland; 4 Human-Environment Relations in Urban Systems, EPFL, Lausanne, Switzerland; 5 Institute of Natural Resource Sciences, ZHAW Zürich, Zürich, Switzerland

**Keywords:** Energy and society, Social sciences, Agriculture

## Abstract

As solar energy deployment on land intensifies, political polarization and local opposition increasingly challenge its social acceptance. We propose that agrivoltaics – a dual land-use technology that combines solar power generation with agricultural production – can help bridge ideological divides and reduce local opposition by minimizing land-use conflicts and offering additional benefits to rural communities. To test this hypothesis, we conducted a pre-registered, field-embedded experiment with a representative sample (2145 Swiss residents) just prior to a national renewable energy referendum, combining a personalized geolocated information treatment on local agrivoltaics potential with a conjoint experiment varying key technology and project design attributes. Here we show that agrivoltaics garners broad public support across ideological groups and reduces local opposition, especially when projects are well integrated into agricultural landscapes, do not significantly reduce agricultural yields, and are locally owned. These findings underscore the importance of technology and project design in addressing local and right-leaning opposition to land-based solar energy.

## Abstract (web summary)

Agrivoltaics — combining solar power with farming — wins broad public support across political divides and reduces local opposition to solar projects, especially when well-integrated into farmland, preserving yields, and locally owned.

## Introduction

Many renewable energy technologies have seen remarkable cost reductions and diffusion, but the expansion of these technologies has become a highly contested and polarized political issue^1–11^. This is particularly the case for large renewable energy projects on land, such as open-space solar photovoltaic (PV) projects, and it puts the expansion of such land-based solar energy initiatives at risk^1,2^. Right-wing populist parties across many countries have increasingly politicized solar energy, with legal initiatives to limit solar projects, such as in Italy^12^ or the United States^13^. However, even right-wing populist parties differ in how they position themselves toward the technology: some, like Germany’s Alternative für Deutschland (AfD), oppose it outright, while others, including Italy’s Lega and Fratelli d’Italia, express conditional support^1^. These divergent stances challenge the notion of a uniform ideological opposition to solar energy and underscore the importance of political strategy, technology- and project-specific factors in shaping public and party positions on renewable energy. At the same time, local opposition to renewable energy projects near individuals’ homes challenges their expansion^14–17^, and research indicates that the specific characteristics of these projects can be pivotal for generating support among local policymakers and residents^1,2,7,9–11,14,15,18–20^.

Compared to other renewable energy technologies, such as wind power plants, solar energy has only more recently become salient in public discourse and for right-wing parties^1,21–23^. Arguably, however, not all types of solar energy technologies are politically contested to the same extent. As a result, voter and party support for solar energy is likely to depend on information about local building potential and on specific project and technology design. In fact, support for solar energy in many countries is not homogeneous across different types of solar energy technologies^1,9,19^. For instance, right-leaning voters and parties across various countries tend to welcome rooftop solar projects^9^ but oppose large-scale solar projects on land, which are perceived to threaten rural esthetics, agricultural production, or local control^1,19^.

However, while rooftop solar PV receives more support—even among right-leaning and local constituencies—its costs and implementation hurdles (e.g., grid connection, small project sizes) are higher than for solar energy projects on land^24,25^. Therefore, globally, rooftop solar PV is likely to cover only a share of the total demand for renewable energy needed to meet climate change mitigation and renewable energy targets, implying the need to expand land-based solar projects to meet these goals^25^. Yet, solar energy projects on land require space and can create important cultural, environmental, and economic land-use trade-offs, for example, in the context of food and energy security^24,26,27^. Especially when agricultural land is limited, such as in countries like Switzerland, these land-use trade-offs become particularly salient. Moreover, solar energy projects on land can create other land-use trade-offs, such as spatial friction in biodiversity hotspots, human settlements, or recreational landscapes^24,26,27^.

This constitutes an important political dilemma. On the one hand, to achieve climate change mitigation and renewable energy targets and become more energy independent, policymakers cannot rely only on rooftop solar PV and need to expand solar energy projects on land. On the other hand, these land-based solar energy projects face important political risks – especially in regions where renewable energy is polarized and where land competition is high and trade-offs are particularly salient.

**Fig. 1:**
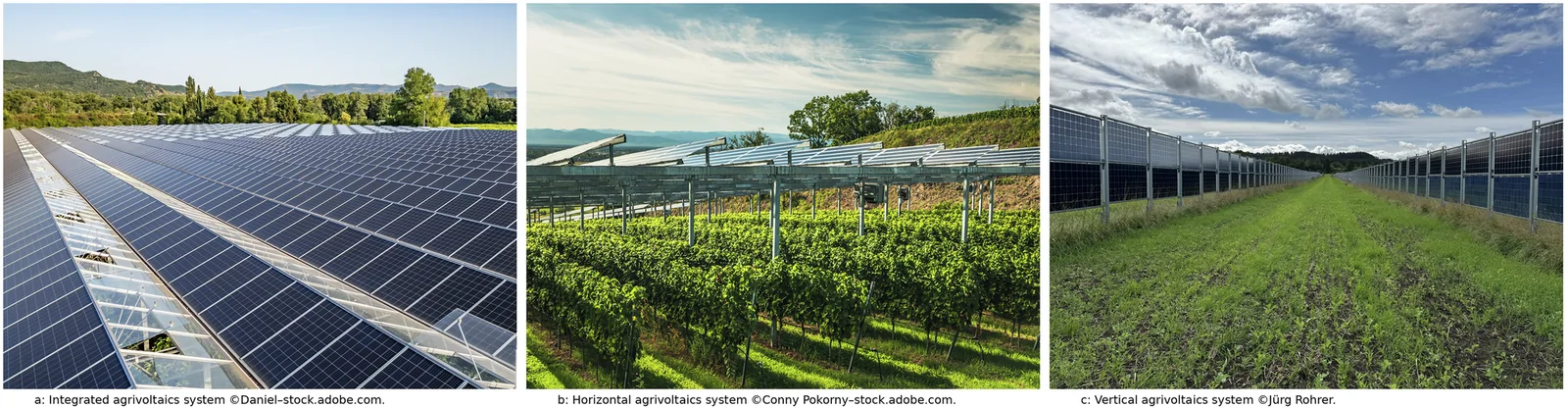
Types of agrivoltaics systems. Figure 1a shows an agrivoltaics system that is integrated into existing agricultural infrastructures (e.g., greenhouses or polytunnels) [©Daniel–stock.adobe.com]. Figure 1b depicts a horizontally elevated agrivoltaics system on pasture or arable land [©Conny Pokorny–stock.adobe.com], and Fig. 1c illustrates a vertically positioned agrivoltaics system on pasture or arable land [© Jürg Rohrer].

When considering such land-use trade-offs and the increasing polarization of land-based solar energy projects, a noteworthy recent technological advance is agrivoltaics. Agrivoltaics refers to dual land-use solar technologies that allow land to be used for both agricultural production and photovoltaic electricity generation. These systems are often integrated into existing agricultural infrastructure (e.g., greenhouses or polytunnels; see Fig. 1a), horizontally elevated on pasture or arable land (Fig. 1b), or vertically positioned to allow uninterrupted farming activity between the PV panels (Fig. 1c)^28,29^. This multifunctional design sets agrivoltaics apart from conventional ground-mounted installations by minimizing land-use competition and delivering additional co-benefits. Studies show that agrivoltaics can improve land-use efficiency^28,30^, help avoid land-use disputes^31^, and generate multiple local benefits, including more diverse income streams for farmers, improved biodiversity, and greater drought resilience^29,32,33^.

Here, we focus on the sociopolitical implications of agrivoltaics, which remain under-researched in existing studies on political behavior and public support for energy transitions^2,3,9,11,14–18,34–39^. Initial evidence suggests that agrivoltaics can increase stakeholder and public support compared to open-space solar PV, especially when aligned with local benefits such as food security and reduced impacts on landscape esthetics^34–43^. In addition, research on public support for other renewable energy technologies highlights the importance of local ownership, increasing local energy production, and higher incomes for local communities^2,18^. Such local co-benefits of renewable energy projects—for instance, ownership by local municipalities or local businesses that allows economic revenues to remain in the region rather than being channeled to outside investors and energy companies—are key for local support^2,18^. Local support is particularly important given the re-emergence of geographically defined—often rural—“policy losers,” who might be more likely to bear, or perceive to bear, the concentrated costs of renewable energies compared to urban residents^6,7,44,45^. Nevertheless, rigorous quantitative and experimental research on political polarization and local opposition to different types of solar energy technologies on land—particularly agrivoltaics—remains scarce^36^. Especially, we lack knowledge on how realistic information about the local building potential of agrivoltaics projects and their design shapes partisan support and local opposition to solar energy projects on land^1,14–19,21,46–49^.

Our central argument is that agrivoltaics’ technology- and project-specific co-benefits can help promote support for solar PV projects on land across ideological groups and reduce local opposition, particularly in politically contested contexts. Specifically, we hypothesize that agrivoltaics’ potential to increase local energy production while maintaining agricultural production (i.e., reducing the potential energy versus food production trade-off), opportunities for local ownership and increased farmer incomes (i.e., more visible local co-benefits), and the integration of solar panels into established farm structures such as greenhouses (i.e., less visible local costs) can increase public support across ideological groups and reduce local opposition – even when residents are presented with information about the realistic local building potential of agrivoltaics projects close to their homes. To test this argument, we conducted a field-embedded, pre-registered, geolocated information and conjoint experiment to analyze how personalized information about realistic local agrivoltaics building potential and the specific technology and project design of agrivoltaics affect public support across ideological groups and urban–rural populations. We fielded the experiment with a representative sample of 2,145 Swiss adult residents in the French- and German-speaking cantons just before a public renewable energy referendum in June 2024 (see further details in the Methods section below the Discussion).

Switzerland offers a particularly relevant and informative research case due to its direct-democratic political system, its geographically inherent land-use trade-offs, and the polarized discussion about agriculture and the expansion of renewable energy on land^18,50,51^. Given Switzerland’s scarce land availability, its strong cultural value of nature, traditional landscapes, and agriculture, and its increasing energy demand, land-use trade-offs are a salient topic – especially concerning energy and food production. The data collection occurred between 3 May and 8 June 2024, in the run-up to a publicly salient national referendum on 9 June 2024 on the Electricity Law, which aimed to accelerate renewable energy deployment, including open-space PV. This timing and context provide strong internal and external validity.

First, to construct the personalized geolocated information treatment, we linked participants’ home address data with a GIS-based dataset that maps the fine-grained, geolocated building and solar power generation potential of agrivoltaics installations in Switzerland^52^. The randomly selected control group participants received generic information about agrivoltaics, whereas the treatment group was additionally shown a personalized map highlighting the realistic potential for building agrivoltaics plants within a defined radius around their home (0–500 m, 500–1500 m, and 1500–4500 m)^52^. Following the personalized information treatment, all respondents answered a factual manipulation check and a series of attitudinal questions concerning agrivoltaics projects in Switzerland, including questions on their support for agrivoltaics projects located near their place of residence. In addition, respondents answered questions on their support for different public policies aimed at expanding agrivoltaics in Switzerland (see the Methods section and Supplementary Information (SI) Section C for full question wording).

Second, to evaluate preferences for different agrivoltaics project and technology designs, participants in both the control and information treatment groups completed a paired conjoint experiment. In four successive choice rounds, respondents were shown two agrivoltaics project design options side by side and asked to indicate their preferred option and the degree to which they supported or opposed each option. Each option was described using seven key project and technology design attributes, with attribute levels randomly assigned (see Table 1 in the Methods section). We selected these design attributes and levels based on a literature review [e.g., refs. ^34–43,46^] and expertise on key solar energy technology design attributes (see details in the Methods section).

**Table 1:** Agrivoltaics technology and project design attributes used in conjoint experiment

Agrivoltaics Design Attributes	Levels
Type of agrivoltaics system	- Greenhouse integration / polytunnel replacement - Vertical on pasture/arable land - Horizontal on pasture/arable land
Size of the agrivoltaics system	- Up to 1 football pitch ( ≈ 1 ha) - Up to 5 football pitches ( ≈ 5 ha) - Up to 10 football pitches ( ≈ 10 ha)
Distance from place of residence	− 0–500 m − 500–1500 m − 1500–4500 m
Ownership structure	- Energy cooperative (e.g., local residents) - Farmers - Landowner - Municipality - Regional energy supplier - External (non-local) investors
Effect on local agricultural crop yield	− 0–5% reduction − 6–10% reduction − 11–20% reduction − 21–40% reduction − 41–80% reduction
Effect on local (municipal) electricity production	− 0–5% increase − 6–10% increase − 11–20% increase − 21–40% increase − 41–80% increase
Effect on farmer income	− 0–5% higher income − 6–10% higher income − 11–20% higher income − 21–40% higher income − 41–80% higher income

Table 1 shows Agrivoltaics technology and project design attributes and randomly varied attribute levels used in the conjoint experiment. We selected these design attributes and levels based on a literature review [e.g., refs. ^36–41,46^] and expert knowledge of key renewable and solar energy project design attributes. Specifically, we included those agrivoltaics project and technology design attributes that we expected to affect support across ideological groups and local residents.

In sum, our study underscores the importance of technology- and project-specific factors—especially the type and size of an agrivoltaics project, its ownership structure, and its impact on food production—in reducing local and right-leaning opposition to land-based solar energy. Furthermore, it highlights that realistic geolocated information about the potential of expanding agrivoltaics close to residents’ homes is a less important factor in shaping public support than technology- and project-specific factors. Our study thus demonstrates the political potential of well-integrated agrivoltaics systems that minimize agricultural yield losses in overcoming local and right-leaning opposition against solar energy on land. In the following, we outline the key results of our study and discuss their implications for future research and policymaking. Please refer to the Methods section (below the Discussion) and the SI for further details.

## Results

**Fig. 2:**
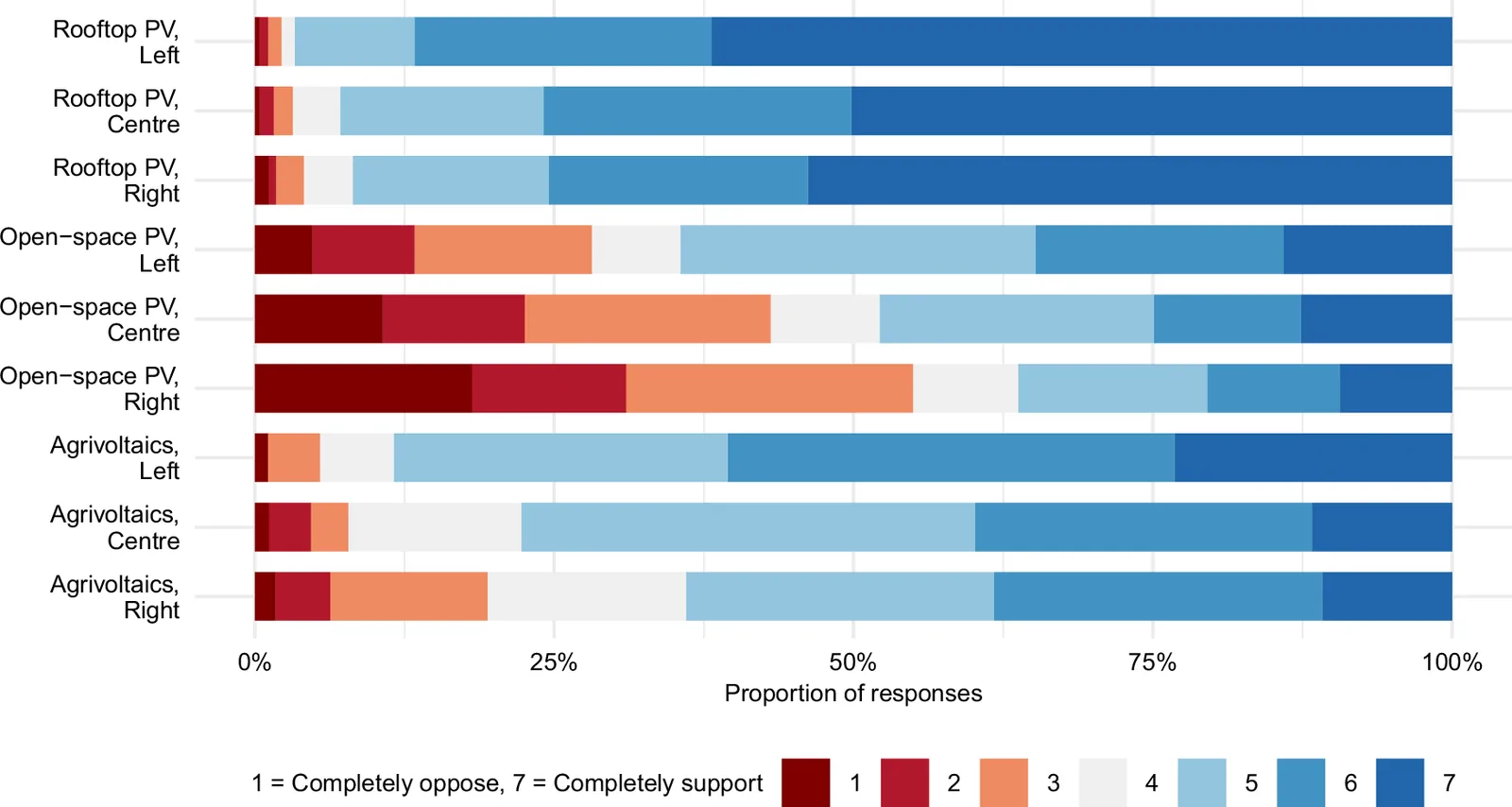
Support for the expansion of different photovoltaic technologies in Switzerland by political orientation. Figure 2 shows the proportion in our survey with a representative sample of Swiss residents with left-leaning (1–4), centric (5–6) and right-leaning (7–10) political ideology who clearly oppose (1) to clearly support (7) open-space PV, rooftop PV and agrivoltaics. Political ideology was measured on a 10-point Likert scale from left (1) to right (10). The survey was conducted in May/June 2024 (see Methods) with *n* = 2145.

Figure 2 shows that around 60 percent of right-leaning residents in Switzerland and more than 50 percent of residents with centrist political views do not support open-space solar projects. In contrast, similar to rooftop solar PV, agrivoltaics projects generally enjoy broad political support among left-, center-, and right-leaning residents in Switzerland. For instance, more than 60 percent of right-leaning residents support agrivoltaics (see Fig. 2).

Moreover, we find similar results for rural areas that are most affected by such projects. While open-space PV projects receive less than 40 percent support among rural residents, agrivoltaics projects receive almost 60 percent support in rural areas (see SI Fig. 5).

### Local expansion potential of agrivoltaics has small effects on public support

**Fig. 3:**
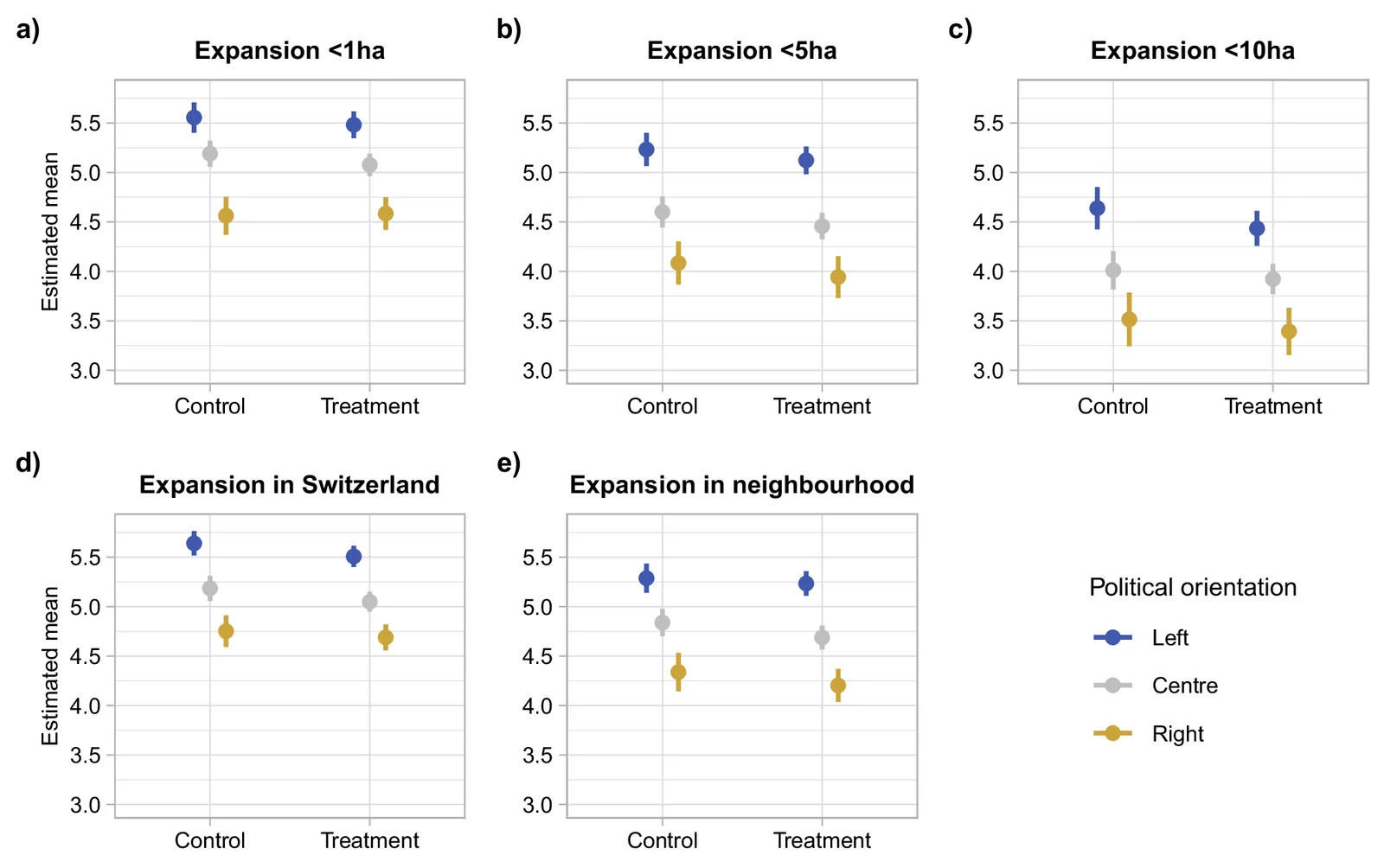
Support for agrivoltaics expansion by size, location, and political orientation. Figure 3a–c present respondents’ support for the expansion of agrivoltaics installations of different sizes, measured on a 7-point Likert scale ranging from 1 (“strong opposition”) to 7 (“strong support”). Project sizes include small-scale (up to one football pitch, ≈ 1 ha) shown in Fig. 3a, medium-scale (up to five football pitches, ≈ 5 ha) shown in Fig. 3b, and large-scale (up to ten football pitches, ≈ 10 ha) installations displayed in Fig. 3c. Figure 3d, e illustrate respondents’ general support for the expansion of agrivoltaics in Switzerland (Fig. 3d) and in their direct residential vicinity (Fig. 3e), again measured on a 7-point Likert scale (1 = “strong opposition”, 7 = “strong support”). For all panels, data points represent the mean support estimates for left-leaning (blue, *n* = 818), centric (gray, *n* = 807), and right-leaning (yellow, *n* = 520) respondents (*n* = 2145) in both the control (*n* = 707) and the information treatment groups (*n* = 1438) based on linear regressions and two-sided tests, with error bars indicating 95% confidence intervals (see details in SI Table 10).

Figure 3a–c reveal that respondents across the ideological spectrum – i.e., left-leaning (blue), centrist (gray), and right-leaning (yellow) respondents – express significantly lower support for the expansion of medium- to large-scale agrivoltaics installations (up to ten football pitches, ≈10 ha) compared to small-scale ( ≈ 1 ha) projects. Large-scale agrivoltaics projects receive substantially lower support among all groups, in particular among right-leaning residents. For right-leaning residents, the estimated mean support rating for large-scale projects on a 7-point scale is 3.5 (see Fig. 3c), while for small-scale projects the estimated mean rating is 4.5 (see Fig. 3a). Overall, a clear ideological divide persists: left-leaning and centrist respondents consistently express greater support for agrivoltaics expansion across all project sizes, whereas support among right-leaning individuals remains comparatively lower. Notably, the geolocated treatment providing respondent-tailored information on the realistic local potential of expanding agrivoltaics close to their homes has only a weak negative support effect for large agrivoltaics projects (but not for smaller projects).

Figure 3d, e reinforce the observed pattern, showing that left-leaning and centric respondents express higher support for expanding agrivoltaics in Switzerland and in respondents’ neighborhoods compared to right-leaning individuals. Again, the geolocated information treatment has only weak and no significant dampening support effects (i.e., −0.123 to −0.155 points on a 7-point Likert scale; see details in SI Section C, SI Table 10).

### Technology and project design key factor for broad agrivoltaics support

**Fig. 4:**
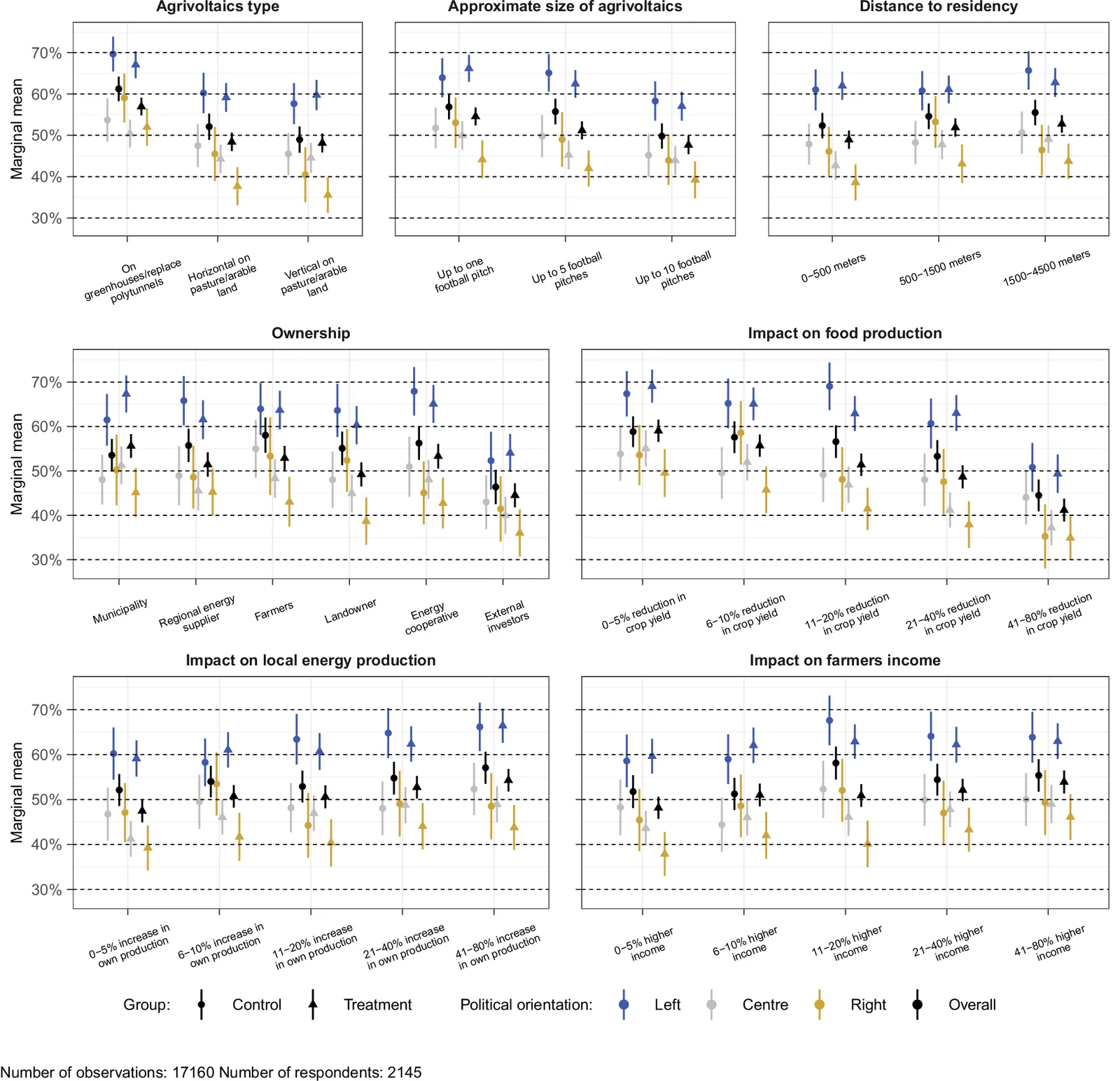
Support for different agrivoltaics project designs by political orientation and treatment group. Figure 4 displays the marginal means for clear support – defined as a support rating greater than 5 on a 7-point Likert scale – for differently designed agrivoltaics projects among the overall sample (black, *n* = 2145), left-leaning (blue, *n* = 818), centric (gray, *n* = 807) and right-leaning (yellow, *n* = 520) respondents in the control (circle, *n* = 707) and information treatment (triangle, *n* = 1438) group, based on the geolocated information and conjoint experiments (see details in Method section). In this case, the marginal means represent the predicted probability that a respondent from a particular subgroup (e.g., left-leaning, centric or right-leaning ideology and control vs. information treatment) expresses clear support for an agrivoltaics design containing a given attribute level. Data points indicate the estimated marginal means for clear support (ranging between 0%-100%) for an agrivoltaics project with the respective design attribute. Vertical lines represent cluster-robust 95% confidence intervals derived from linear least squares regression and two-sided tests. Detailed regression results can be found in SI Section C.

Figure 4 shows a pronounced ideological divide in support for differently designed agrivoltaics projects among the overall sample (black), left-leaning (blue), centrist (gray), and right-leaning (yellow) respondents in the control (circle) and information treatment (triangle) groups, based on the combined geolocated information and conjoint experiments (see further details in the Methods section and SI Table 12). Overall, we find that agrivoltaics project and technology design are crucial factors for reducing ideological and local opposition to land-based solar energy. In contrast, geolocated information about the realistic potential of agrivoltaics projects near respondents’ homes affects support less strongly than project and technology design.

In general, while left-leaning participants show clear support across most agrivoltaics technology and project types—except for scenarios involving substantial crop yield reductions (41–80%)—clear majority support among right-leaning and centric residents depends much more on the specific project and technology design.

For instance, agrivoltaics systems that are well integrated into existing agricultural and landscape structures (e.g., replacing greenhouses or polytunnels) receive clear support from left-leaning, centrist, and right-leaning respondents in the control group (see Fig. 4 and SI Table 12; marginal means for clear support between 54–70 percent). In the information treatment group, clear support for well-integrated agrivoltaics systems is somewhat lower than in the control group but still above 50 percent among all ideological subgroups. Importantly, this is not the case for other types of agrivoltaics systems—especially those that are closer in visual appearance to open-space PV systems—such as horizontal or vertical systems on pasture or arable land. For instance, clear support among right-leaning respondents falls to 40 percent for vertical systems on pasture or arable land in the control group and 36 percent in the information treatment group (see Fig. 4 and SI Table 12).

Similarly, when anticipated crop yield reductions are minimal (0–5%), a majority of respondents across all ideological groups in the control and information treatment groups clearly support agrivoltaics projects. For instance, while clear support among right-leaning respondents in the control group for projects with minimal crop yield reduction is 54 percent, in the information treatment group, 50 percent of right-leaning residents still clearly support such projects (see Fig. 4 and SI Table 12).

Moreover, project size and local ownership foster cross-ideological support. Across ideology, a clear majority of respondents in the control group support small-scale projects (up to 1 football pitch, ≈1 ha). In addition, 53 percent of right-leaning, 55 percent of centrist, and 64 percent of left-leaning respondents in the control group back projects owned by local farmers (see Fig. 4 and SI Table 12).

These findings suggest that key design elements—specifically, system type and visual integration, implications for agricultural output, and ownership structure—are central to securing majority support across the political spectrum. We find similar results when studying these design effects for urban, suburban, and rural residents (see SI Figure 10 and SI Table 25). Generally, support among urban and suburban residents is higher for agrivoltaics than among rural residents. Yet, local opposition from rural residents is unlikely if agrivoltaics projects are well integrated, create minimal crop yield reductions, and have local ownership.

Interestingly, across ideological groups and urban–rural residents, the effect of agrivoltaics projects on local food production emerges as a more critical factor than their impact on local energy output. As discussed above and shown in Fig. 4, minimal decreases in food production (0–5%) attract majority support across ideological and urban–rural respondents, whereas large reductions (41–80%) lead to widespread rejection. By contrast, agrivoltaics projects’ substantial increases in electricity generation in the local municipality positively affect support, but less strongly than agrivoltaics projects that minimize the reduction of local food production. This suggests that concerns related to local food production and security weigh more heavily than considerations related to local energy production in shaping cross-ideological and urban-rural attitudes toward agrivoltaics deployment. In addition, these results underscore opposition to open-space PV on agricultural land and buttress the political importance of the dual land-use function of agrivoltaics projects.

Finally, the personalized geolocated information treatment led to some reduction in overall support for agrivoltaics projects, especially among right-leaning respondents, yet these effects did not lead to substantial changes in majority support. For instance, right-leaning respondents in the treatment group have significantly lower support for horizontal agrivoltaics systems (38 percent) compared to respondents in the control group (46 percent) (see Fig. 4 and SI Table 12). In addition, the approximate size of agrivoltaics projects affects support among right-leaning respondents in the treatment group significantly more than in the control group or among left-leaning respondents. For instance, right-leaning respondents in the control group support medium-scale agrivoltaics projects (up to 5 football pitches, ≈5 ha) at 49 percent, while in the treatment group only 42 percent support such projects.

In sum, despite information effects among right-leaning respondents for some agrivoltaics designs, the information treatment is a less decisive factor in shaping support than agrivoltaics project and technology design.

### Geolocated information unlikely to alter agrivoltaics policy support

**Fig. 5:**
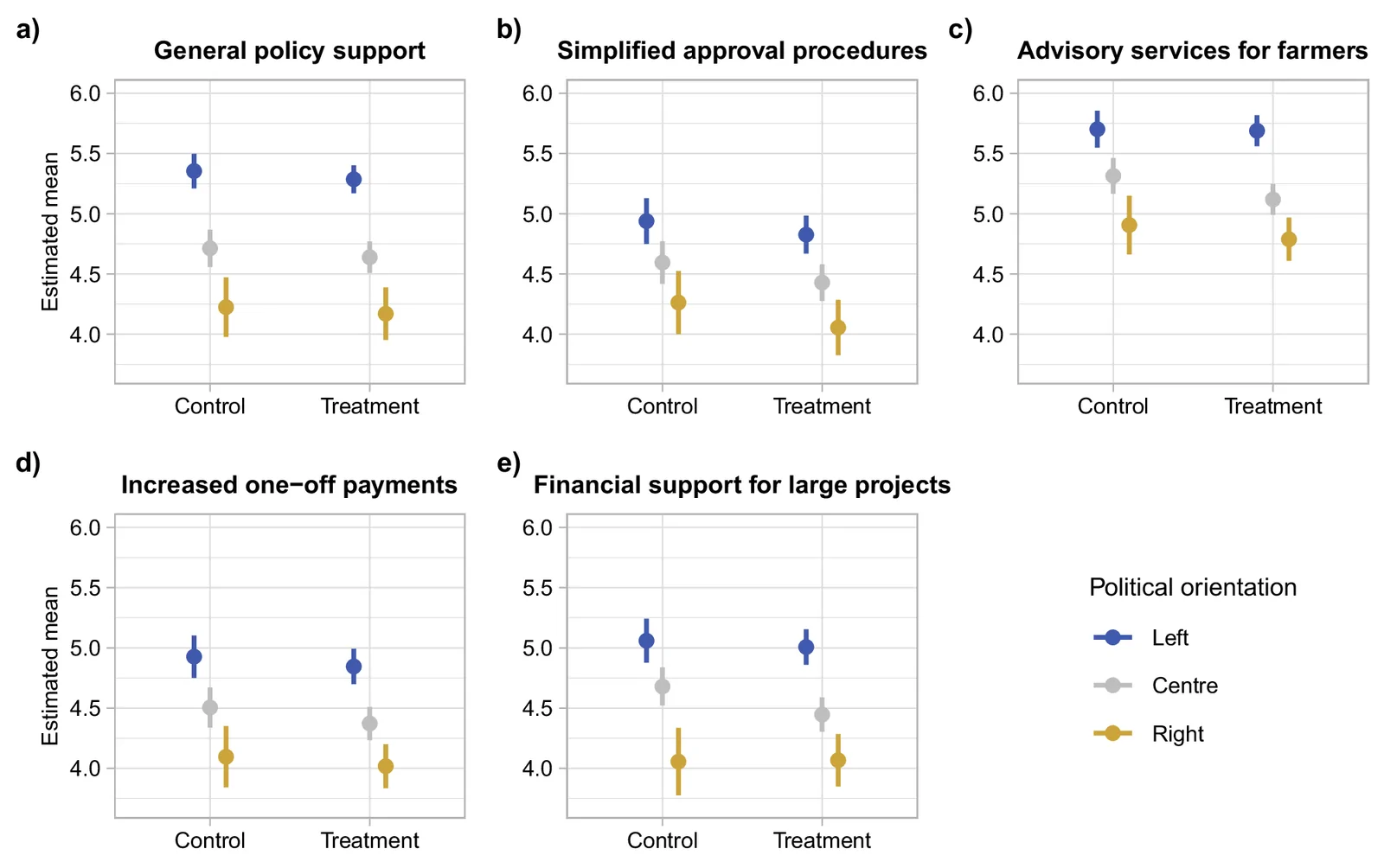
Support for different policy measures aimed at promoting agrivoltaics projects. Figure 5a–e show respondents’ support for various policy measures aimed at promoting the expansion of agrivoltaics in Switzerland, measured on a 7-point Likert scale ranging from 1 (“strong opposition”) to 7 (“strong support”). Figure 5a displays general support for policies expanding agrivoltaics in Switzerland. Figure 6b illustrates support for simplifying approval procedures for agrivoltaics installations. Figure 5c presents support for providing advisory services to farmers to facilitate agrivoltaics adoption. Figure 5d shows support for increasing one-off payments to incentivize agrivoltaics installation. Figure 5e presents support for offering financial assistance specifically for large-scale agrivoltaics projects. For all panels, data points represent the mean support estimates for left-leaning (blue, *n* = 818), centric (gray, *n* = 807), and right-leaning (yellow, *n* = 520) respondents (*n* = 2145) in both the control (*n* = 707) and the information treatment groups (*n* = 1438) based on linear regressions and two-sided tests, with error bars indicating 95% confidence intervals (see details in SI Table 13).

Finally, Fig. 5a–e (see details in SI Section C, SI Table 13) highlight that there is generally cross-ideological support for policies aimed at promoting the expansion of agrivoltaics in Switzerland. However, we again observe a pronounced ideological divide in policy support. While right-leaning and centrist respondents do not oppose policies promoting agrivoltaics (i.e., mean policy support is not lower than 4 on a 7-point Likert scale), left-leaning voters clearly support these policies (i.e., mean policy support is higher than 4.5 on a 7-point Likert scale). Notably, certain policy instruments appear more capable of attracting broad cross-ideological approval. For example, targeted advisory services receive relatively high levels of support across ideological groups (i.e., a mean support of almost 5 for right-leaning, 5.3 for centrist, and 5.6 for left-leaning respondents on a 7-point Likert scale).

However, other policy measures—such as simplified approval procedures, increased one-off payments, or financial incentives for large-scale agrivoltaics projects—garner less support across ideological groups. For instance, financial incentives for large-scale agrivoltaics projects receive a mean support of 4 for right-leaning respondents, 4.7 for centrist respondents, and 5.1 for left-leaning respondents.

Again, the geolocated information treatment does not produce a notable and consistent shift in policy preferences (see SI Section C, SI Table 13).

## Discussion

Our field-embedded survey experiment offers insights into the sociopolitical implications of agrivoltaics—a dual-use technology that combines solar energy production with agricultural activity. We contribute to the literature by studying the public support effects of agrivoltaics projects and technology design, and of personalized information about the realistic potential of building agrivoltaics close to respondents’ homes. In doing so, we leverage a combination of a geolocated information treatment and a conjoint experiment fielded at a unique time, just before a direct-democratic referendum on renewable energy expansion in Switzerland.

Our findings show that—unlike open-space solar energy projects on land (see Fig. 1)—agrivoltaics as a dual land-use technology can reduce political opposition and increase cross-ideological and urban–rural support for solar energy on land.

A first key takeaway from our study is that technology and project design attributes of agrivoltaics—such as their integration with existing agricultural infrastructure, their size, ownership structure, and the impact on crop production—play a crucial role in shaping support across ideological groups and urban–rural populations. Left-leaning and urban residents consistently show stronger support for agrivoltaics, but majorities—even among right-leaning and rural populations—emerge when installations are landscape integrated, not large scale, locally owned, and do not result in substantial yield reductions. This highlights the potential of agrivoltaics—compared to open-space solar energy—as a politically viable renewable energy solution, especially in polarized political contexts.

A second key takeaway from our experiment is that the personalized geolocated information has modest support effects and only interacts with some agrivoltaics technology and project design attributes. Comparing the effect sizes of the geolocated information treatment with those of project-technology design across ideological groups and urban–rural populations, it is evident that design effects are larger. Our results thus show that while partisan polarization, urban–rural divides, and negative effects from information about local expansion potential are present, these effects can be mitigated through smart project and technology design.

For policymakers, these results suggest that well-designed agrivoltaics projects have the potential to overcome local opposition and foster cross-ideological support for solar energy expansion on land. To unlock this potential, policymakers must carefully consider key design elements, especially project scale, landscape integration, and ownership structure. In addition, we find that citizens are particularly sensitive to the impact of land-based solar projects on local food production, whereas they are less concerned about impacts on local energy production. This underscores the importance for policymakers of promoting agrivoltaics projects that minimize crop yield losses to garner cross-ideological and local support. Moreover, policies that align agrivoltaics with existing agricultural practices, reduce visual disruption, and enable local economic participation are likely to gain broad support across diverse groups.

Finally, policymakers may face a trade-off between minimizing political opposition and containing economic costs when expanding land-based solar energy. As this study demonstrates, political risks can be mitigated by promoting agrivoltaics, particularly when systems are smaller scale and carefully integrated into the landscape – for instance, by replacing existing structures such as greenhouses or polytunnels –, and when they do not significantly reduce agricultural yields. However, levelized electricity costs for agrivoltaics vary depending on design, ranging from approximately 10% higher than conventional open-space PV for low-mounted horizontal systems to as much as 50% higher for elevated structures positioned more than 4 meters above the ground^52^. Especially, large, low-mounted horizontal systems can benefit from economies of scale and lower customization costs than smaller, more integrated agrivoltaics systems. Landscape-integrated agrivoltaics systems positioned up to 2.5 m above ground (see Fig. 1a) typically incur a 30–40% cost premium^52^. This constitutes a potential trade-off for policymakers between efficiency and feasibility: Large-scale open-space PV and low-mounted horizontal agrivoltaics systems are more cost-efficient to reach renewable energy targets but are publicly less accepted—especially among right-wing and rural residents—than smaller, more integrated agrivoltaics systems. Yet these more-integrated designs can offset part of their higher costs by reducing yield losses from extreme weather events (e.g., hail) and substituting for other farm infrastructure (e.g., polytunnels). Moreover, while well-integrated systems currently do raise electricity generation costs compared to alternative open-space and agrivoltaics systems, they can deliver clear political co-benefits that may reduce investment risks. It is also likely that, due to learning effects, costs—especially for well-integrated systems—may fall further over time. Notably, we find that public support for well-integrated designs with minimal impact on crop yield is up to 40–50 percentage points higher than for large-scale open-space PV, vertical, or horizontal agrivoltaics installations that lead to higher yield losses (see SI Section C). These results suggest that, depending on sociopolitical conditions, the political advantages of well-integrated agrivoltaics designs may outweigh their higher levelized electricity costs compared to open-space PV, particularly if such projects help forge new coalitions, including skeptical right-leaning voters and farmers.

The political potential of agrivoltaics is already apparent in practice: While the Italian conservative government under Prime Minister Giorgia Meloni has imposed restrictions on open-space PV installations to preserve farmland, it simultaneously promotes agrivoltaics as a more acceptable alternative^12^. Future research should further explore such cases to examine how agrivoltaics may open new pathways for building unexpected political coalitions in support of solar energy.

While this study makes important contributions to understanding the sociopolitical dynamics of agrivoltaics deployment, several limitations suggest promising avenues for future research. First, the study is based on the Swiss context, which is characterized by direct-democratic institutions, strong land competition, and polarized political dynamics around climate policies and renewable energy^18,50,51^. Moreover, Switzerland is a high-income country with—compared to other countries—large subsidies to farmers^53,54^. This context might explain some of the results, for instance, the limited public support for additional financial support to farmers. Therefore, the extent to which our findings can be generalized to other countries with different institutional structures, land-use pressures, economic and political environments remains an open question. Second, although our focus has been on the political implications of agrivoltaics as a recently developed dual land-use technology, future studies should explore how geolocated information and project and technology design affect public support across different types of low-carbon technologies. This comparative work would contribute to a broader understanding of technology politicization and how different technology-specific attributes influence polarization and local opposition in low-carbon energy transitions. Third, future research could study how project and technology design can be strategically utilized in policy design and how this might feed back into later policymaking, for instance, by changing interest group and voting behavior.

In conclusion, this study highlights the political importance of technology design and the potential of agrivoltaics as a dual land-use technology for overcoming ideological division and local opposition to solar energy expansion on land.

## Methods

### Survey panel and case selection

The respondents in our survey are drawn from the population register of the Federal Statistical Office (BfS), comprising Swiss residents. The BfS population register mirrors, aside from random error and uneven response rates, the Swiss resident population. We used stratified random sampling with the strata language region (i.e., German- or French-speaking cantons of Switzerland), gender (i.e., male or female), and urbanization level (i.e., urban, semi-urban, rural). We invited a random sample of 16,500 Swiss residents in the French- and German-speaking cantons of Switzerland above the age of 18 years via letter (plus one reminder) to participate in our online survey via the survey platform Qualtrics. We received a total of 3384 responses, of which 445 responses were incomplete, resulting in a response rate of around 20.5%. After excluding respondents who completed the survey in less than 40 percent of the median response time (44 min), as well as unclear responses and non-completes, our final sample consisted of 2145 Swiss residents. As shown in SI Section B, our random sample is a close approximation of the Swiss resident population in the French- and German-speaking cantons of Switzerland above the age of 18 years. Younger respondents (ages 18–33) are slightly underrepresented, while older respondents (ages 58–78) are slightly overrepresented in your survey.

The data collection occurred between May 3 and June 8, 2024, in the run-up to a publicly salient national referendum on the Electricity Law, which aimed to accelerate renewable energy deployment, including open-space PV. The unique timing and context of the direct-democratic referendum provide strong external validity given the increased issue salience and voters’ direct say in political decision-making.

In addition, Switzerland is a particularly relevant case study due to its direct-democratic political system, geographic conditions, and cultural values. Citizens routinely vote on land-use and energy issues, such as the Electricity Law, amplifying the role of public support in actual political decision-making. The country’s mountainous geography and scarcity of arable land create tensions between agriculture and renewable energy infrastructure. These tensions are often amplified in polarized political discussions between right-leaning and left-leaning parties and by influential lobby groups, such as the powerful Swiss farmer association. Moreover, Swiss citizens place high value on landscape preservation as well as food and energy self-sufficiency^55^. Additionally, most research on local opposition to renewable energy has focused on the United States and United Kingdom^14^, while Switzerland’s increasingly polarized political climate around climate policy and renewable energy^50,51^ makes it an ideal context for studying ideological and urban-rural variation in renewable energy, especially agrivoltaics, support.

### Experimental design

For our pre-registered experiment (see pre-registration details^56^), we developed a GIS-based dataset that maps the fine-grained, geolocated building and solar power generation potential of agrivoltaics installations in Switzerland^52^.

The dataset is based on^52^, which shows the geolocated technical potential for agrivoltaics in Switzerland. It includes agricultural land located no more than 1000 m from a building zone and potentially suitable for agrivoltaics. This is consistent with the current regulatory framework in Switzerland and accounts for grid connection. Areas with low horizontal irradiation (less than 1000 kWh/m2) were excluded, as were summer grazing areas and areas that are obviously unsuitable for agrivoltaics. Furthermore, areas in national protected zones such as BLN, national parks, or moorlands were classified as unusable. The remaining technical potential is available as a polygon dataset and can thus be narrowed down to any geographical region.

**Fig. 6:**
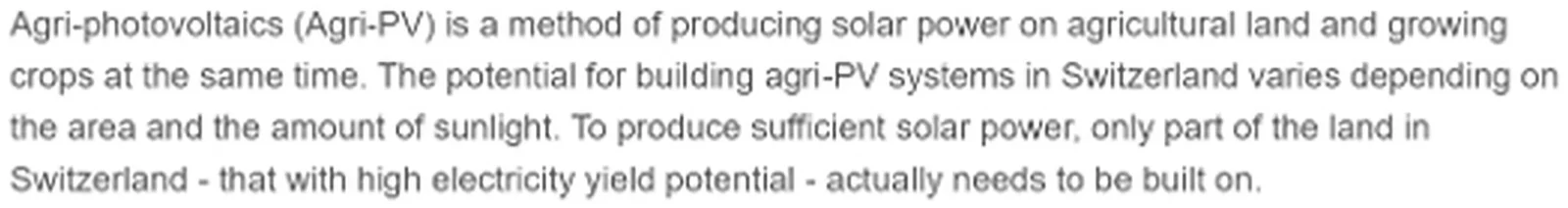
Example of control group information. Figure 6 shows an example of the information provided to the control group.

**Fig. 7:**
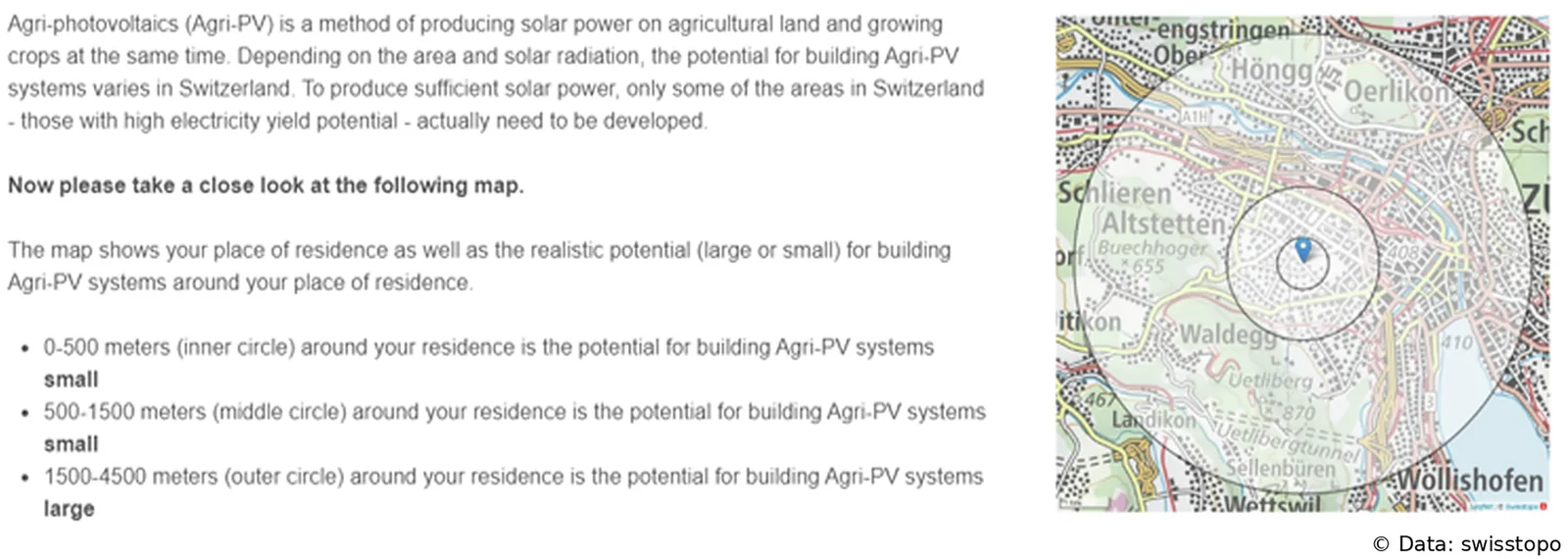
Example of personalized and geolocated information treatment. Figure 7 shows an example of the personalized and geolocated information provided to the treatment group. The personalized map shown to treated respondents highlighted the realistic potential for building agrivoltaics plants around respondents’ home within a defined radius (0–500 m, 500–1500 m, and 1500–4500 m). The map is based on data provided by © Data: swisstopo.

This polygon dataset was combined with participants’ home address data from the Swiss Federal Statistical Office, allowing us to construct an individualized information treatment condition. The randomly selected control group of participants received generic information about agrivoltaics shown in Fig. 6, while the treatment group was additionally shown a personalized map highlighting the realistic potential for building agrivoltaics plants around their home within a defined radius (0–500 m, 500–1500 m, and 1500–4500 m)^52^, as shown in Fig. 7. The realistic potential (GWh/a) within each pre-defined radius was either displayed as large (upper 1/3 of the distribution of solar power generation potential of all respondents in the respective radius) or small (lower 2/3 of the distribution of solar power generation potential of all respondents in the respective radius). Our balance checks (see SI Section C) indicate that the treatment randomization worked as expected and the control and treatment samples are well balanced.

Following the information treatment, in a factual manipulation check, all respondents in both the control and treatment groups were asked several questions regarding their perceived realistic potential of building agrivoltaics installations in proximity to their home (see SI Section C for exact question wording). These items allowed us to show that respondents understood the content of the information treatment and that the treatment had the expected information-updating effects on respondents’ knowledge about realistic agrivoltaics installation potential near their home. As described in SI Section C, our analysis of the factual manipulation and comprehension check items validates that respondents in the information treatment group (compared to the control group) significantly updated their knowledge about realistic agrivoltaics building potential in line with the information treatment received (see SI Section C, SI Table 5–8).

After the factual manipulation and comprehension check items, the map containing the agrivoltaics potential was presented again to the treatment group to remind respondents about the realistic agrivoltaics installation potential before assessing the information treatment effect on a series of attitudinal 7-point Likert scale questions. These dependent variables concerned the perceived advantages of agrivoltaics projects in Switzerland and of those located near their place of residence, as well as respondents’ support for the expansion of agrivoltaics projects of different sizes and locations (see the codebook in SI Section C for exact question wording). In addition, respondents answered questions on their support for different public policies aiming to expand agrivoltaics in Switzerland (see SI Section C for exact question wording).

After the personalized geolocated information treatment, we fielded a conjoint experiment to assess the support effects of the information treatment for differently designed agrivoltaics projects across ideological and rural–urban populations. First, we introduced to respondents all agrivoltaics’ technology and project design attributes used in the conjoint experiment (see Method Tab). We selected these design attributes based on a literature review [e.g., refs. ^36–41,46,57^] and expert knowledge of key renewable and solar energy project design attributes. Specifically, we included those agrivoltaics project and technology design attributes that we expected to affect support across ideological groups and local residents. Second, we then implemented four successive choice rounds in which respondents were shown two agrivoltaics project profiles side by side and asked to indicate both their preferred option and the degree to which they supported or opposed each option on a 7-point Likert scale (see exact wording in SI Section C). Each agrivoltaics project option was described using the seven design attributes, with attributes fully randomized within and across agrivoltaics project options. This allows the non-parametric identification of causal effects of the attributes. Respondents then Conjoint experiments work by randomly combining different levels of attributes (in our case related to differently designed agrivoltaics projects) into full-profile options and then asking respondents to choose between two such options^58–60^. Because participants weigh all attributes simultaneously, the exercise mirrors the multidimensional trade-offs people make in real-world decisions more closely than single-attribute rating tasks^58–60^. This advantage has led to a rapid rise in its use to study public preferences across the social sciences in recent years^58–63^, including in the context of renewable energy^2^. Evidence shows that conjoint designs deliver findings with strong external and ecological validity and are less prone to social desirability bias than many alternative survey techniques^58,59^.

### Analysis of geolocated information treatment

We quantify the effect of the personalized and geolocated information treatment on every attitudinal and policy-support outcome with ordinary least-squares (OLS) regressions estimated on the original 1–7 Likert responses. Robust heteroskedasticity-consistent (HC2) standard errors are reported.

For respondent $i\in {{{\mathscr{I}}}}=\{i,\ldots,N\}$, the estimating equation is:

1 $${Y}_{i}={{{{\rm{\beta }}}}}_{0}+{{{{\rm{\beta }}}}}_{1}{T}_{i}+{{{{\rm{\beta }}}}}_{2}{G}_{i}+{{{{\rm{\beta }}}}}_{3}\left({T}_{i}\times {G}_{i}\right)+{X}_{i}^{{{\top }}}{{{\rm{\gamma }}}}+{{{{\rm{\varepsilon }}}}}_{i} \quad \forall i{{{\mathscr{\in }}}}{{{\mathcal{J}}}},{G}_{i}\in \{{{{\mathcal{P}}}},{{{\mathcal{U}}}}\}$$

where

- ${Y}_{i}$ denotes the respective attitudinal and policy support outcome,
- ${T}_{i}$ equals 1 if respondent i received the personalized treatment, 0 otherwise,
- ${G}_{i}\in \{{P},{U}\}$ is either respondents’ self-identified ideology variable ${P}=\{{{{\rm{Left}}}},{{{\rm{Center}}}},{{{\rm{Right}}}}\}$ or urban-rural categorical variable ${U}={\{{{\rm{Urban}}}},{{{\rm{Suburban}}}},{{{\rm{Rural}}}}\}$,
- ${T}_{i}\times {{G}}_{{i}}$ is the interaction between treatment and ideology/urban-rural variable,
- ${{{{\bf{X}}}}}_{i}$ is the vector of control variables (age, gender, environmental score, prior feelings about agrivoltaics, prior opinion about agrivoltaics, prior familiarity with agrivoltaics, degree of urbanization, and realistic agrivoltaics potential in three distance bands),
- ${{{\boldsymbol{\gamma }}}}$ is the vector of coefficients corresponding to the control variables,
- ${{{{\rm{\varepsilon }}}}}_{i}$ is the error term.

The coefficient ${{{{\rm{\beta }}}}}_{3}$ captures how the treatment effect varies by the respective subgroup.

As a robustness test, ordered-logit and ordered-probit specifications were also estimated with substantively similar results.

In addition, we explored the interaction effects between the treatment and the displayed potential (low/high) in each of the three circles (for further details, please see SI Section C).

### Conjoint-experiment analysis

First, we estimate the effects of each agrivoltaics attribute level on public support by calculating Average Marginal Component Effects (AMCEs). The AMCEs measure the average causal effect of an attribute level on support for an agrivoltaics project profile relative to a baseline, holding all other attributes constant^58^.

The conjoint experiment is conducted among all N respondents, that are each completing K=4 choice rounds where J=2 different agrivoltaics projects are presented. Choice rounds are indexed by $k\in \{1,\ldots,K\}$, and the different projects presented per round by $j\in \{1,\ldots,J\}$. Each project is characterized by L attributes, where attribute $l\in \{1,\ldots,L\}$ takes ${D}_{l}$ discrete levels indexed by $d\in \{1,\ldots,{D}_{l}\}$. The selection of attribute level l is fully randomized.

To estimate baseline AMCEs, we use ordinary least squares regressions with standard errors clustered at the respondent level to account for repeated observations. The dependent variable is coded as a binary indicator:

${Y}_{{ijk}}=1$ if respondent i in one of the repeated conjoint experimental choice rounds k rates profile j with clear support (Likert scale values 6 or 7), and 0 otherwise.

This binary operationalization of support aligns with previous research^62,63^ and provides a conservative, robust measure that facilitates intuitive interpretation of the proportion of respondents expressing clear support for different agrivoltaics projects.

The baseline AMCE estimation is done by regressing respondent’s i's rating ${Y}_{{ijk}}$ for a specific project over ${W}_{{ijkld}}$, a dummy variable that is 1 when respondent i got shown project j in round k, where attribute l takes level d, and 0 otherwise:

2 $${Y}_{{ijk}}={{{\rm{\alpha }}}}+{\sum}_{l=1}^{L} {\sum}_{d=1}^{{D}_{l}-1}{{{{\rm{\beta }}}}}_{{ld}}\cdot {W}_{{ijkld}}+{{{{\bf{X}}}}}_{i}^{{{\top }}}{{{\boldsymbol{\gamma }}}}+{{{{\rm{\varepsilon }}}}}_{{ijk}}$$

${{{\rm{\alpha }}}}$ is the intercept, ${{{{\bf{X}}}}}_{i}^{{{\top }}}$ is a vector of control variables, and ${{{\boldsymbol{\gamma }}}}$ is the corresponding vector of coefficients. ${{{{\rm{\varepsilon }}}}}_{{ijk}}$ is the error term with standard errors clustered by respondent i to account for within-respondent correlation across choice rounds. ${{{{\rm{\beta }}}}}_{{ld}}$ is the AMCE of a specific level d in comparison to an omitted baseline level (hence why the second sum runs from d=1 to ${D}_{l}-1$) representing the average change in rating when an agrivoltaics project includes level d rather than the baseline level.

To examine preference heterogeneity, we split the sample based on a respondents’ self-identified political orientation ${{{\mathscr{P}}}}=\{{Left},{Center},{Right}\}$ or degree of urbanization ${{{\mathscr{U}}}}=\{{Urban},{Suburban},{Rural}\}$ of the area the respondent is living in. For each subsample, we split again by ${{{\mathscr{T}}}}=\{{Control},{Treatment}\}$, the treatment group a respondent is in. We estimate (2) separately for all resulting subsamples ${{{{\mathscr{I}}}}}_{g,t}$ (12 in total):

3 $${Y}_{{ijk}}={{{{\rm{\alpha }}}}}^{g,t}+{\sum }_{l=1}^{L}{\sum }_{d=1}^{{D}_{l}-1}{{{{\rm{\beta }}}}}_{{ld}}^{g,t}\cdot {W}_{{ijkld}}+{{{{\bf{X}}}}}_{i}^{{{\top }}}{{{{\boldsymbol{\gamma }}}}}^{g,t}+{{{{\rm{\varepsilon }}}}}_{{ijk}} \quad\forall \left(g,t\right)\in \left({{{\mathcal{P}}}}{{{\mathscr{\cup }}}}{{{\mathcal{U}}}}\right){\times }{{{\mathcal{T}}}},i\in {{{{\mathcal{J}}}}}_{g,t}$$

Comparing AMCEs across population subgroups depends on the ratings of projects with the baseline level of a project attribute of interest. To be able to compare preferences across different groups of respondents, we follow Leeper et al. ^61^ and estimate marginal means (MM), the absolute level of support of a project a specific attribute level, while averaging over all other attributes and covariates.

The AMCE for attribute l at level d (${{{{\rm{\beta }}}}}_{{ld}}^{g,t}$) in subsample ${{{{\mathscr{I}}}}}_{g,t}$, estimated in (3), is now used to calculate the marginal mean, again for all subsamples:

4 $$\widehat{{MM}}\left({m}_{{ld}}{|g},t\right)={\widehat{\upalpha }}^{g,t}+{\widehat{\upbeta }}_{{ld}}^{g,t}+{\left({\widehat{{{\mathbf{\beta }}}}}_{-l}^{\,g,t}\right)}^{{{\rm{T}}}}{\bar{{{\bf{D}}}}}_{{{g}},t}^{-l}+{\bar{{{\bf{X}}}}}_{g,t}^{{{\rm{T}}}}{\widehat{{{\boldsymbol{\gamma }}}}}^{\,g,t}$$

${\hat{{{{\rm{\alpha }}}}}}^{g,t}$ is the estimated intercept from Eq. (3). ${\hat{{{{\rm{\beta }}}}}}_{{ld}}^{g,t}$ is the estimated AMCE for the attribute level of interest ${m}_{{ld}}$; it equals zero when d is the baseline level. ${\hat{{{{\boldsymbol{\beta }}}}}}_{-l}^{g,t}$ is the vector of all estimated attribute coefficients from Eq. (3) excluding those for attribute l. ${\bar{{{{\bf{D}}}}}}_{g,t}^{-l}$ is the vector of observed attribute level proportions across all projects shown to all respondents in ${{{{\mathscr{I}}}}}_{g,t}$, that is the likelihood of each level being part of an agrivoltaics project. The product of ${\hat{{{{\boldsymbol{\beta }}}}}}_{-l}^{g,t}$ and ${\bar{{{{\bf{D}}}}}}_{g,t}^{-l}$ averages the contributions of all attributes except attribute level l over their empirical distribution within the subgroup, thereby marginalizing them out of the estimation. ${\bar{{{{\bf{X}}}}}}_{g,t}$ is the vector of covariate means within ${{{{\mathscr{I}}}}}_{g,t}$, and its product with ${\hat{{{{\boldsymbol{\gamma }}}}}}^{g,t}$ produces a covariate-adjusted estimation of the marginal means. We include covariates to enhance the efficiency of the estimations.

In addition, our results are robust to an alternative modeling strategy: we nonparametrically estimate Average Marginal Interaction Effects (AMIEs) using ANOVA regression with weighted zero-sum constraints, following Egami and Imai^64^. This method enables the estimation of support for complex multi-attribute profiles by accounting for higher-order interactions between attribute levels, independent of baseline category choices. It also allows us to identify the most and least preferred agrivoltaics project configurations across the full factorial design space (see SI Section C, SI Figure 12 and SI Tables 31 and 32).

### Ethics declarations

The study has been pre-registered (*AEA RCT Registry* https://doi.org/10.1257/rct.13540-1.0) and approved by the ETH Zurich Ethics Commission (EK 2024-N-84, Zurich, 24 April 2024).

### Reporting summary

Further information on research design is available in the Nature Portfolio Reporting Summary linked to this article.

## Supplementary information

Supplementary Information

Reporting Summary

Transparent Peer Review file

## Data Availability

The datasets generated by the survey research during and/or analyzed during the current study are available at Github under https://doi.org/10.5281/zenodo.20266276

## Code availability

Code supporting this study (including replication code for analyses, figures and tables) is available on Github under https://doi.org/10.5281/zenodo.20266276. Software The analysis for this paper has been conducted using R version 4.5.2. The following packages have been used: • cregg 0.3.7. • tidyverse 2.0.0. • ggpubr 0.6.2. • kableExtra 1.4.0. • stringi 1.8.7. • cowplot 1.2.0. • FindIt 1.3.0. • estimatr 1.0.6. • patchwork 1.3.2. • texreg 1.39.5. • emmeans 2.0.1. • scales 1.4.0. • vtable 1.4.8. • openxlsx 4.2.8.1. • dplyr 1.1.4
