# Supplementary material for: Agrivoltaics can reduce political polarization and local opposition to solar energy on land
Source: Nat Commun. 2026 Aug 27;17:10263. doi: 10.1038/s41467-026-77141-8 (PMC13616971; doi:10.1038/s41467-026-77141-8)
Supplement: Supplementary file 1 [file 41467_2026_77141_MOESM1_ESM.pdf]

Supplementary Information for "Agrivoltaics can reduce political polarization and local opposition to solar energy on land"

Lukas Fesenfeld, Leon Sistek, Simon Montfort, Dionis Anderegg, Jürg Rohrer, Tobias Schmidt

Corresponding author: Lukas Fesenfeld, ETH Zurich, lukas.fesenfeld@gess.ethz.ch

Contents

Section A - Survey sample 1

Section B - Descriptive statistics 2

Section C - Regression results and additional figures 6

Section C1 – By political orientation . . . 6

Section C2 – By degree of urbanization . . . 33

Section D - Codebook and survey instrument 67

SI Section A - Survey sample

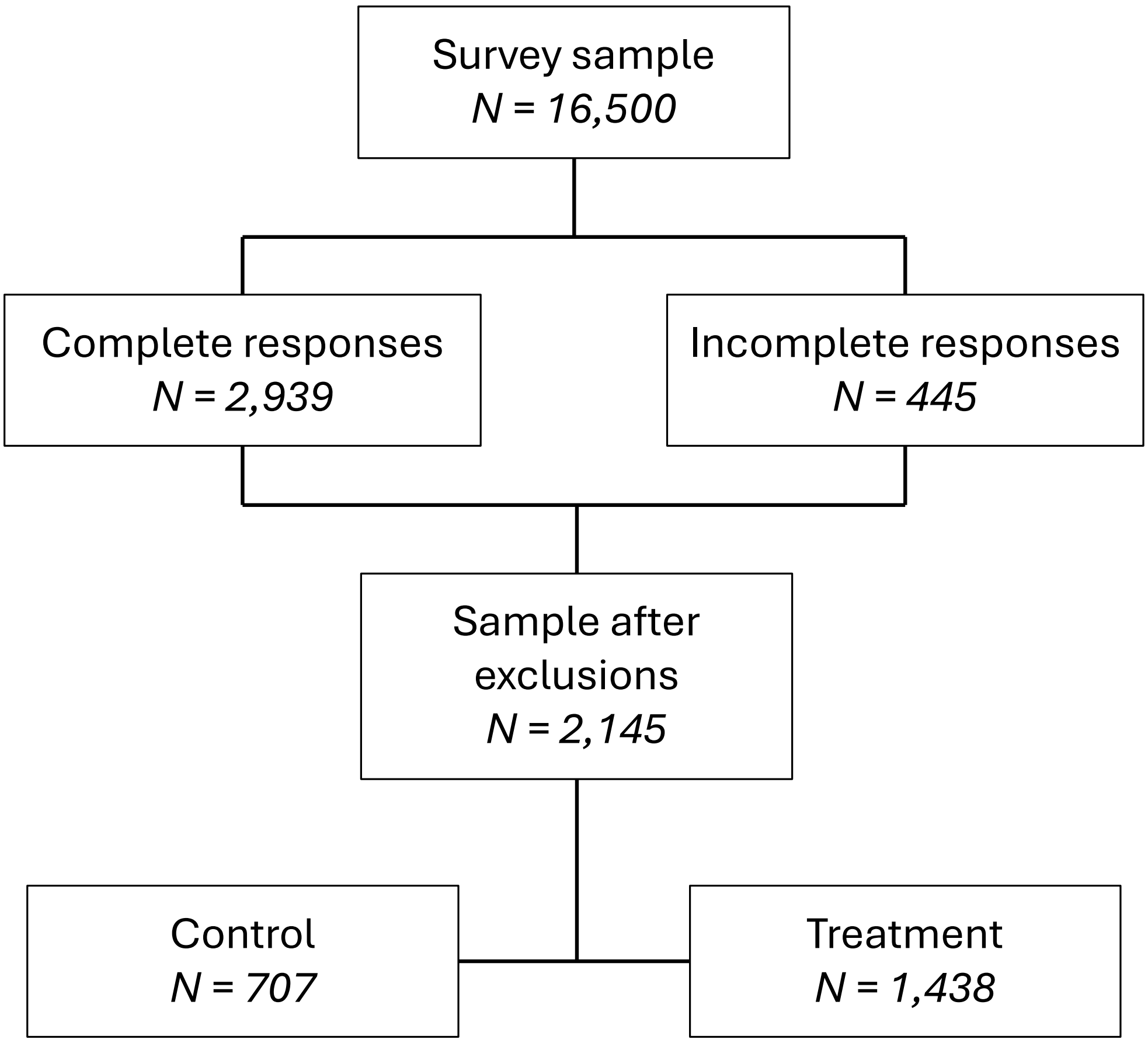

SI Figure 1: Respondents were excluded from the sample if any of the following criteria applied: (a) decided not to participate in the beginning of the survey, (b) answered after 08.06.2024, (c) quit survey before all relevant questions got answered, (d) completed the survey in less than 40% of the median response time, (e) Answered all elements of matrix question on Swiss electricity supply in a straight line (e) younger than 18 years or older than 78 years (f) indicated gender other than male or female.

SI Section B - Descriptive statistics

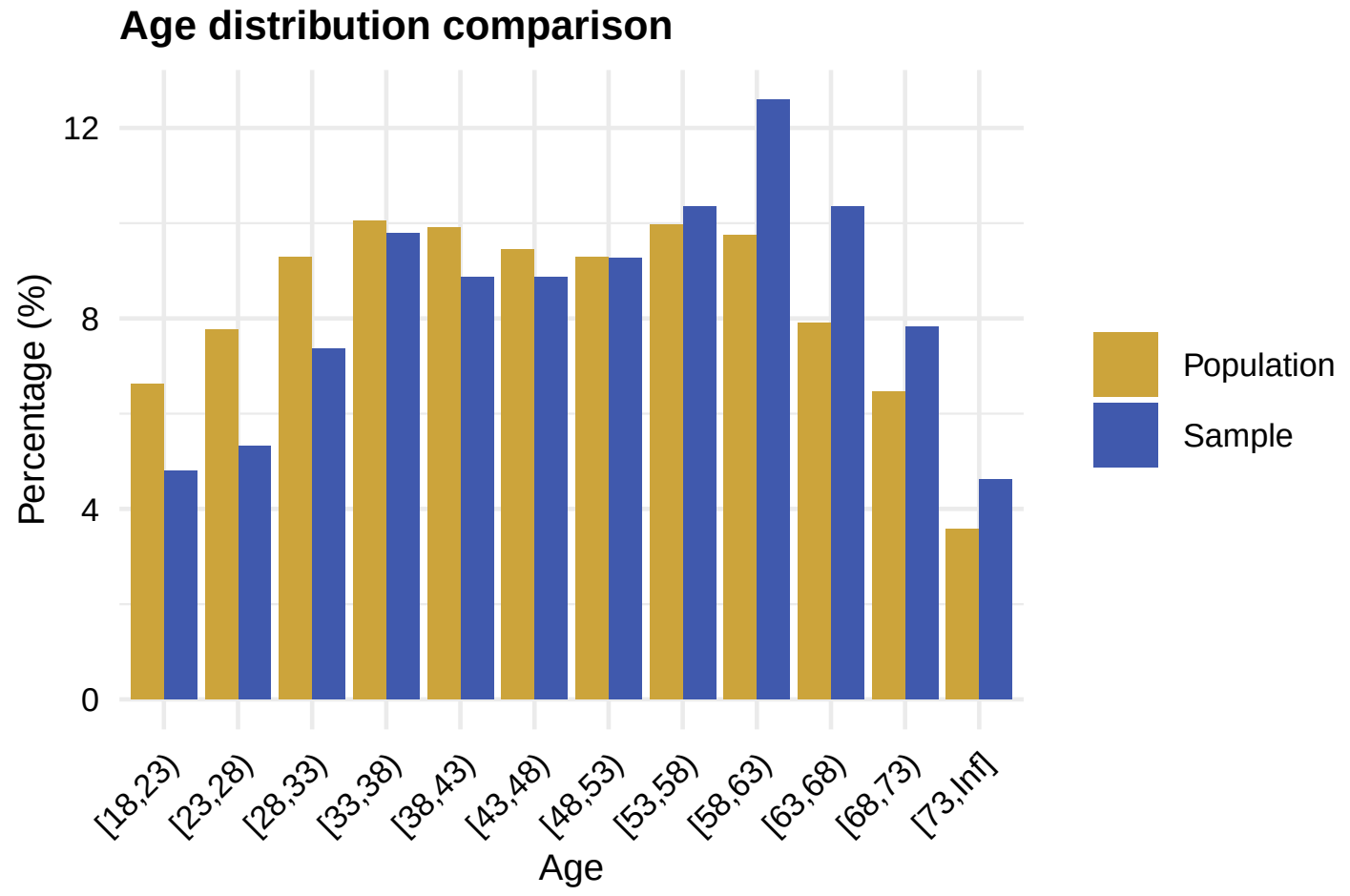

SI Figure 2: Comparison of population and sample statistics (n = 2145) regarding age. The population statistics include all residents from the French and German speaking part of Switzerland.

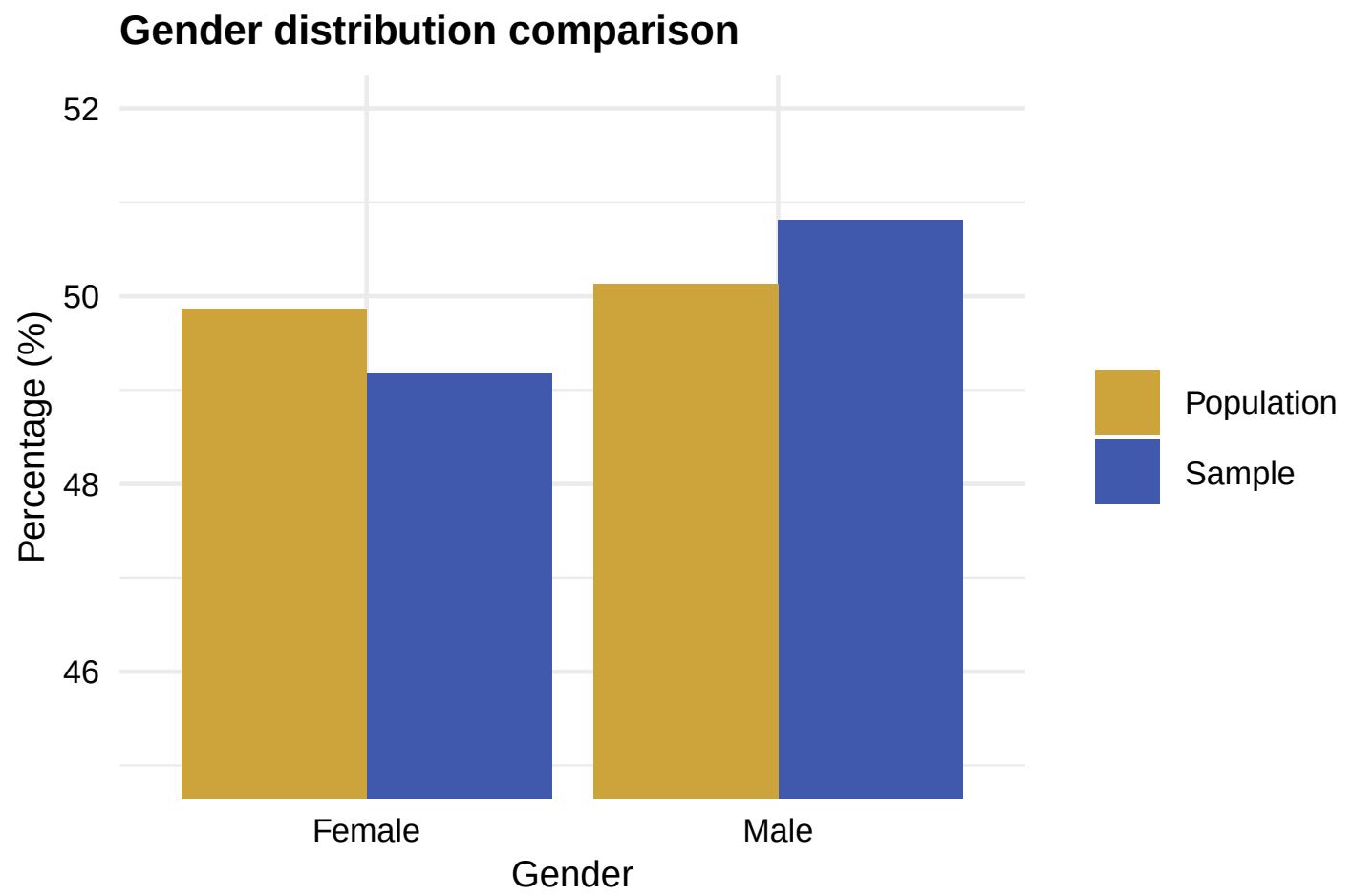

SI Figure 3: Comparison of population and sample statistics (n = 2145) regarding gender. The population statistics include all residents from the French and German speaking part of Switzerland.

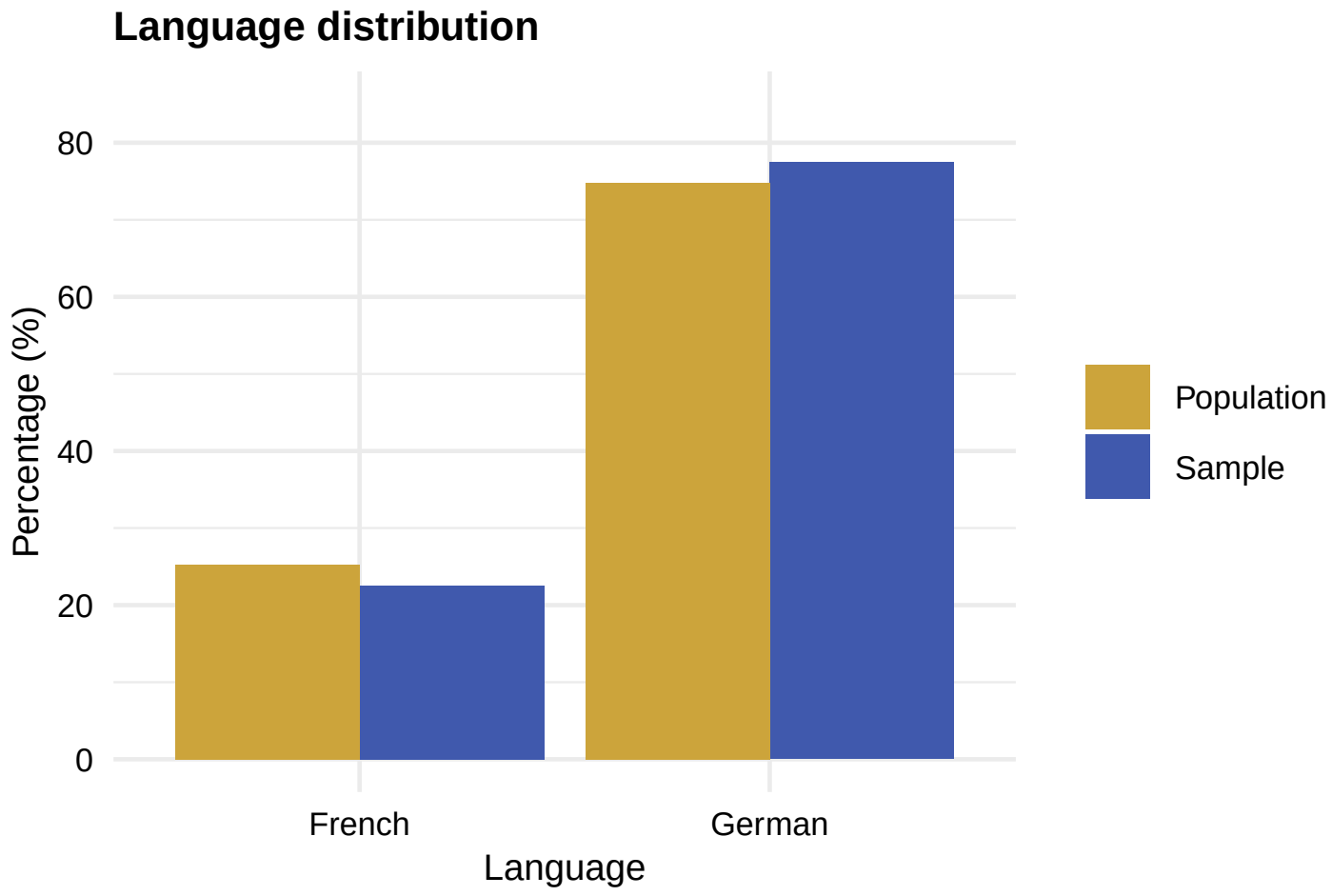

SI Figure 4: Comparison of population and sample statistics (n = 2145) regarding the spoken language. The population statistics include all residents from the French and German speaking part of Switzerland.

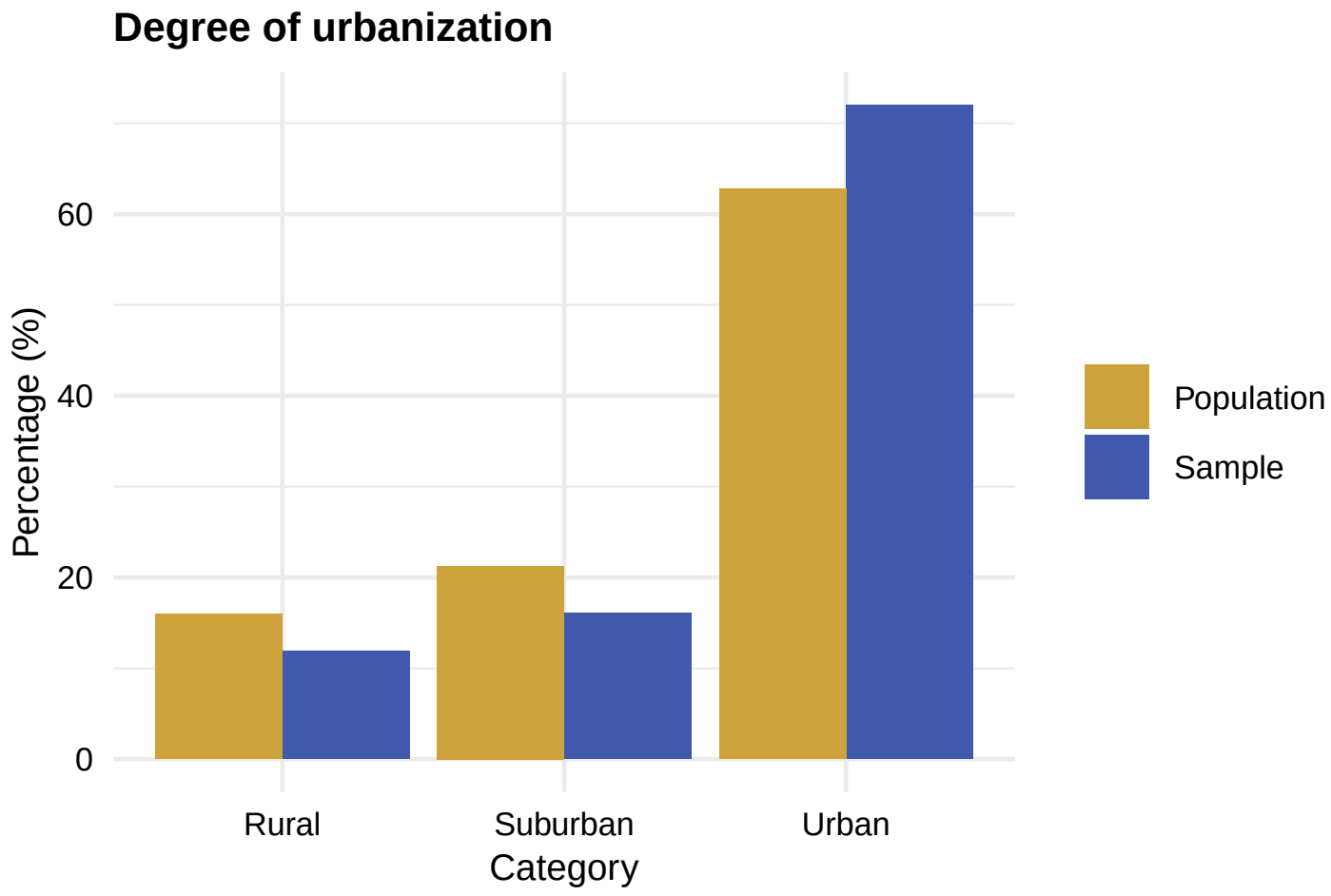

SI Figure 5: Comparison of population and sample statistics (n = 2145) regarding the degree of urbanization. The population statistics include all residents from the French and German speaking part of Switzerland.

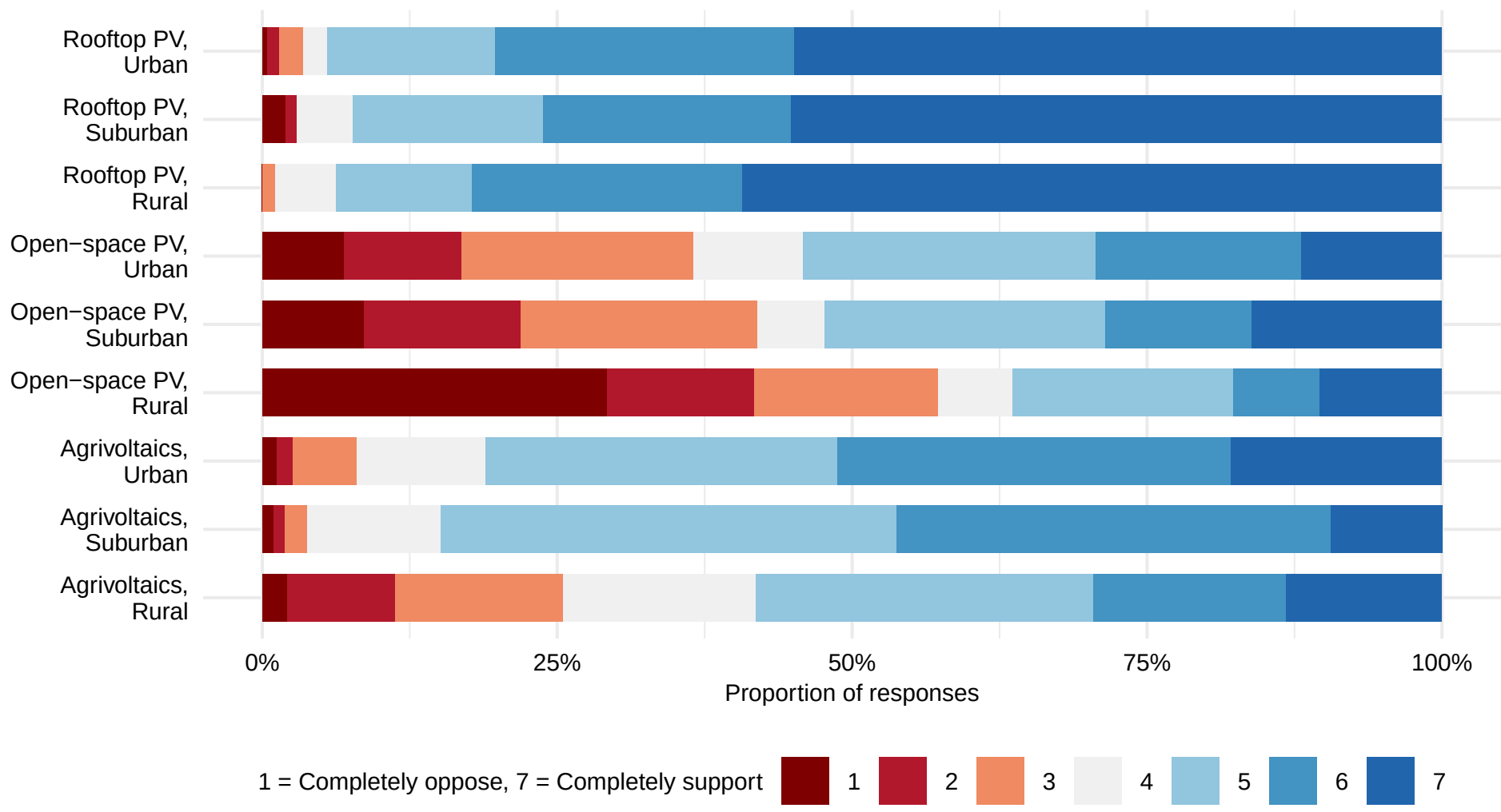

SI Figure 6: Proportion of respondents in our survey with a representative sample of Swiss residents that live in a rural, suburban or rural environment that clearly oppose (1) to clearly support (7) open-space PV, rooftop PV and agrivoltaics. The survey was conducted in May/June 2024 (see Methods) with n = 2145.

SI Table 1: Control variables, treatment variables and political orientation

Variable	N	Mean	SD	Min	Max
Potential in circle 1	2145	0.2424	0.4286	0	1
Potential in circle 2	2145	0.1441	0.3512	0	1
Potential in circle 3	2145	0.1399	0.3469	0	1
Age	2145	48.97	15.38	18	78
Gender	2145				
... Female	1055	49.18%			
... Male	1090	50.82%			
Environmental score	2145	5.256	1.122	1	7
Familiarity with agrivoltaics	2145	3.449	1.725	1	7
Prior agrivoltaics preference	2145	5.554	1.422	1	7
Degree of urbanization	2145	1.399	0.6912	1	3
Treatment	2145	0.6704	0.4702	0	1
Political orientation	2145	5.055	2.138	1	10

SI Table 2: Comprehension check and dependent variables 1 and 2

Variable	N	Mean	SD	Min	Max
Knowledge of potential in circle 1	2145	0.1184	0.3232	0	1
Knowledge of potential in circle 2	2145	0.1963	0.3973	0	1
Knowledge of potential in circle 3	2145	0.3939	0.4887	0	1
Personal advantage from agrivoltaics	2145	5.046	1.391	1	7
Advantage for Switzerland	2145	5.174	1.345	1	7

SI Table 3: Dependent variables 3 to 7

Variable	N	Mean	SD	Min	Max
Expansion <1ha	2145	5.144	1.413	1	7
Expansion <5ha	2145	4.679	1.581	1	7
Expansion <10ha	2145	4.138	1.743	1	7
Expansion in Switzerland	2145	5.192	1.336	1	7
Expansion in neighbourhood	2145	4.841	1.468	1	7

SI Table 4: Dependent variables 8 to 12

Variable	N	Mean	SD	Min	Max
General policy support	2145	4.813	1.656	1	7
Simplified approval procedures	2145	4.608	1.594	1	7
Advisory services for farmers	2145	5.271	1.482	1	7
Increased one-off payments	2145	4.445	1.472	1	7
Financial support for large projects	2145	4.6	1.546	1	7

SI Section C - Regression results and additional figures

Section C1 – By political orientation

SI Table 5

	CC1, Left	CC1, Centre	CC1, Right	CC2, Left	CC2, Centre	CC2, Right	CC3, Left	CC3, Centre	CC3, Right
Intercept	0.047	0.044	−0.065	0.260*	−0.145	0.170	0.488**	0.085	0.294*
	(0.094)	(0.082)	(0.095)	(0.122)	(0.100)	(0.122)	(0.150)	(0.117)	(0.138)
Treatment	0.027	0.006	−0.012	−0.101***	−0.027	−0.119**	−0.275***	−0.191***	−0.204***
	(0.022)	(0.023)	(0.032)	(0.028)	(0.030)	(0.040)	(0.035)	(0.036)	(0.044)
Realistic agrivoltaics potential									
Potential in circle 1	0.376***	0.177***	0.203***	0.142**	0.186***	0.100*	−0.008	0.023	0.009
	(0.046)	(0.036)	(0.042)	(0.045)	(0.042)	(0.043)	(0.048)	(0.045)	(0.050)
Potential in circle 2	0.047	0.031	0.061	0.383***	0.139*	0.165*	0.121	0.127*	0.104
	(0.069)	(0.048)	(0.069)	(0.073)	(0.058)	(0.082)	(0.076)	(0.063)	(0.096)
Potential in circle 3	0.064	0.001	−0.060	−0.035	0.018	0.015	0.312***	0.255***	0.135
	(0.060)	(0.045)	(0.059)	(0.059)	(0.056)	(0.078)	(0.068)	(0.062)	(0.094)
Controls									
Age	−0.002**	−0.002**	0.000	−0.001$^{\cdot}$	−0.003**	−0.002$^{\cdot}$	−0.001	−0.002$^{\cdot}$	−0.002$^{\cdot}$
	(0.001)	(0.001)	(0.001)	(0.001)	(0.001)	(0.001)	(0.001)	(0.001)	(0.001)
Gender: Male	0.006	0.017	0.020	0.023	0.087**	0.002	0.062$^{\cdot}$	0.118***	−0.050
	(0.021)	(0.021)	(0.029)	(0.025)	(0.027)	(0.036)	(0.032)	(0.032)	(0.044)
Environmental score	−0.015	0.002	−0.005	−0.030*	0.020	−0.012	−0.017	0.037*	0.012
	(0.013)	(0.011)	(0.012)	(0.015)	(0.014)	(0.015)	(0.019)	(0.016)	(0.018)
Familiarity with agrivoltaics	0.011$^{\cdot}$	0.001	0.016$^{\cdot}$	0.009	0.008	0.023*	0.018*	0.006	0.020
	(0.006)	(0.007)	(0.009)	(0.007)	(0.009)	(0.011)	(0.009)	(0.010)	(0.013)
Prior agrivoltaics preference	0.023**	0.014$^{\cdot}$	0.020$^{\cdot}$	0.018$^{\cdot}$	0.044***	0.033**	0.016	0.053***	0.051***
	(0.008)	(0.008)	(0.011)	(0.010)	(0.010)	(0.012)	(0.013)	(0.011)	(0.013)
Degree of urbanization	−0.029	0.014	−0.021	0.014	0.010	−0.032	−0.014	−0.037	−0.044
	(0.023)	(0.017)	(0.020)	(0.023)	(0.022)	(0.023)	(0.028)	(0.025)	(0.027)
Observations	818	807	520	818	807	520	818	807	520
R^2	0.248	0.092	0.094	0.172	0.140	0.099	0.144	0.158	0.117
Adj. R^2	0.239	0.081	0.076	0.161	0.129	0.081	0.134	0.148	0.099
RMSE	0.285	0.299	0.322	0.350	0.381	0.387	0.451	0.457	0.459

Table 5: CC1: Knowledge of potential in circle 1 (0m-500m, binary), CC2: Knowledge of potential in circle 2 (500m-1500m, binary), CC3: Knowledge of potential in circle 3 (1500m-4500m, binary). Statistical significance was derived from linear least squares regression and two-sided tests. Respective standard errors are reported in brackets and significant effects are indicated with: *** for $p < 0.001$, ** for $p < 0.01$, * for $p < 0.05$ and $\cdot$ for $p < 0.1$.

SI Table 6

	CC1, low, Left	CC1, low, Centre	CC1, low, Right	CC1, high, Left	CC1, high, Centre	CC1, high, Right
Intercept	0.075	$0.121^{\cdot}$	−0.049	0.355	0.007	0.046
	(0.082)	(0.073)	(0.091)	(0.298)	(0.249)	(0.222)
Treatment	-0.043^{*}	$-0.039^{\cdot}$	$-0.058^{\cdot}$	0.302^{***}	0.150^{*}	0.115
	(0.019)	(0.022)	(0.032)	(0.075)	(0.062)	(0.079)
Controls						
Age	0.000	−0.001	0.001	-0.009^{***}	-0.006^{**}	−0.003
	(0.000)	(0.001)	(0.001)	(0.002)	(0.002)	(0.002)
Gender: Male	0.006	0.010	$0.047^{\cdot}$	−0.015	0.006	−0.009
	(0.017)	(0.020)	(0.025)	(0.072)	(0.057)	(0.071)
Environmental score	−0.017	−0.004	−0.004	0.008	0.035	0.009
	(0.012)	(0.010)	(0.012)	(0.036)	(0.033)	(0.032)
Familiarity with agrivoltaics	0.008	0.005	0.018^{*}	0.023	−0.016	0.014
	(0.005)	(0.007)	(0.009)	(0.022)	(0.017)	(0.021)
Prior agrivoltaics preference	0.004	0.003	0.002	0.068^{**}	$0.039^{\cdot}$	0.059^{*}
	(0.006)	(0.008)	(0.011)	(0.025)	(0.021)	(0.026)
Degree of urbanization	0.035	−0.004	0.000	-0.100^{*}	0.055	−0.056
	(0.022)	(0.015)	(0.019)	(0.048)	(0.039)	(0.043)
Observations	662	597	366	156	210	154
R^2	0.027	0.013	0.048	0.272	0.093	0.083
Adj. R^2	0.016	0.001	0.030	0.237	0.061	0.039
RMSE	0.210	0.235	0.253	0.435	0.422	0.435

Table 6: CC1: Knowledge of potential in circle 1 (0m-500m, binary).Statistical significance was derived from linear least squares regression and two-sided tests. Respective standard errors are reported in brackets and significant effects are indicated with: *** for $p < 0.001$, ** for $p < 0.01$, * for $p < 0.05$ and $\cdot$ for $p < 0.1$.

SI Table 7

	CC2, low, Left	CC2, low, Centre	CC2, low, Right	CC2, high, Left	CC2, high, Centre	CC2, high, Right
Intercept	0.365^{**}	-0.082	0.171	-0.220	-0.292	0.377
	(0.121)	(0.103)	(0.128)	(0.448)	(0.313)	(0.371)
Treatment	-0.129^{***}	-0.038	-0.123^{**}	$0.205^{\cdot}$	0.095	-0.065
	(0.029)	(0.032)	(0.042)	(0.110)	(0.080)	(0.116)
Controls						
Age	-0.001	$-0.002^{\cdot}$	-0.001	$-0.006^{\cdot}$	-0.007^{**}	-0.009^{*}
	(0.001)	(0.001)	(0.001)	(0.003)	(0.002)	(0.004)
Gender: Male	0.010	$0.047^{\cdot}$	0.018	0.118	0.234^{**}	-0.060
	(0.025)	(0.028)	(0.037)	(0.106)	(0.075)	(0.101)
Environmental score	-0.040^{**}	0.007	-0.012	0.047	0.082^{*}	0.037
	(0.015)	(0.015)	(0.015)	(0.063)	(0.037)	(0.047)
Familiarity with agrivoltaics	0.010	0.019^{*}	0.032^{**}	-0.022	-0.022	-0.008
	(0.008)	(0.010)	(0.012)	(0.033)	(0.024)	(0.029)
Prior agrivoltaics preference	0.009	0.035^{***}	0.015	0.096^{**}	0.094^{***}	0.101^{**}
	(0.010)	(0.010)	(0.012)	(0.033)	(0.027)	(0.035)
Degree of urbanization	0.023	0.033	-0.015	0.090	0.005	-0.074
	(0.022)	(0.023)	(0.023)	(0.078)	(0.055)	(0.073)
Observations	733	663	440	85	144	80
R^2	0.049	0.042	0.052	0.172	0.204	0.246
Adj. R^2	0.040	0.032	0.037	0.097	0.163	0.173
RMSE	0.331	0.365	0.369	0.472	0.454	0.446

Table 7: CC2: Knowledge of potential in circle 2 (500m-1500m, binary). Statistical significance was derived from linear least squares regression and two-sided tests. Respective standard errors are reported in brackets and significant effects are indicated with: *** for $p < 0.001$, ** for $p < 0.01$, * for $p < 0.05$ and $\cdot$ for $p < 0.1$.

SI Table 8

	CC3, low, Left	CC3, low, Centre	CC3, low, Right	CC3, high, Left	CC3, high, Centre	CC3, high, Right
Intercept	0.605^{***}	0.020	$0.278^{\cdot}$	0.129	0.822^{**}	0.533
	(0.160)	(0.126)	(0.146)	(0.381)	(0.299)	(0.395)
Treatment	-0.318^{***}	-0.211^{***}	-0.238^{***}	0.059	−0.064	0.003
	(0.037)	(0.040)	(0.048)	(0.109)	(0.077)	(0.120)
Controls						
Age	−0.001	−0.001	−0.001	−0.001	-0.005^{*}	$-0.008^{\cdot}$
	(0.001)	(0.001)	(0.001)	(0.003)	(0.002)	(0.004)
Gender: Male	0.048	0.105^{**}	−0.061	0.162	0.256^{***}	0.013
	(0.034)	(0.036)	(0.047)	(0.102)	(0.076)	(0.118)
Environmental score	−0.029	0.038^{*}	0.009	0.049	0.027	0.047
	(0.020)	(0.017)	(0.019)	(0.056)	(0.040)	(0.056)
Familiarity with agrivoltaics	$0.016^{\cdot}$	0.014	0.031^{*}	0.011	$-0.048^{\cdot}$	−0.030
	(0.010)	(0.011)	(0.014)	(0.034)	(0.025)	(0.036)
Prior agrivoltaics preference	0.013	0.049^{***}	0.047^{**}	0.034	0.065^{*}	$0.073^{\cdot}$
	(0.013)	(0.012)	(0.014)	(0.041)	(0.029)	(0.041)
Degree of urbanization	−0.011	−0.004	−0.045	0.012	-0.158^{**}	−0.024
	(0.030)	(0.027)	(0.028)	(0.073)	(0.053)	(0.076)
Observations	725	677	443	93	130	77
R^2	0.113	0.091	0.106	0.068	0.229	0.171
Adj. R^2	0.104	0.081	0.091	−0.009	0.185	0.087
RMSE	0.446	0.464	0.452	0.463	0.406	0.479

Table 8: CC1: Knowledge of potential in circle 3 (1500m-4500m, binary). Statistical significance was derived from linear least squares regression and two-sided tests. Respective standard errors are reported in brackets and significant effects are indicated with: *** for $p < 0.001$, ** for $p < 0.01$, * for $p < 0.05$ and · for $p < 0.1$.

SI Table 9

	DV1, Left	DV1, Centre	DV1, Right	DV2, Left	DV2, Centre	DV2, Right
Intercept	1.328***	1.370***	0.943**	1.349***	1.368***	1.102***
	(0.321)	(0.279)	(0.310)	(0.307)	(0.270)	(0.326)
Treatment	−0.142$^{\cdot}$	−0.076	−0.063	−0.051	−0.059	−0.077
	(0.074)	(0.075)	(0.100)	(0.067)	(0.077)	(0.091)
Realistic agrivoltaics potential						
Potential in circle 1	−0.110	0.136	−0.206$^{\cdot}$	0.008	0.112	−0.116
	(0.110)	(0.092)	(0.119)	(0.096)	(0.086)	(0.114)
Potential in circle 2	−0.236	−0.049	0.060	−0.175	−0.097	−0.112
	(0.171)	(0.139)	(0.163)	(0.169)	(0.121)	(0.163)
Potential in circle 3	0.225	−0.041	0.024	0.249$^{\cdot}$	−0.022	0.038
	(0.140)	(0.139)	(0.158)	(0.138)	(0.113)	(0.161)
Controls						
Age	−0.003	−0.004	0.003	−0.003	−0.001	0.003
	(0.002)	(0.002)	(0.003)	(0.002)	(0.002)	(0.003)
Gender: Male	−0.012	−0.009	0.079	−0.017	0.004	0.010
	(0.079)	(0.073)	(0.095)	(0.068)	(0.072)	(0.088)
Environmental score	0.081$^{\cdot}$	0.147***	0.163***	0.067$^{\cdot}$	0.165***	0.142**
	(0.042)	(0.040)	(0.048)	(0.037)	(0.040)	(0.044)
Familiarity with agrivoltaics	0.060*	0.033	0.019	0.044*	0.037	−0.017
	(0.024)	(0.024)	(0.027)	(0.020)	(0.024)	(0.026)
Prior agrivoltaics preference	0.628***	0.544***	0.598***	0.651***	0.535***	0.620***
	(0.035)	(0.032)	(0.038)	(0.033)	(0.031)	(0.036)
Degree of urbanization	−0.084	−0.003	−0.188**	−0.030	−0.057	−0.109$^{\cdot}$
	(0.074)	(0.056)	(0.068)	(0.061)	(0.059)	(0.064)
Observations	818	807	520	818	807	520
R^2	0.382	0.398	0.507	0.453	0.402	0.539
Adj. R^2	0.374	0.390	0.498	0.446	0.395	0.530
RMSE	1.074	1.020	1.044	0.937	1.011	0.981

Table 9: DV1: Personal advantage from agrivoltaics (1-7 Likert scale), DV2: Advantage for Switzerland (1-7 Likert scale). Statistical significance was derived from linear least squares regression and two-sided tests. Respective standard errors are reported in brackets and significant effects are indicated with: *** for $p < 0.001$, ** for $p < 0.01$, * for $p < 0.05$ and $\cdot$ for $p < 0.1$.

SI Table 10

	DV3, Left	DV3, Centre	DV3, Right	DV4, Left	DV4, Centre	DV4, Right	DV5, Left	DV5, Centre	DV5, Right	DV6, Left	DV6, Centre	DV6, Right	DV7, Left	DV7, Centre	DV7, Right
Intercept	1.634***	1.371***	0.452	0.982**	0.557·	−0.171	0.260	0.202	−0.298	1.135***	0.722**	0.246	0.718*	0.419	−0.093
	(0.333)	(0.279)	(0.355)	(0.361)	(0.301)	(0.366)	(0.425)	(0.358)	(0.407)	(0.282)	(0.234)	(0.309)	(0.335)	(0.272)	(0.317)
Treatment	−0.073	−0.112	0.022	−0.110	−0.144·	−0.141	−0.204*	−0.088	−0.121	−0.132*	−0.136*	−0.063	−0.053	−0.151·	−0.135
	(0.068)	(0.079)	(0.107)	(0.078)	(0.087)	(0.117)	(0.098)	(0.105)	(0.141)	(0.061)	(0.068)	(0.092)	(0.072)	(0.078)	(0.105)
Realistic agrivoltaics potential															
Potential in circle 1	0.164	0.124	−0.315*	0.080	0.062	−0.381**	−0.178	0.138	−0.453**	−0.090	0.034	−0.281**	−0.158	−0.001	−0.479***
	(0.102)	(0.093)	(0.142)	(0.118)	(0.118)	(0.144)	(0.136)	(0.137)	(0.162)	(0.090)	(0.078)	(0.109)	(0.114)	(0.101)	(0.125)
Potential in circle 2	−0.207	0.065	0.305	−0.165	0.152	0.472*	−0.239	0.227	0.334	0.051	−0.028	0.018	−0.059	0.073	0.593**
	(0.158)	(0.120)	(0.222)	(0.171)	(0.150)	(0.220)	(0.223)	(0.178)	(0.266)	(0.122)	(0.104)	(0.144)	(0.187)	(0.150)	(0.195)
Potential in circle 3	0.013	−0.059	−0.225	0.060	−0.265·	−0.546*	0.146	−0.495**	−0.464·	−0.011	−0.064	0.102	0.089	−0.094	−0.438*
	(0.134)	(0.121)	(0.210)	(0.151)	(0.154)	(0.221)	(0.214)	(0.173)	(0.269)	(0.107)	(0.111)	(0.142)	(0.162)	(0.140)	(0.192)
Controls															
Age	−0.002	0.000	0.008*	−0.005*	−0.002	0.004	−0.008*	−0.006*	0.002	0.002	0.004	0.010**	−0.002	0.003	0.009**
	(0.002)	(0.002)	(0.004)	(0.002)	(0.003)	(0.004)	(0.003)	(0.003)	(0.005)	(0.002)	(0.002)	(0.003)	(0.002)	(0.002)	(0.004)
Gender: Male	−0.036	0.109	0.044	0.166*	0.441***	0.314*	0.288**	0.618***	0.446**	0.029	0.097	0.027	0.215**	0.283***	0.101
	(0.067)	(0.074)	(0.110)	(0.077)	(0.084)	(0.126)	(0.097)	(0.099)	(0.145)	(0.062)	(0.065)	(0.091)	(0.072)	(0.075)	(0.107)
Environmental score	0.118**	0.166***	0.185***	0.101*	0.159***	0.166**	0.130*	0.159**	0.126*	0.069*	0.208***	0.177***	0.119**	0.222***	0.210***
	(0.038)	(0.039)	(0.047)	(0.042)	(0.043)	(0.054)	(0.053)	(0.051)	(0.059)	(0.034)	(0.034)	(0.041)	(0.043)	(0.040)	(0.046)
Familiarity with agrivoltaics	0.027	0.058*	−0.031	0.026	0.063*	−0.011	0.038	0.074*	−0.019	0.050**	0.062**	0.032	0.064**	0.067**	0.011
	(0.021)	(0.024)	(0.033)	(0.024)	(0.027)	(0.038)	(0.029)	(0.032)	(0.043)	(0.019)	(0.020)	(0.026)	(0.024)	(0.024)	(0.031)
Prior agrivoltaics preference	0.581***	0.506***	0.669***	0.673***	0.567***	0.692***	0.692***	0.546***	0.630***	0.656***	0.555***	0.641***	0.644***	0.547***	0.636***
	(0.035)	(0.033)	(0.035)	(0.036)	(0.032)	(0.039)	(0.039)	(0.035)	(0.043)	(0.031)	(0.031)	(0.034)	(0.034)	(0.031)	(0.036)
Degree of urbanization	−0.114·	−0.077	−0.257**	−0.182*	−0.112·	−0.127	−0.168·	−0.112	0.006	−0.009	−0.029	−0.120·	−0.049	−0.139*	−0.151*
	(0.058)	(0.060)	(0.078)	(0.077)	(0.067)	(0.082)	(0.089)	(0.076)	(0.089)	(0.049)	(0.055)	(0.064)	(0.056)	(0.068)	(0.073)
Observations	818	807	520	818	807	520	818	807	520	818	807	520	818	807	520
R^2	0.405	0.375	0.522	0.415	0.381	0.470	0.346	0.311	0.347	0.502	0.496	0.572	0.434	0.428	0.522
Adj. R^2	0.398	0.368	0.512	0.408	0.374	0.460	0.338	0.302	0.334	0.496	0.490	0.563	0.427	0.421	0.512
RMSE	0.943	1.034	1.164	1.080	1.169	1.302	1.344	1.382	1.511	0.855	0.897	0.981	0.999	1.048	1.131

Table 10: DV3: Expansion <1ha, DV4: Expansion <5ha, DV5: Expansion <10ha, DV6: Expansion in Switzerland, DV7: Expanion in neighbourhood. Statistical significance was derived from linear least squares regression and two-sided tests. Respective standard errors are reported in brackets and significant effects are indicated with: *** for $p < 0.001$, ** for $p < 0.01$, * for $p < 0.05$ and · for $p < 0.1$.

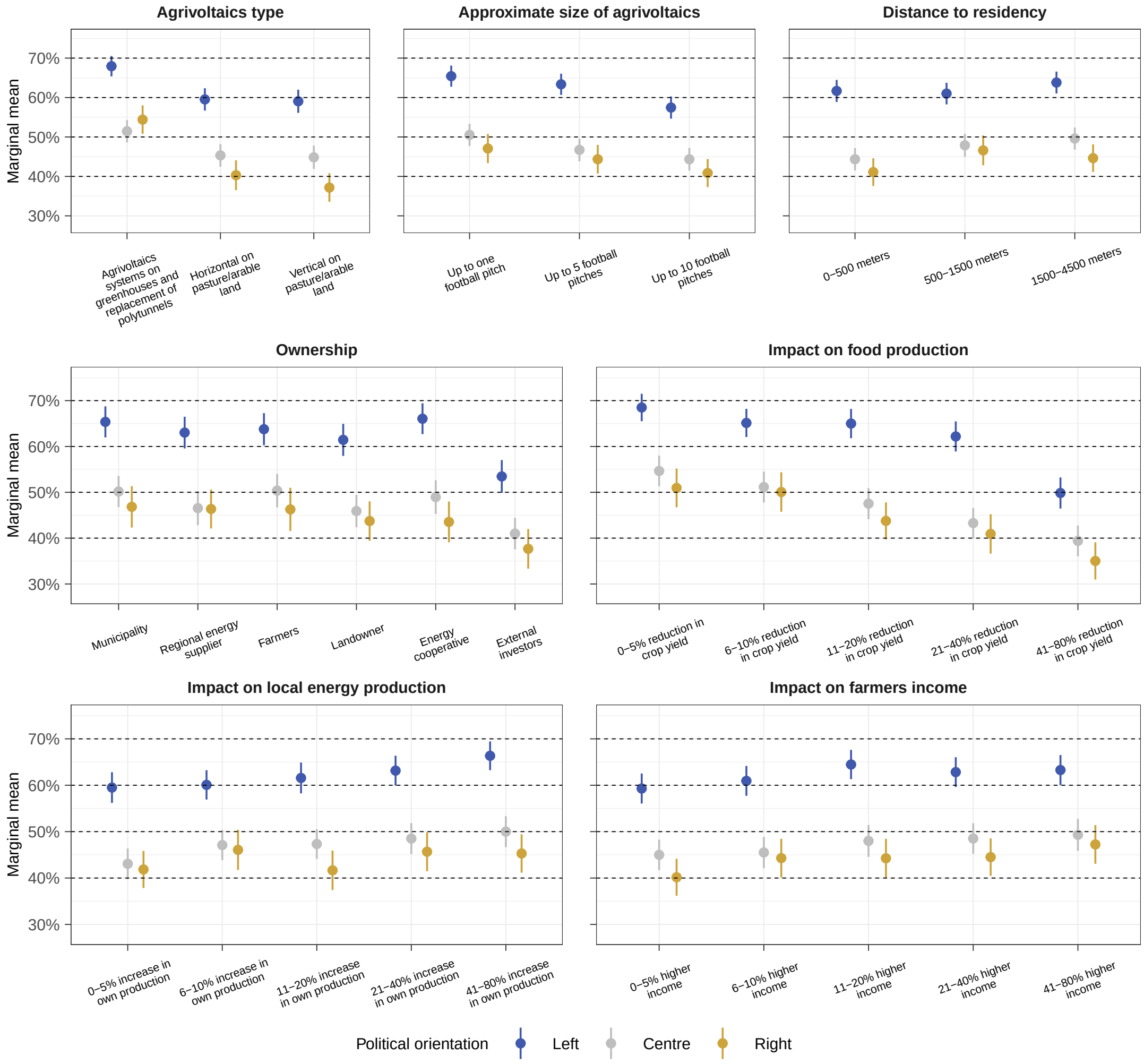

Number of observations: 17160 Number of respondents: 2145

SI Figure 7: The figure displays the marginal means for clear support — defined as a support rating greater than 5 on a 7-point Likert scale — for differently designed agrivoltaics projects among left-leaning (blue, n = 818), centric (grey, n = 807), and right-leaning (yellow, n = 520) voters in the overall sample (n = 2145). Here, the marginal means represent the predicted probability that a respondent from a particular subgroup expresses clear support for an agrivoltaics profile containing a given attribute level. Data points indicate the estimated marginal means for clear support (ranging between 0%-100%) for an agrivoltaics project with the respective design attribute. Vertical lines represent cluster-robust 95% confidence intervals derived from linear least squares regression and two-sided tests. Detailed results can be found in SI Table 11

SI Table 11: The table displays the marginal means displayed in SI Figure 7. The total N is 17'160 observations, i.e., 4 choice rounds between each 2 agrivoltaics design options for 2145 respondents (see further details in Methods)

Feature	Level	Political orientation	Marginal mean	SE	Z-Value	p-Value	Significance
Agrivoltaics type	On greenhouses/replace polytunnels	Left	0.679	0.013	51.624	<0.001	***
		Centre	0.515	0.014	35.580	<0.001	***
		Right	0.544	0.018	29.539	<0.001	***
	Horizontal on pasture/arable land	Left	0.595	0.014	41.121	<0.001	***
		Centre	0.453	0.015	30.985	<0.001	***
		Right	0.403	0.019	20.898	<0.001	***
	Vertical on pasture/arable land	Left	0.591	0.015	39.393	<0.001	***
		Centre	0.449	0.015	29.769	<0.001	***
		Right	0.372	0.019	20.088	<0.001	***
Approximate size of agrivoltaics	Up to one football pitch	Left	0.654	0.014	47.789	<0.001	***
		Centre	0.505	0.014	35.261	<0.001	***
		Right	0.471	0.019	24.887	<0.001	***
	Up to 5 football pitches	Left	0.634	0.014	46.233	<0.001	***
		Centre	0.467	0.015	31.703	<0.001	***
		Right	0.444	0.019	23.881	<0.001	***
	Up to 10 football pitches	Left	0.575	0.014	40.077	<0.001	***
		Centre	0.443	0.015	30.099	<0.001	***
		Right	0.409	0.018	22.498	<0.001	***
Distance to residency	0-500 meters	Left	0.617	0.014	43.309	<0.001	***

		Centre	0.444	0.014	30.662	<0.001	***
		Right	0.411	0.018	22.937	<0.001	***
	500-1500 meters	Left	0.610	0.014	43.693	<0.001	***
		Centre	0.479	0.015	32.238	<0.001	***
		Right	0.466	0.019	24.209	<0.001	***
	1500-4500 meters	Left	0.638	0.014	45.104	<0.001	***
		Centre	0.496	0.014	34.842	<0.001	***
		Right	0.446	0.018	25.072	<0.001	***
Ownership	Municipality	Left	0.654	0.017	37.769	<0.001	***
		Centre	0.502	0.017	28.937	<0.001	***
		Right	0.468	0.023	20.315	<0.001	***
	Regional energy supplier	Left	0.630	0.018	35.729	<0.001	***
		Centre	0.465	0.019	24.722	<0.001	***
		Right	0.464	0.022	21.446	<0.001	***
	Farmers	Left	0.638	0.018	35.731	<0.001	***
		Centre	0.504	0.019	27.123	<0.001	***
		Right	0.463	0.024	19.240	<0.001	***
	Landowner	Left	0.614	0.018	34.449	<0.001	***
		Centre	0.459	0.018	25.477	<0.001	***
		Right	0.437	0.022	19.922	<0.001	***
	Energy cooperative	Left	0.661	0.017	38.533	<0.001	***
		Centre	0.490	0.019	26.058	<0.001	***
		Right	0.435	0.023	19.162	<0.001	***
	External investors	Left	0.535	0.018	29.333	<0.001	***
		Centre	0.410	0.018	23.339	<0.001	***

		Right	0.377	0.022	17.110	<0.001	***
Impact on food production	0-5% reduction in crop yield	Left	0.685	0.015	44.831	<0.001	***
		Centre	0.547	0.017	31.973	<0.001	***
		Right	0.510	0.022	23.681	<0.001	***
	6-10% reduction in crop yield	Left	0.651	0.016	41.517	<0.001	***
		Centre	0.511	0.017	29.779	<0.001	***
		Right	0.501	0.022	22.776	<0.001	***
	11-20% reduction in crop yield	Left	0.650	0.016	39.953	<0.001	***
		Centre	0.475	0.017	27.520	<0.001	***
		Right	0.438	0.021	21.225	<0.001	***
	21-40% reduction in crop yield	Left	0.622	0.017	37.131	<0.001	***
		Centre	0.433	0.017	25.732	<0.001	***
		Right	0.409	0.022	18.662	<0.001	***
	41-80% reduction in crop yield	Left	0.498	0.017	28.792	<0.001	***
		Centre	0.394	0.017	23.032	<0.001	***
		Right	0.350	0.021	16.901	<0.001	***
Impact on local energy production	0-5% increase in own production	Left	0.595	0.017	35.333	<0.001	***
		Centre	0.431	0.017	25.510	<0.001	***
		Right	0.418	0.020	20.541	<0.001	***
	6-10% increase in own production	Left	0.601	0.016	37.279	<0.001	***
		Centre	0.471	0.017	28.436	<0.001	***

		Right	0.461	0.022	20.911	<0.001	***
	11-20% increase in own production	Left	0.616	0.017	36.404	<0.001	***
		Centre	0.473	0.016	28.917	<0.001	***
		Right	0.417	0.022	19.190	<0.001	***
	21-40% increase in own production	Left	0.631	0.016	38.600	<0.001	***
		Centre	0.485	0.017	28.543	<0.001	***
		Right	0.457	0.021	21.280	<0.001	***
	41-80% increase in own production	Left	0.663	0.016	41.923	<0.001	***
		Centre	0.500	0.017	29.422	<0.001	***
		Right	0.453	0.021	21.463	<0.001	***
Impact on farmers income	0-5% higher income	Left	0.593	0.017	35.926	<0.001	***
		Centre	0.450	0.017	26.918	<0.001	***
		Right	0.402	0.020	19.739	<0.001	***
	6-10% higher income	Left	0.609	0.016	37.150	<0.001	***
		Centre	0.455	0.017	26.567	<0.001	***
		Right	0.443	0.021	20.863	<0.001	***
	11-20% higher income	Left	0.645	0.016	40.094	<0.001	***
		Centre	0.480	0.017	27.446	<0.001	***
		Right	0.443	0.021	20.671	<0.001	***
	21-40% higher income	Left	0.628	0.016	38.311	<0.001	***
		Centre	0.485	0.017	28.973	<0.001	***
		Right	0.445	0.021	21.635	<0.001	***
	41-80% higher income	Left	0.633	0.016	38.405	<0.001	***

Centre	0.493	0.018	28.023	<0.001	***
Right	0.472	0.021	22.252	<0.001	***

SI Table 12: The table displays the marginal means displayed in Figure 4 in the main manuscript. The total N is 17'160 observations, i.e., 4 choice rounds between each 2 agrivoltaics design options for 2145 respondents (see further details in Methods)

Feature	Level	Group	Political orientation	Marginal mean	SE	Z-Value	p-Value	Significance
Agrivoltaics type	On greenhouses/replace polytunnels	Control	Centre	0.537	0.027	20.173	<0.001	***
			Left	0.697	0.021	32.454	<0.001	***
			Overall	0.612	0.015	40.313	<0.001	***
			Right	0.591	0.030	19.524	<0.001	***
		Treatment	Centre	0.504	0.017	29.268	<0.001	***
			Left	0.671	0.017	40.442	<0.001	***
			Overall	0.570	0.011	52.381	<0.001	***
			Right	0.520	0.023	22.599	<0.001	***
	Horizontal on pasture/arable land	Control	Centre	0.475	0.027	17.810	<0.001	***
			Left	0.602	0.025	24.132	<0.001	***
			Overall	0.521	0.016	32.274	<0.001	***
			Right	0.455	0.033	13.645	<0.001	***
		Treatment	Centre	0.443	0.017	25.382	<0.001	***
			Left	0.592	0.018	33.262	<0.001	***
			Overall	0.484	0.011	42.898	<0.001	***
			Right	0.377	0.023	16.047	<0.001	***
	Vertical on pasture/arable land	Control	Centre	0.455	0.026	17.553	<0.001	***
			Left	0.577	0.025	22.743	<0.001	***
			Overall	0.490	0.016	30.290	<0.001	***
			Right	0.404	0.034	11.915	<0.001	***
		Treatment	Centre	0.445	0.019	24.059	<0.001	***
			Left	0.597	0.019	32.186	<0.001	***

			Overall	0.481	0.012	41.394	<0.001	***
			Right	0.356	0.022	16.248	<0.001	***
Approximate size of agrivoltaics	Up to one football pitch	Control	Centre	0.518	0.025	20.758	<0.001	***
			Left	0.639	0.024	26.474	<0.001	***
			Overall	0.569	0.015	37.103	<0.001	***
			Right	0.530	0.031	17.038	<0.001	***
		Treatment	Centre	0.500	0.017	28.577	<0.001	***
			Left	0.662	0.017	39.831	<0.001	***
			Overall	0.546	0.011	49.135	<0.001	***
			Right	0.441	0.023	18.883	<0.001	***
	Up to 5 football pitches	Control	Centre	0.498	0.026	19.189	<0.001	***
			Left	0.651	0.023	28.318	<0.001	***
			Overall	0.557	0.016	35.384	<0.001	***
			Right	0.490	0.034	14.573	<0.001	***
		Treatment	Centre	0.452	0.018	25.318	<0.001	***
			Left	0.625	0.017	36.674	<0.001	***
			Overall	0.512	0.011	46.308	<0.001	***
			Right	0.420	0.022	18.946	<0.001	***
	Up to 10 football pitches	Control	Centre	0.452	0.026	17.171	<0.001	***
			Left	0.583	0.024	24.114	<0.001	***
			Overall	0.498	0.016	31.977	<0.001	***
			Right	0.439	0.030	14.585	<0.001	***
		Treatment	Centre	0.439	0.018	24.700	<0.001	***
			Left	0.570	0.018	32.032	<0.001	***
			Overall	0.477	0.011	42.425	<0.001	***

			Right	0.392	0.023	17.270	<0.001	***
Distance to residency	0-500 meters	Control	Centre	0.479	0.025	18.918	<0.001	***
			Left	0.610	0.025	24.161	<0.001	***
			Overall	0.524	0.016	33.433	<0.001	***
			Right	0.461	0.030	15.192	<0.001	***
		Treatment	Centre	0.427	0.018	24.280	<0.001	***
			Left	0.620	0.017	35.947	<0.001	***
			Overall	0.489	0.011	43.654	<0.001	***
			Right	0.386	0.022	17.397	<0.001	***
	500-1500 meters	Control	Centre	0.482	0.027	18.081	<0.001	***
			Left	0.608	0.024	25.201	<0.001	***
			Overall	0.546	0.016	34.661	<0.001	***
			Right	0.532	0.032	16.744	<0.001	***
		Treatment	Centre	0.477	0.018	26.676	<0.001	***
			Left	0.611	0.017	35.661	<0.001	***
			Overall	0.519	0.011	46.269	<0.001	***
			Right	0.431	0.024	18.085	<0.001	***
	1500-4500 meters	Control	Centre	0.506	0.026	19.431	<0.001	***
			Left	0.657	0.024	27.903	<0.001	***
			Overall	0.555	0.016	35.557	<0.001	***
			Right	0.464	0.031	14.956	<0.001	***
		Treatment	Centre	0.491	0.017	28.955	<0.001	***
			Left	0.628	0.018	35.498	<0.001	***
			Overall	0.528	0.011	48.611	<0.001	***
			Right	0.437	0.022	20.077	<0.001	***

Ownership	Municipality	Control	Centre	0.480	0.029	16.845	<0.001	***
			Left	0.615	0.030	20.768	<0.001	***
			Overall	0.535	0.019	28.794	<0.001	***
			Right	0.502	0.041	12.390	<0.001	***
		Treatment	Centre	0.512	0.022	23.625	<0.001	***
			Left	0.673	0.021	31.800	<0.001	***
			Overall	0.556	0.014	40.713	<0.001	***
			Right	0.451	0.028	16.061	<0.001	***
	Regional energy supplier	Control	Centre	0.489	0.034	14.457	<0.001	***
			Left	0.658	0.028	23.243	<0.001	***
			Overall	0.557	0.019	29.221	<0.001	***
			Right	0.486	0.036	13.469	<0.001	***
		Treatment	Centre	0.455	0.023	20.128	<0.001	***
			Left	0.615	0.022	27.549	<0.001	***
			Overall	0.514	0.014	37.025	<0.001	***
			Right	0.452	0.027	16.784	<0.001	***
	Farmers	Control	Centre	0.550	0.033	16.518	<0.001	***
			Left	0.640	0.030	21.401	<0.001	***
			Overall	0.580	0.020	28.745	<0.001	***
			Right	0.533	0.045	11.932	<0.001	***
		Treatment	Centre	0.483	0.022	21.645	<0.001	***
			Left	0.637	0.022	28.729	<0.001	***
			Overall	0.529	0.014	37.733	<0.001	***
			Right	0.430	0.028	15.144	<0.001	***
	Landowner	Control	Centre	0.480	0.032	14.901	<0.001	***

			Left	0.636	0.031	20.824	<0.001	***
			Overall	0.551	0.019	28.616	<0.001	***
			Right	0.524	0.036	14.556	<0.001	***
		Treatment	Centre	0.449	0.022	20.680	<0.001	***
			Left	0.603	0.022	27.488	<0.001	***
			Overall	0.492	0.014	35.677	<0.001	***
			Right	0.387	0.027	14.307	<0.001	***
	Energy cooperative	Control	Centre	0.510	0.034	14.799	<0.001	***
			Left	0.679	0.028	24.394	<0.001	***
			Overall	0.562	0.019	29.177	<0.001	***
			Right	0.450	0.036	12.443	<0.001	***
		Treatment	Centre	0.480	0.022	21.446	<0.001	***
			Left	0.651	0.022	30.033	<0.001	***
			Overall	0.533	0.014	37.754	<0.001	***
			Right	0.427	0.029	14.715	<0.001	***
	External investors	Control	Centre	0.430	0.031	13.909	<0.001	***
			Left	0.523	0.033	15.747	<0.001	***
			Overall	0.464	0.020	23.501	<0.001	***
			Right	0.414	0.037	11.056	<0.001	***
		Treatment	Centre	0.400	0.021	18.724	<0.001	***
			Left	0.540	0.022	24.859	<0.001	***
			Overall	0.445	0.014	32.684	<0.001	***
			Right	0.360	0.027	13.303	<0.001	***
Impact on food production	0-5% reduction in crop yield	Control	Centre	0.538	0.031	17.428	<0.001	***
			Left	0.674	0.026	25.953	<0.001	***

		Overall	0.588	0.018	33.321	<0.001	***
		Right	0.536	0.035	15.512	<0.001	***
	Treatment	Centre	0.551	0.021	26.806	<0.001	***
		Left	0.691	0.019	36.517	<0.001	***
		Overall	0.590	0.013	46.301	<0.001	***
		Right	0.495	0.027	18.082	<0.001	***
6-10% reduction in crop yield	Control	Centre	0.495	0.030	16.677	<0.001	***
		Left	0.652	0.028	22.910	<0.001	***
		Overall	0.576	0.018	31.584	<0.001	***
		Right	0.586	0.036	16.090	<0.001	***
	Treatment	Centre	0.520	0.021	24.691	<0.001	***
		Left	0.651	0.019	34.612	<0.001	***
		Overall	0.557	0.013	43.775	<0.001	***
		Right	0.457	0.027	17.051	<0.001	***
11-20% reduction in crop yield	Control	Centre	0.491	0.031	15.719	<0.001	***
		Left	0.690	0.027	25.278	<0.001	***
		Overall	0.566	0.019	30.486	<0.001	***
		Right	0.481	0.037	12.947	<0.001	***
	Treatment	Centre	0.468	0.021	22.623	<0.001	***
		Left	0.629	0.020	31.229	<0.001	***
		Overall	0.514	0.013	40.189	<0.001	***
		Right	0.415	0.024	16.983	<0.001	***
21-40% reduction in crop yield	Control	Centre	0.480	0.030	15.887	<0.001	***
		Left	0.607	0.029	21.135	<0.001	***
		Overall	0.533	0.018	28.936	<0.001	***

			Right	0.476	0.037	12.713	<0.001	***
		Treatment	Centre	0.412	0.020	20.419	<0.001	***
			Left	0.630	0.021	30.591	<0.001	***
			Overall	0.487	0.013	36.771	<0.001	***
			Right	0.379	0.027	14.144	<0.001	***
	41-80% reduction in crop yield	Control	Centre	0.440	0.031	14.085	<0.001	***
			Left	0.508	0.028	18.172	<0.001	***
			Overall	0.445	0.018	24.269	<0.001	***
			Right	0.352	0.037	9.582	<0.001	***
		Treatment	Centre	0.372	0.020	18.341	<0.001	***
			Left	0.494	0.022	22.457	<0.001	***
			Overall	0.412	0.013	31.552	<0.001	***
			Right	0.349	0.025	13.904	<0.001	***
Impact on local energy production	0-5% increase in own production	Control	Centre	0.467	0.030	15.571	<0.001	***
			Left	0.602	0.030	20.343	<0.001	***
			Overall	0.521	0.018	28.681	<0.001	***
			Right	0.471	0.034	13.989	<0.001	***
		Treatment	Centre	0.412	0.020	20.290	<0.001	***
			Left	0.591	0.020	28.902	<0.001	***
			Overall	0.475	0.013	36.842	<0.001	***
			Right	0.392	0.025	15.409	<0.001	***
	6-10% increase in own production	Control	Centre	0.495	0.031	16.056	<0.001	***
			Left	0.583	0.027	21.581	<0.001	***
			Overall	0.540	0.018	30.395	<0.001	***
			Right	0.535	0.036	14.937	<0.001	***

	Treatment	Centre	0.461	0.020	23.446	<0.001	***
		Left	0.610	0.020	30.400	<0.001	***
		Overall	0.507	0.013	39.790	<0.001	***
		Right	0.417	0.027	15.311	<0.001	***
11-20% increase in own production	Control	Centre	0.482	0.028	17.215	<0.001	***
		Left	0.634	0.029	22.164	<0.001	***
		Overall	0.529	0.018	29.493	<0.001	***
		Right	0.442	0.037	12.038	<0.001	***
	Treatment	Centre	0.469	0.020	23.250	<0.001	***
		Left	0.607	0.021	29.011	<0.001	***
		Overall	0.506	0.013	38.811	<0.001	***
		Right	0.404	0.027	15.044	<0.001	***
21-40% increase in own production	Control	Centre	0.480	0.030	15.755	<0.001	***
		Left	0.648	0.028	22.882	<0.001	***
		Overall	0.548	0.019	29.553	<0.001	***
		Right	0.491	0.037	13.181	<0.001	***
	Treatment	Centre	0.487	0.020	23.788	<0.001	***
		Left	0.624	0.020	31.132	<0.001	***
		Overall	0.527	0.013	41.372	<0.001	***
		Right	0.440	0.026	16.772	<0.001	***
41-80% increase in own production	Control	Centre	0.523	0.030	17.583	<0.001	***
		Left	0.662	0.027	24.146	<0.001	***
		Overall	0.571	0.018	31.316	<0.001	***
		Right	0.485	0.038	12.919	<0.001	***
	Treatment	Centre	0.489	0.021	23.669	<0.001	***

			Left	0.664	0.019	34.251	<0.001	***
			Overall	0.543	0.013	42.318	<0.001	***
			Right	0.438	0.025	17.189	<0.001	***
Impact on farmers income	0-5% higher income	Control	Centre	0.483	0.032	15.221	<0.001	***
			Left	0.586	0.030	19.642	<0.001	***
			Overall	0.517	0.019	27.749	<0.001	***
			Right	0.454	0.035	12.879	<0.001	***
		Treatment	Centre	0.436	0.020	22.242	<0.001	***
			Left	0.596	0.020	30.160	<0.001	***
			Overall	0.481	0.012	38.608	<0.001	***
			Right	0.379	0.025	15.310	<0.001	***
	6-10% higher income	Control	Centre	0.444	0.031	14.317	<0.001	***
			Left	0.590	0.028	20.875	<0.001	***
			Overall	0.512	0.018	28.116	<0.001	***
			Right	0.486	0.036	13.676	<0.001	***
		Treatment	Centre	0.460	0.021	22.418	<0.001	***
			Left	0.620	0.020	30.915	<0.001	***
			Overall	0.510	0.013	39.462	<0.001	***
			Right	0.420	0.027	15.845	<0.001	***
	11-20% higher income	Control	Centre	0.523	0.032	16.255	<0.001	***
			Left	0.676	0.028	23.959	<0.001	***
			Overall	0.581	0.019	31.295	<0.001	***
			Right	0.520	0.036	14.565	<0.001	***
		Treatment	Centre	0.461	0.021	22.147	<0.001	***
			Left	0.629	0.020	32.214	<0.001	***

			Overall	0.509	0.013	39.533	<0.001	***
			Right	0.401	0.026	15.265	<0.001	***
	21-40% higher income	Control	Centre	0.499	0.029	16.936	<0.001	***
			Left	0.641	0.028	22.945	<0.001	***
			Overall	0.544	0.018	30.180	<0.001	***
			Right	0.470	0.036	12.881	<0.001	***
		Treatment	Centre	0.478	0.020	23.500	<0.001	***
			Left	0.622	0.020	30.710	<0.001	***
			Overall	0.521	0.013	41.121	<0.001	***
			Right	0.433	0.025	17.367	<0.001	***
	41-80% higher income	Control	Centre	0.500	0.030	16.688	<0.001	***
			Left	0.639	0.029	22.128	<0.001	***
			Overall	0.554	0.018	30.142	<0.001	***
			Right	0.493	0.037	13.382	<0.001	***
		Treatment	Centre	0.490	0.022	22.562	<0.001	***
			Left	0.630	0.020	31.362	<0.001	***
			Overall	0.539	0.013	41.363	<0.001	***
			Right	0.461	0.026	17.774	<0.001	***

SI Table 13

	DV8, Left	DV8, Centre	DV8, Right	DV9, Left	DV9, Centre	DV9, Right	DV10, Left	DV10, Centre	DV10, Right	DV11, Left	DV11, Centre	DV11, Right	DV12, Left	DV12, Centre	DV12, Right
Intercept	−0.194	−0.631*	−0.494	0.590	0.422	0.134	1.665***	1.253***	1.169**	1.253***	1.441***	0.788·	0.964*	1.094***	0.460
	(0.361)	(0.303)	(0.392)	(0.391)	(0.320)	(0.441)	(0.380)	(0.297)	(0.406)	(0.378)	(0.322)	(0.426)	(0.413)	(0.310)	(0.407)
Treatment	−0.068	−0.074	−0.054	−0.113	−0.166·	−0.208	−0.013	−0.196*	−0.118	−0.082	−0.133	−0.078	−0.052	−0.233*	0.012
	(0.076)	(0.086)	(0.127)	(0.087)	(0.094)	(0.141)	(0.075)	(0.083)	(0.129)	(0.087)	(0.091)	(0.136)	(0.086)	(0.091)	(0.143)
Realistic agrivoltaics potential															
Potential in circle 1	−0.032	0.002	−0.260·	−0.121	−0.082	−0.321·	0.124	−0.027	−0.063	−0.016	−0.107	−0.130	−0.024	−0.008	0.006
	(0.111)	(0.112)	(0.149)	(0.128)	(0.122)	(0.168)	(0.104)	(0.107)	(0.148)	(0.112)	(0.116)	(0.151)	(0.127)	(0.117)	(0.162)
Potential in circle 2	−0.148	−0.136	0.344·	−0.196	−0.060	0.245	−0.132	0.064	0.145	0.081	0.129	0.228	0.159	−0.045	0.195
	(0.135)	(0.165)	(0.207)	(0.189)	(0.161)	(0.234)	(0.144)	(0.142)	(0.200)	(0.184)	(0.164)	(0.204)	(0.176)	(0.157)	(0.235)
Potential in circle 3	0.094	0.144	−0.277	0.133	−0.020	−0.306	0.098	0.044	−0.078	0.058	0.142	0.015	−0.063	0.061	−0.246
	(0.126)	(0.154)	(0.214)	(0.172)	(0.166)	(0.217)	(0.135)	(0.154)	(0.193)	(0.173)	(0.173)	(0.195)	(0.188)	(0.151)	(0.235)
Controls															
Age	0.001	0.004	0.014***	0.001	0.003	0.008	0.001	0.003	0.000	0.007**	0.003	−0.002	−0.006*	−0.003	−0.003
	(0.002)	(0.003)	(0.004)	(0.003)	(0.003)	(0.005)	(0.002)	(0.003)	(0.004)	(0.003)	(0.003)	(0.004)	(0.002)	(0.003)	(0.005)
Gender: Male	0.005	0.173*	−0.005	0.144·	0.387***	0.195	−0.084	0.077	0.010	−0.039	−0.010	0.044	−0.171*	0.019	0.034
	(0.076)	(0.086)	(0.129)	(0.087)	(0.091)	(0.141)	(0.074)	(0.083)	(0.117)	(0.086)	(0.088)	(0.122)	(0.086)	(0.090)	(0.129)
Environmental score	0.182***	0.252***	0.230***	0.068	0.140**	0.185**	0.076·	0.168***	0.133*	0.099·	0.076·	0.154**	0.175***	0.189***	0.233***
	(0.048)	(0.046)	(0.058)	(0.052)	(0.046)	(0.063)	(0.045)	(0.045)	(0.054)	(0.051)	(0.046)	(0.056)	(0.050)	(0.048)	(0.058)
Familiarity with agrivoltaics	0.058*	0.080**	0.033	0.053*	0.099***	0.043	0.039·	0.038	0.012	0.017	0.045	0.051	0.046·	0.073*	0.022
	(0.024)	(0.029)	(0.037)	(0.026)	(0.030)	(0.042)	(0.023)	(0.025)	(0.033)	(0.027)	(0.028)	(0.038)	(0.026)	(0.029)	(0.040)
Prior agrivoltaics preference	0.752***	0.650***	0.647***	0.656***	0.512***	0.571***	0.604***	0.555***	0.638***	0.482***	0.440***	0.514***	0.556***	0.442***	0.547***
	(0.038)	(0.032)	(0.042)	(0.037)	(0.035)	(0.045)	(0.040)	(0.035)	(0.043)	(0.038)	(0.037)	(0.043)	(0.038)	(0.039)	(0.044)
Degree of urbanization	−0.115·	−0.050	−0.273**	−0.082	0.056	−0.084	−0.095	−0.111·	−0.163*	−0.152*	−0.086	−0.148·	−0.017	0.059	−0.139
	(0.060)	(0.065)	(0.082)	(0.077)	(0.070)	(0.093)	(0.063)	(0.065)	(0.080)	(0.071)	(0.075)	(0.084)	(0.079)	(0.074)	(0.085)
Observations	818	807	520	818	807	520	818	807	520	818	807	520	818	807	520
R^2	0.475	0.438	0.452	0.345	0.314	0.328	0.367	0.366	0.421	0.226	0.236	0.304	0.307	0.251	0.321
Adj. R^2	0.468	0.431	0.441	0.337	0.305	0.315	0.359	0.358	0.410	0.217	0.226	0.290	0.298	0.241	0.307
RMSE	1.064	1.194	1.349	1.201	1.257	1.492	1.036	1.145	1.280	1.183	1.224	1.379	1.167	1.260	1.449

Table 13: DV8: General policy support , DV9: Simplified approval procedures, DV10: Advisory services for farmers, DV11: Increased one-off payments, DV12: Financial support for large projects. Statistical significance was derived from linear least squares regression and two-sided tests. Respective standard errors are reported in brackets and significant effects are indicated with: *** for $p < 0.001$, ** for $p < 0.01$, * for $p < 0.05$ and · for $p < 0.1$.

SI Table 14

	CC1, Left	CC1, Centre	CC1, Right	CC2, Left	CC2, Centre	CC2, Right	CC3, Left	CC3, Centre	CC3, Right
Intercept	0.101	0.069	−0.044	0.267*	−0.127	0.167	0.541***	0.105	0.334*
	(0.090)	(0.082)	(0.095)	(0.118)	(0.100)	(0.124)	(0.149)	(0.118)	(0.139)
Treatment	−0.036$^{\cdot}$	−0.036	−0.050	−0.119***	−0.061$^{\cdot}$	−0.117*	−0.339***	−0.225***	−0.264***
	(0.019)	(0.024)	(0.034)	(0.030)	(0.034)	(0.047)	(0.039)	(0.043)	(0.053)
Realistic agrivoltaics potential									
Potential in circle 1	0.101	0.044	0.090	0.149$^{\cdot}$	0.131$^{\cdot}$	0.111	−0.117	−0.039	−0.051
	(0.069)	(0.049)	(0.062)	(0.085)	(0.072)	(0.079)	(0.090)	(0.085)	(0.090)
Potential in circle 2	0.033	0.134$^{\cdot}$	0.009	0.085	0.141	0.130	0.074	0.165	0.055
	(0.099)	(0.075)	(0.102)	(0.125)	(0.090)	(0.133)	(0.119)	(0.119)	(0.154)
Potential in circle 3	0.117	−0.087	0.046	0.101	−0.042	0.039	0.130	0.164	0.052
	(0.093)	(0.063)	(0.097)	(0.117)	(0.083)	(0.127)	(0.111)	(0.115)	(0.148)
Controls									
Age	−0.002**	−0.002**	0.000	−0.001$^{\cdot}$	−0.003**	−0.002$^{\cdot}$	−0.001	−0.002$^{\cdot}$	−0.002$^{\cdot}$
	(0.001)	(0.001)	(0.001)	(0.001)	(0.001)	(0.001)	(0.001)	(0.001)	(0.001)
Gender: Male	0.004	0.017	0.017	0.021	0.086**	0.002	0.060$^{\cdot}$	0.118***	−0.051
	(0.020)	(0.021)	(0.028)	(0.025)	(0.027)	(0.036)	(0.032)	(0.032)	(0.044)
Environmental score	−0.018	0.004	−0.003	−0.030*	0.021	−0.012	−0.019	0.038*	0.012
	(0.012)	(0.011)	(0.012)	(0.015)	(0.014)	(0.015)	(0.019)	(0.016)	(0.018)
Familiarity with agrivoltaics	0.013*	0.001	0.017$^{\cdot}$	0.010	0.008	0.023*	0.018$^{\cdot}$	0.006	0.020
	(0.006)	(0.007)	(0.009)	(0.007)	(0.009)	(0.011)	(0.009)	(0.010)	(0.013)
Prior agrivoltaics preference	0.021**	0.012	0.020$^{\cdot}$	0.019$^{\cdot}$	0.043***	0.033**	0.015	0.052***	0.052***
	(0.008)	(0.008)	(0.011)	(0.010)	(0.010)	(0.012)	(0.012)	(0.011)	(0.014)
Degree of urbanization	−0.024	0.018	−0.019	0.011	0.011	−0.032	−0.009	−0.035	−0.047$^{\cdot}$
	(0.022)	(0.017)	(0.020)	(0.023)	(0.022)	(0.023)	(0.028)	(0.025)	(0.027)
Interactions									
NIMBY × Pot. in Circle1	0.393***	0.189**	0.165*	−0.019	0.079	−0.016	0.154	0.089	0.097
	(0.085)	(0.066)	(0.080)	(0.097)	(0.086)	(0.093)	(0.104)	(0.097)	(0.108)
NIMBY × Pot. in Circle2	0.010	−0.156	0.095	0.469**	−0.007	0.059	0.047	−0.060	0.065
	(0.133)	(0.096)	(0.134)	(0.148)	(0.116)	(0.170)	(0.154)	(0.141)	(0.199)
NIMBY × Pot. in Circle3	−0.074	0.139	−0.171	−0.227$^{\cdot}$	0.096	−0.043	0.285*	0.140	0.138
	(0.120)	(0.086)	(0.121)	(0.131)	(0.111)	(0.161)	(0.140)	(0.135)	(0.192)
Observations	818	807	520	818	807	520	818	807	520
R^2	0.292	0.109	0.108	0.190	0.144	0.099	0.161	0.162	0.125
Adj. R^2	0.281	0.094	0.085	0.177	0.130	0.076	0.148	0.148	0.102
RMSE	0.277	0.297	0.321	0.347	0.381	0.388	0.447	0.457	0.458

Table 14: CC1: Knowledge of potential in circle 1 (0m-500m, binary), CC2: Knowledge of potential in circle 2 (500m-1500m, binary), CC3: Knowledge of potential in circle 3 (1500m-4500m, binary). Statistical significance was derived from linear least squares regression and two-sided tests. Respective standard errors are reported in brackets and significant effects are indicated with: *** for $p < 0.001$, ** for $p < 0.01$, * for $p < 0.05$ and $\cdot$ for $p < 0.1$.

SI Table 15

	DV1, Left	DV1, Centre	DV1, Right	DV2, Left	DV2, Centre	DV2, Right
Intercept	1.289***	1.361***	0.981**	1.345***	1.360***	1.121***
	(0.322)	(0.281)	(0.311)	(0.308)	(0.271)	(0.322)
Treatment	−0.108	−0.063	−0.113	−0.055	−0.046	−0.087
	(0.084)	(0.089)	(0.114)	(0.076)	(0.093)	(0.105)
Realistic agrivoltaics potential						
Potential in circle 1	0.279·	0.074	−0.260	0.271·	0.003	0.041
	(0.156)	(0.158)	(0.245)	(0.153)	(0.162)	(0.213)
Potential in circle 2	−0.541·	−0.011	0.085	−0.439	−0.033	−0.045
	(0.295)	(0.173)	(0.261)	(0.317)	(0.176)	(0.217)
Potential in circle 3	0.104	0.057	−0.104	0.051	0.109	−0.357·
	(0.206)	(0.187)	(0.235)	(0.220)	(0.175)	(0.191)
Controls						
Age	−0.003	−0.004	0.003	−0.003	−0.001	0.003
	(0.002)	(0.002)	(0.003)	(0.002)	(0.002)	(0.003)
Gender: Male	−0.012	−0.010	0.080	−0.018	0.003	0.015
	(0.078)	(0.073)	(0.095)	(0.068)	(0.073)	(0.088)
Environmental score	0.085*	0.147***	0.163***	0.070·	0.166***	0.138**
	(0.042)	(0.040)	(0.048)	(0.037)	(0.040)	(0.043)
Familiarity with agrivoltaics	0.058*	0.034	0.019	0.041*	0.037	−0.019
	(0.024)	(0.024)	(0.028)	(0.020)	(0.024)	(0.026)
Prior agrivoltaics preference	0.631***	0.543***	0.600***	0.653***	0.534***	0.622***
	(0.035)	(0.032)	(0.038)	(0.033)	(0.031)	(0.036)
Degree of urbanization	−0.092	−0.001	−0.190**	−0.034	−0.053	−0.116·
	(0.074)	(0.057)	(0.067)	(0.060)	(0.059)	(0.064)
Interactions						
NIMBY × Pot. in Circle1	−0.564**	0.084	0.089	−0.382*	0.150	−0.212
	(0.196)	(0.193)	(0.280)	(0.184)	(0.186)	(0.253)
NIMBY × Pot. in Circle2	0.477	−0.055	−0.056	0.399	−0.095	−0.155
	(0.354)	(0.260)	(0.343)	(0.368)	(0.238)	(0.316)
NIMBY × Pot. in Circle3	0.160	−0.145	0.216	0.284	−0.191	0.647*
	(0.274)	(0.265)	(0.318)	(0.282)	(0.229)	(0.291)
Observations	818	807	520	818	807	520
R^2	0.387	0.398	0.508	0.458	0.403	0.543
Adj. R^2	0.378	0.388	0.495	0.449	0.394	0.531
RMSE	1.071	1.022	1.046	0.935	1.013	0.979

Table 15: DV1: Personal advantage from agrivoltaics (1-7 Likert scale), DV2: Advantage for Switzerland (1-7 Likert scale). Statistical significance was derived from linear least squares regression and two-sided tests. Respective standard errors are reported in brackets and significant effects are indicated with: *** for $p < 0.001$, ** for $p < 0.01$, * for $p < 0.05$ and · for $p < 0.1$.

SI Table 16

	DV3, Left	DV3, Centre	DV3, Right	DV4, Left	DV4, Centre	DV4, Right	DV5, Left	DV5, Centre	DV5, Right	DV6, Left	DV6, Centre	DV6, Right	DV7, Left	DV7, Centre	DV7, Right
Intercept	1.655***	1.340***	0.565	1.020**	0.507·	−0.082	0.240	0.170	−0.302	1.130***	0.709**	0.242	0.733*	0.389	−0.036
	(0.331)	(0.284)	(0.357)	(0.361)	(0.304)	(0.364)	(0.431)	(0.362)	(0.406)	(0.279)	(0.232)	(0.311)	(0.340)	(0.273)	(0.322)
Treatment	−0.099	−0.060	−0.118	−0.158·	−0.045	−0.262·	−0.186·	−0.012	−0.126	−0.133·	−0.113	−0.051	−0.083	−0.076	−0.207
	(0.074)	(0.098)	(0.121)	(0.085)	(0.104)	(0.137)	(0.110)	(0.123)	(0.170)	(0.069)	(0.081)	(0.111)	(0.081)	(0.091)	(0.126)
Realistic agrivoltaics potential															
Potential in circle 1	0.171	0.190	−0.599*	0.125	0.106	−0.556*	0.112	0.001	−0.393	0.160	0.018	−0.173	0.012	0.171	−0.639**
	(0.182)	(0.156)	(0.250)	(0.209)	(0.194)	(0.242)	(0.223)	(0.259)	(0.285)	(0.139)	(0.164)	(0.197)	(0.182)	(0.170)	(0.210)
Potential in circle 2	−0.202	0.020	0.669*	−0.292	0.269	0.585·	−0.334	0.547·	0.117	−0.130	−0.003	0.018	−0.437	0.180	0.767*
	(0.270)	(0.199)	(0.281)	(0.305)	(0.242)	(0.304)	(0.349)	(0.327)	(0.480)	(0.207)	(0.187)	(0.233)	(0.293)	(0.219)	(0.320)
Potential in circle 3	−0.156	0.104	−0.628*	−0.193	−0.058	−0.818**	−0.102	−0.343	−0.377	−0.243	0.022	−0.048	−0.027	−0.148	−0.607*
	(0.212)	(0.184)	(0.252)	(0.253)	(0.245)	(0.278)	(0.378)	(0.342)	(0.479)	(0.195)	(0.222)	(0.235)	(0.214)	(0.218)	(0.300)
Controls															
Age	−0.002	0.000	0.007·	−0.005*	−0.002	0.004	−0.008*	−0.006*	0.002	0.002	0.004	0.010**	−0.002	0.003	0.009*
	(0.002)	(0.002)	(0.004)	(0.002)	(0.003)	(0.004)	(0.003)	(0.003)	(0.005)	(0.002)	(0.002)	(0.003)	(0.002)	(0.003)	(0.004)
Gender: Male	−0.037	0.109	0.048	0.164*	0.442***	0.314*	0.289**	0.618***	0.443**	0.029	0.097	0.029	0.213**	0.287***	0.102
	(0.067)	(0.074)	(0.109)	(0.077)	(0.084)	(0.126)	(0.097)	(0.099)	(0.145)	(0.062)	(0.065)	(0.091)	(0.072)	(0.075)	(0.107)
Environmental score	0.118**	0.164***	0.180***	0.101*	0.156***	0.164**	0.133*	0.158**	0.128*	0.072*	0.208***	0.176***	0.121**	0.217***	0.208***
	(0.038)	(0.040)	(0.047)	(0.042)	(0.043)	(0.054)	(0.053)	(0.051)	(0.059)	(0.033)	(0.035)	(0.042)	(0.044)	(0.040)	(0.047)
Familiarity with agrivoltaics	0.026	0.058*	−0.030	0.024	0.064*	−0.011	0.036	0.077*	−0.019	0.048**	0.063**	0.031	0.063**	0.067**	0.012
	(0.021)	(0.024)	(0.033)	(0.024)	(0.027)	(0.038)	(0.029)	(0.032)	(0.043)	(0.019)	(0.020)	(0.026)	(0.024)	(0.024)	(0.031)
Prior agrivoltaics preference	0.580***	0.507***	0.673***	0.673***	0.567***	0.694***	0.693***	0.542***	0.629***	0.658***	0.555***	0.642***	0.645***	0.549***	0.638***
	(0.035)	(0.033)	(0.035)	(0.035)	(0.032)	(0.039)	(0.039)	(0.035)	(0.043)	(0.031)	(0.031)	(0.034)	(0.034)	(0.031)	(0.037)
Degree of urbanization	−0.112·	−0.079	−0.257**	−0.180*	−0.114·	−0.129	−0.172·	−0.109	0.003	−0.012	−0.029	−0.123·	−0.052	−0.145*	−0.150*
	(0.058)	(0.061)	(0.078)	(0.077)	(0.068)	(0.081)	(0.089)	(0.076)	(0.088)	(0.048)	(0.056)	(0.064)	(0.055)	(0.068)	(0.073)
Interactions															
NIMBY × Pot. in Circle1	−0.010	−0.096	0.441	−0.069	−0.065	0.278	−0.417	0.191	−0.088	−0.362*	0.021	−0.152	−0.253	−0.239	0.246
	(0.217)	(0.187)	(0.293)	(0.246)	(0.233)	(0.296)	(0.270)	(0.300)	(0.341)	(0.177)	(0.182)	(0.231)	(0.232)	(0.207)	(0.262)
NIMBY × Pot. in Circle2	−0.026	0.070	−0.628	0.171	−0.164	−0.213	0.137	−0.468	0.354	0.267	−0.036	−0.020	0.574	−0.149	−0.297
	(0.334)	(0.247)	(0.420)	(0.366)	(0.308)	(0.435)	(0.453)	(0.387)	(0.564)	(0.255)	(0.228)	(0.297)	(0.385)	(0.294)	(0.403)
NIMBY × Pot. in Circle3	0.263	−0.249	0.709·	0.386	−0.318	0.467	0.371	−0.227	−0.162	0.343	−0.130	0.242	0.155	0.066	0.301
	(0.268)	(0.242)	(0.389)	(0.310)	(0.313)	(0.420)	(0.458)	(0.393)	(0.563)	(0.231)	(0.257)	(0.294)	(0.308)	(0.283)	(0.389)
Observations	818	807	520	818	807	520	818	807	520	818	807	520	818	807	520
R^2	0.406	0.377	0.527	0.418	0.385	0.473	0.348	0.314	0.348	0.506	0.497	0.573	0.439	0.430	0.523
Adj. R^2	0.396	0.366	0.515	0.408	0.375	0.460	0.338	0.303	0.331	0.498	0.488	0.562	0.430	0.421	0.511
RMSE	0.944	1.034	1.161	1.080	1.168	1.303	1.344	1.381	1.515	0.853	0.898	0.983	0.997	1.048	1.133

Table 16: DV3: Expansion <1ha, DV4: Expansion <5ha, DV5: Expansion <10ha, DV6: Expansion in Switzerland, DV7: Expanion in neighbourhood. Statistical significance was derived from linear least squares regression and two-sided tests. Respective standard errors are reported in brackets and significant effects are indicated with: *** for $p < 0.001$, ** for $p < 0.01$, * for $p < 0.05$ and · for $p < 0.1$.

SI Table 17

	DV8, Left	DV8, Centre	DV8, Right	DV9, Left	DV9, Centre	DV9, Right	DV10, Left	DV10, Centre	DV10, Right	DV11, Left	DV11, Centre	DV11, Right	DV12, Left	DV12, Centre	DV12, Right
Intercept	−0.200	−0.620*	−0.452	0.556	−0.620*	0.145	1.641***	1.214***	1.232**	1.264**	1.419***	0.814·	0.943*	1.075***	0.429
	(0.362)	(0.305)	(0.405)	(0.391)	(0.305)	(0.440)	(0.384)	(0.296)	(0.414)	(0.383)	(0.323)	(0.437)	(0.418)	(0.309)	(0.413)
Treatment	−0.062	−0.106	−0.099	−0.080	−0.106	−0.205	0.002	−0.115	−0.205	−0.093	−0.095	−0.110	−0.039	−0.204·	0.075
	(0.086)	(0.098)	(0.151)	(0.096)	(0.098)	(0.169)	(0.085)	(0.095)	(0.149)	(0.099)	(0.104)	(0.164)	(0.093)	(0.107)	(0.171)
Realistic agrivoltaics potential															
Potential in circle 1	0.017	−0.135	−0.320	0.126	−0.135	−0.262	0.490**	−0.191	−0.262	0.187	−0.203	−0.154	0.350	−0.192	0.210
	(0.187)	(0.231)	(0.254)	(0.216)	(0.231)	(0.309)	(0.156)	(0.225)	(0.322)	(0.197)	(0.230)	(0.318)	(0.235)	(0.213)	(0.331)
Potential in circle 2	−0.171	−0.173	0.559	−0.382	−0.173	0.426	−0.507*	0.331	0.263	0.151	0.202	0.295	−0.175	0.057	0.343
	(0.223)	(0.275)	(0.356)	(0.357)	(0.275)	(0.363)	(0.232)	(0.261)	(0.362)	(0.275)	(0.284)	(0.357)	(0.325)	(0.280)	(0.394)
Potential in circle 3	0.076	0.244	−0.559	0.118	0.244	−0.584·	−0.043	0.305	−0.179	−0.383	0.343	−0.132	−0.267	0.321	−0.515
	(0.221)	(0.267)	(0.380)	(0.376)	(0.267)	(0.341)	(0.235)	(0.264)	(0.344)	(0.288)	(0.271)	(0.350)	(0.389)	(0.263)	(0.391)
Controls															
Age	0.001	0.004	0.014***	0.001	0.004	0.008	0.001	0.002	−0.000	0.008**	0.003	−0.002	−0.006*	−0.003	−0.003
	(0.002)	(0.003)	(0.004)	(0.003)	(0.003)	(0.005)	(0.002)	(0.003)	(0.004)	(0.003)	(0.003)	(0.005)	(0.002)	(0.003)	(0.005)
Gender: Male	0.005	0.171*	−0.001	0.144·	0.171*	0.200	−0.084	0.076	0.010	−0.039	−0.012	0.045	−0.172*	0.017	0.041
	(0.076)	(0.086)	(0.129)	(0.087)	(0.086)	(0.141)	(0.074)	(0.082)	(0.117)	(0.086)	(0.088)	(0.123)	(0.086)	(0.090)	(0.130)
Environmental score	0.183***	0.255***	0.226***	0.071	0.255***	0.181**	0.079·	0.167***	0.132*	0.100*	0.077·	0.153**	0.179***	0.190***	0.229***
	(0.048)	(0.046)	(0.059)	(0.052)	(0.046)	(0.063)	(0.045)	(0.045)	(0.055)	(0.051)	(0.046)	(0.056)	(0.050)	(0.048)	(0.059)
Familiarity with agrivoltaics	0.058*	0.080**	0.033	0.052*	0.080**	0.043	0.037·	0.040	0.013	0.013	0.046·	0.051	0.043·	0.074*	0.021
	(0.024)	(0.029)	(0.037)	(0.026)	(0.029)	(0.042)	(0.023)	(0.025)	(0.034)	(0.027)	(0.028)	(0.038)	(0.026)	(0.029)	(0.040)
Prior agrivoltaics preference	0.753***	0.648***	0.649***	0.657***	0.648***	0.572***	0.606***	0.551***	0.640***	0.482***	0.438***	0.515***	0.558***	0.439***	0.547***
	(0.038)	(0.032)	(0.042)	(0.037)	(0.032)	(0.045)	(0.040)	(0.035)	(0.043)	(0.038)	(0.037)	(0.044)	(0.037)	(0.039)	(0.044)
Degree of urbanization	−0.116·	−0.046	−0.274***	−0.088	−0.046	−0.087	−0.102·	−0.106·	−0.162*	−0.150*	−0.083	−0.150·	−0.024	0.065	−0.142·
	(0.060)	(0.065)	(0.082)	(0.077)	(0.065)	(0.093)	(0.062)	(0.064)	(0.080)	(0.071)	(0.075)	(0.084)	(0.078)	(0.074)	(0.085)
Interactions															
NIMBY × Pot. in Circle1	−0.071	0.189	0.102	−0.358	0.189	−0.076	−0.532**	0.227	0.302	−0.287	0.131	0.044	−0.544·	0.253	−0.294
	(0.239)	(0.259)	(0.309)	(0.260)	(0.259)	(0.358)	(0.201)	(0.255)	(0.360)	(0.240)	(0.263)	(0.355)	(0.279)	(0.249)	(0.370)
NIMBY × Pot. in Circle2	0.035	0.050	−0.377	0.297	0.050	−0.321	0.579*	−0.390	−0.200	−0.140	−0.108	−0.124	0.511	−0.151	−0.266
	(0.286)	(0.348)	(0.435)	(0.417)	(0.348)	(0.477)	(0.292)	(0.311)	(0.437)	(0.366)	(0.347)	(0.447)	(0.383)	(0.339)	(0.501)
NIMBY × Pot. in Circle3	0.026	−0.139	0.485	0.005	−0.139	0.468	0.187	−0.389	0.187	0.683·	−0.299	0.248	0.287	−0.380	0.441
	(0.269)	(0.330)	(0.454)	(0.407)	(0.330)	(0.438)	(0.287)	(0.328)	(0.413)	(0.354)	(0.351)	(0.420)	(0.433)	(0.324)	(0.485)
Observations	818	807	520	818	807	520	818	807	520	818	807	520	818	807	520
R^2	0.475	0.439	0.453	0.347	0.439	0.329	0.374	0.371	0.422	0.231	0.238	0.305	0.313	0.253	0.323
Adj. R^2	0.466	0.430	0.439	0.336	0.430	0.312	0.364	0.361	0.408	0.219	0.225	0.287	0.302	0.241	0.305
RMSE	1.066	1.196	1.352	1.202	1.196	1.495	1.033	1.142	1.282	1.181	1.225	1.383	1.164	1.260	1.451

Table 17: DV8: General policy support , DV9: Simplified approval procedures, DV10: Advisory services for farmers, DV11: Increased one-off payments, DV12: Financial support for large projects. Statistical significance was derived from linear least squares regression and two-sided tests. Respective standard errors are reported in brackets and significant effects are indicated with: *** for $p < 0.001$, ** for $p < 0.01$, * for $p < 0.05$ and · for $p < 0.1$.

Section C2 – By degree of urbanization

SI Table 18

	CC1, Urban	CC1, Suburban	CC1, Rural	CC2, Urban	CC2, Suburban	CC2, Rural	CC3, Urban	CC3, Suburban	CC3, Rural
Intercept	0.046	0.206	−0.109	0.094	0.156	−0.153	0.234^{*}	0.075	−0.170
	(0.064)	(0.199)	(0.178)	(0.082)	(0.237)	(0.201)	(0.100)	(0.229)	(0.241)
Treatment	−0.006	0.027	0.055	-0.096^{***}	−0.027	−0.055	-0.264^{***}	-0.164^{**}	−0.091
	(0.015)	(0.047)	(0.044)	(0.021)	(0.055)	(0.050)	(0.026)	(0.056)	(0.059)
Realistic agrivoltaics potential									
Potential in circle 1	0.304^{***}	0.215^{***}	0.122^{**}	0.160^{***}	0.127^{*}	0.117^{*}	0.015	−0.010	0.019
	(0.035)	(0.046)	(0.044)	(0.034)	(0.054)	(0.051)	(0.037)	(0.059)	(0.059)
Potential in circle 2	−0.011	0.051	0.013	0.226^{***}	0.195^{*}	0.330^{*}	$0.108^{\cdot}$	$0.133^{\cdot}$	0.367^{**}
	(0.046)	(0.071)	(0.130)	(0.057)	(0.077)	(0.132)	(0.060)	(0.077)	(0.140)
Potential in circle 3	−0.025	0.002	0.106	0.025	−0.044	−0.113	0.323^{***}	$0.145^{\cdot}$	−0.032
	(0.032)	(0.071)	(0.147)	(0.044)	(0.077)	(0.149)	(0.053)	(0.078)	(0.152)
Controls									
Age	-0.001^{**}	$-0.003^{\cdot}$	0.000	-0.002^{**}	−0.002	-0.004^{*}	−0.001	−0.001	−0.002
	(0.000)	(0.001)	(0.001)	(0.001)	(0.002)	(0.002)	(0.001)	(0.002)	(0.002)
Gender: Male	0.018	−0.025	0.052	0.027	$0.099^{\cdot}$	$0.088^{\cdot}$	0.028	0.147^{**}	0.149^{**}
	(0.014)	(0.044)	(0.043)	(0.018)	(0.051)	(0.049)	(0.024)	(0.052)	(0.056)
Environmental score	−0.007	−0.038	0.026	−0.015	−0.010	$0.040^{\cdot}$	0.002	0.036	$0.056^{\cdot}$
	(0.008)	(0.023)	(0.022)	(0.010)	(0.026)	(0.022)	(0.012)	(0.026)	(0.029)
Familiarity with agrivoltaics	$0.008^{\cdot}$	0.004	0.000	$0.011^{\cdot}$	−0.008	$0.029^{\cdot}$	0.014^{*}	−0.009	0.016
	(0.004)	(0.014)	(0.014)	(0.006)	(0.015)	(0.016)	(0.007)	(0.015)	(0.018)
Prior agrivoltaics preference	0.015^{**}	0.052^{**}	−0.002	0.029^{***}	0.054^{**}	0.036^{*}	0.037^{***}	0.061^{**}	0.040^{*}
	(0.005)	(0.016)	(0.014)	(0.007)	(0.019)	(0.015)	(0.008)	(0.020)	(0.018)
Political orientation	−0.001	−0.019	−0.004	0.007	−0.024	−0.012	$0.012^{\cdot}$	−0.016	−0.003
	(0.004)	(0.013)	(0.011)	(0.005)	(0.015)	(0.012)	(0.006)	(0.016)	(0.015)
Observations	1545	345	255	1545	345	255	1545	345	255
R^2	0.135	0.142	0.086	0.101	0.125	0.190	0.129	0.167	0.171
Adj. R^2	0.129	0.116	0.048	0.095	0.099	0.157	0.123	0.142	0.137
RMSE	0.270	0.392	0.332	0.352	0.448	0.376	0.455	0.463	0.445

Table 18: CC1: Knowledge of potential in circle 1 (0m-500m, binary), CC2: Knowledge of potential in circle 2 (500m-1500m, binary), CC3: Knowledge of potential in circle 3 (1500m-4500m, binary). Statistical significance was derived from linear least squares regression and two-sided tests. Respective standard errors are reported in brackets and significant effects are indicated with: *** for $p < 0.001$, ** for $p < 0.01$, * for $p < 0.05$ and · for $p < 0.1$.

SI Table 19

	CC1, low, Urban	CC1, low, Suburban	CC1, low, Rural	CC1, high, Urban	CC1, high, Suburban	CC1, high, Rural
Intercept	0.055	0.210	0.100	0.103	0.363	-0.145
	(0.058)	(0.234)	(0.126)	(0.266)	(0.288)	(0.309)
Treatment	-0.041^{**}	-0.077	-0.054	0.239^{***}	$0.129^{\cdot}$	0.174^{*}
	(0.014)	(0.058)	(0.050)	(0.065)	(0.069)	(0.075)
Controls						
Age	-0.000	0.001	0.000	-0.008^{***}	-0.006^{**}	-0.001
	(0.000)	(0.001)	(0.001)	(0.002)	(0.002)	(0.003)
Gender: Male	0.019	-0.009	0.003	0.013	-0.057	0.075
	(0.012)	(0.050)	(0.044)	(0.060)	(0.066)	(0.081)
Environmental score	-0.008	-0.022	0.007	0.042	-0.030	0.058
	(0.007)	(0.028)	(0.023)	(0.034)	(0.033)	(0.036)
Familiarity with agrivoltaics	0.010^{*}	0.001	0.010	0.002	0.006	-0.009
	(0.004)	(0.016)	(0.015)	(0.019)	(0.019)	(0.025)
Prior agrivoltaics preference	0.005	-0.004	-0.005	0.062^{*}	0.092^{***}	-0.002
	(0.005)	(0.021)	(0.017)	(0.025)	(0.022)	(0.024)
Political orientation	0.003	-0.001	-0.011	-0.014	-0.027	0.002
	(0.003)	(0.016)	(0.011)	(0.016)	(0.018)	(0.019)
Observations	1330	158	137	215	187	118
R^2	0.021	0.033	0.028	0.183	0.162	0.077
Adj. R^2	0.016	-0.013	-0.025	0.156	0.129	0.018
RMSE	0.220	0.287	0.252	0.438	0.442	0.407

Table 19: CC1: Knowledge of potential in circle 1 (0m-500m, binary). Statistical significance was derived from linear least squares regression and two-sided tests. Respective standard errors are reported in brackets and significant effects are indicated with: *** for $p < 0.001$, ** for $p < 0.01$, * for $p < 0.05$ and $\cdot$ for $p < 0.1$.

SI Table 20

	CC2, low, Urban	CC2, low, Suburban	CC2, low, Rural	CC2, high, Urban	CC2, high, Suburban	CC2, high, Rural
Intercept	0.130	0.121	0.030	−0.171	0.285	−0.581
	(0.083)	(0.313)	(0.212)	(0.433)	(0.332)	(0.383)
Treatment	−0.098***	−0.075	−0.082	0.045	0.089	0.062
	(0.021)	(0.071)	(0.055)	(0.099)	(0.086)	(0.115)
Controls						
Age	−0.001·	0.001	−0.002	−0.007*	−0.006*	−0.008·
	(0.001)	(0.002)	(0.002)	(0.003)	(0.003)	(0.004)
Gender: Male	0.016	0.026	0.087·	0.134	0.180*	0.088
	(0.018)	(0.064)	(0.052)	(0.098)	(0.077)	(0.107)
Environmental score	−0.020*	−0.026	0.028	0.042	0.044	0.127*
	(0.010)	(0.033)	(0.023)	(0.056)	(0.037)	(0.050)
Familiarity with agrivoltaics	0.012*	0.039*	0.017	0.003	−0.061**	0.020
	(0.006)	(0.019)	(0.017)	(0.034)	(0.021)	(0.034)
Prior agrivoltaics preference	0.025***	0.027	0.008	0.111**	0.082**	0.137***
	(0.007)	(0.027)	(0.016)	(0.042)	(0.026)	(0.028)
Political orientation	0.009·	−0.010	−0.004	0.016	−0.031	−0.015
	(0.005)	(0.020)	(0.012)	(0.029)	(0.022)	(0.028)
Observations	1445	194	197	100	151	58
R^2	0.040	0.058	0.056	0.154	0.218	0.397
Adj. R^2	0.036	0.023	0.021	0.090	0.180	0.312
RMSE	0.343	0.418	0.352	0.478	0.453	0.414

Table 20: CC2: Knowledge of potential in circle 2 (500m-1500m, binary). Statistical significance was derived from linear least squares regression and two-sided tests. Respective standard errors are reported in brackets and significant effects are indicated with: *** for $p < 0.001$, ** for $p < 0.01$, * for $p < 0.05$ and · for $p < 0.1$.

SI Table 21

	CC3, low, Urban	CC3, low, Suburban	CC3, low, Rural	CC3, high, Urban	CC3, high, Suburban	CC3, high, Rural
Intercept	0.254*	−0.150	−0.109	0.304	0.557	0.086
	(0.103)	(0.277)	(0.273)	(0.355)	(0.403)	(0.548)
Treatment	−0.289***	−0.178*	−0.139*	0.032	−0.124	0.256·
	(0.027)	(0.075)	(0.066)	(0.087)	(0.092)	(0.127)
Controls						
Age	−0.001	0.001	−0.001	−0.003	−0.004	−0.005
	(0.001)	(0.002)	(0.002)	(0.003)	(0.003)	(0.005)
Gender: Male	0.024	0.080	0.138*	0.111	0.242**	0.093
	(0.025)	(0.069)	(0.063)	(0.088)	(0.079)	(0.133)
Environmental score	−0.002	0.059·	0.043	0.061	0.004	0.069
	(0.012)	(0.033)	(0.030)	(0.043)	(0.043)	(0.066)
Familiarity with agrivoltaics	0.018*	0.006	0.016	−0.029	−0.032	−0.027
	(0.007)	(0.019)	(0.021)	(0.031)	(0.023)	(0.039)
Prior agrivoltaics preference	0.038***	0.044·	0.033·	0.041	0.087**	0.108*
	(0.009)	(0.026)	(0.020)	(0.035)	(0.029)	(0.044)
Political orientation	0.013·	−0.000	0.002	0.010	−0.030	−0.034
	(0.007)	(0.020)	(0.017)	(0.025)	(0.026)	(0.031)
Observations	1428	211	206	117	134	49
R^2	0.102	0.087	0.072	0.072	0.187	0.312
Adj. R^2	0.097	0.055	0.039	0.012	0.141	0.195
RMSE	0.454	0.473	0.446	0.445	0.446	0.442

Table 21: CC1: Knowledge of potential in circle 3 (1500m-4500m, binary). Statistical significance was derived from linear least squares regression and two-sided tests. Respective standard errors are reported in brackets and significant effects are indicated with: *** for $p < 0.001$, ** for $p < 0.01$, * for $p < 0.05$ and · for $p < 0.1$.

SI Table 22

	DV1, Urban	DV1, Suburban	DV1, Rural	DV2, Urban	DV2, Suburban	DV2, Rural
Intercept	1.317***	2.074***	−0.664	1.387***	2.132***	0.132
	(0.224)	(0.526)	(0.678)	(0.224)	(0.457)	(0.646)
Treatment	−0.092·	−0.146	−0.080	−0.034	−0.113	−0.160
	(0.055)	(0.106)	(0.152)	(0.052)	(0.106)	(0.141)
Realistic agrivoltaics potential						
Potential in circle 1	−0.100	−0.108	0.132	0.033	−0.141	0.149
	(0.082)	(0.124)	(0.147)	(0.072)	(0.113)	(0.142)
Potential in circle 2	0.008	−0.071	−0.118	−0.123	0.037	−0.086
	(0.124)	(0.151)	(0.413)	(0.119)	(0.135)	(0.391)
Potential in circle 3	0.050	0.033	0.083	0.124	−0.017	0.026
	(0.109)	(0.144)	(0.412)	(0.096)	(0.134)	(0.384)
Controls						
Age	−0.003·	−0.002	0.006	−0.002	−0.000	0.001
	(0.002)	(0.004)	(0.005)	(0.002)	(0.003)	(0.005)
Gender: Male	0.009	0.001	0.112	0.002	0.080	−0.044
	(0.055)	(0.114)	(0.155)	(0.051)	(0.100)	(0.145)
Environmental score	0.126***	0.033	0.244**	0.125***	0.064	0.194*
	(0.029)	(0.057)	(0.082)	(0.027)	(0.053)	(0.077)
Familiarity with agrivoltaics	0.034*	0.071*	0.033	0.029·	0.035	−0.002
	(0.017)	(0.033)	(0.049)	(0.016)	(0.029)	(0.049)
Prior agrivoltaics preference	0.571***	0.602***	0.661***	0.576***	0.595***	0.713***
	(0.024)	(0.042)	(0.051)	(0.024)	(0.038)	(0.048)
Political orientation	0.005	−0.106**	0.021	−0.004	−0.133***	0.012
	(0.014)	(0.035)	(0.039)	(0.014)	(0.029)	(0.035)
Observations	1545	345	255	1545	345	255
R^2	0.393	0.485	0.522	0.430	0.541	0.574
Adj. R^2	0.389	0.470	0.502	0.426	0.528	0.556
RMSE	1.041	1.001	1.145	0.970	0.903	1.089

Table 22: DV1: Personal advantage from agrivoltaics (1-7 Likert scale), DV2: Advantage for Switzerland (1-7 Likert scale). Statistical significance was derived from linear least squares regression and two-sided tests. Respective standard errors are reported in brackets and significant effects are indicated with: *** for $p < 0.001$, ** for $p < 0.01$, * for $p < 0.05$ and · for $p < 0.1$.

SI Table 23

	DV3, Urban	DV3, Suburban	DV3, Rural	DV4, Urban	DV4, Suburban	DV4, Rural	DV5, Urban	DV5, Suburban	DV5, Rural	DV6, Urban	DV6, Suburban	DV6, Rural	DV7, Urban	DV7, Suburban	DV7, Rural
Intercept	1.418***	1.519**	0.059	0.777**	1.898**	−0.171	0.457	1.697*	−0.439	0.886***	1.369**	0.603	0.808***	0.877$^{\cdot}$	−1.451*
	(0.232)	(0.586)	(0.633)	(0.249)	(0.614)	(0.649)	(0.300)	(0.702)	(0.775)	(0.199)	(0.428)	(0.662)	(0.227)	(0.457)	(0.679)
Treatment	−0.103$^{\cdot}$	−0.181	0.175	−0.178**	−0.260$^{\cdot}$	0.213	−0.201**	−0.262	0.308	−0.103*	−0.309**	0.027	−0.139*	−0.241*	0.180
	(0.055)	(0.123)	(0.158)	(0.059)	(0.144)	(0.174)	(0.075)	(0.166)	(0.188)	(0.048)	(0.095)	(0.142)	(0.054)	(0.120)	(0.162)
Realistic agrivoltaics potential															
Potential in circle 1	0.064	−0.086	−0.023	0.021	0.004	−0.241	−0.056	−0.174	−0.170	−0.068	−0.211*	−0.047	−0.106	−0.329*	−0.235
	(0.081)	(0.156)	(0.153)	(0.090)	(0.169)	(0.168)	(0.107)	(0.179)	(0.191)	(0.070)	(0.106)	(0.131)	(0.082)	(0.135)	(0.173)
Potential in circle 2	−0.017	0.188	0.168	−0.048	0.350	0.580*	−0.118	0.439$^{\cdot}$	0.551	0.031	0.102	−0.011	0.008	0.309$^{\cdot}$	0.345
	(0.115)	(0.186)	(0.276)	(0.126)	(0.220)	(0.291)	(0.170)	(0.242)	(0.449)	(0.096)	(0.125)	(0.255)	(0.136)	(0.168)	(0.514)
Potential in circle 3	−0.022	−0.267	0.049	−0.113	−0.602**	−0.139	−0.166	−0.629*	−0.173	0.037	−0.142	0.231	−0.112	−0.155	−0.106
	(0.105)	(0.179)	(0.317)	(0.121)	(0.223)	(0.332)	(0.151)	(0.245)	(0.476)	(0.093)	(0.128)	(0.276)	(0.122)	(0.159)	(0.512)
Controls															
Age	0.000	0.005	0.004	−0.003	0.003	−0.004	−0.007**	0.004	−0.007	0.004*	0.008*	0.004	0.000	0.008$^{\cdot}$	0.010$^{\cdot}$
	(0.002)	(0.004)	(0.005)	(0.002)	(0.004)	(0.005)	(0.002)	(0.005)	(0.006)	(0.001)	(0.003)	(0.005)	(0.002)	(0.004)	(0.005)
Gender: Male	0.017	0.127	0.139	0.354***	0.242$^{\cdot}$	0.216	0.521***	0.356*	0.236	0.077	0.093	−0.066	0.266***	0.106	0.111
	(0.052)	(0.132)	(0.154)	(0.059)	(0.144)	(0.176)	(0.073)	(0.160)	(0.195)	(0.047)	(0.100)	(0.138)	(0.053)	(0.122)	(0.169)
Environmental score	0.131***	0.202**	0.258***	0.135***	0.029	0.232**	0.144***	−0.070	0.229*	0.153***	0.125*	0.171*	0.158***	0.165**	0.346***
	(0.027)	(0.072)	(0.077)	(0.030)	(0.074)	(0.086)	(0.037)	(0.080)	(0.089)	(0.025)	(0.050)	(0.071)	(0.028)	(0.061)	(0.082)
Familiarity with agrivoltaics	0.032*	0.007	−0.053	0.040*	0.011	−0.046	0.055*	0.013	−0.048	0.051***	0.067*	−0.009	0.051**	0.074*	0.016
	(0.016)	(0.040)	(0.048)	(0.018)	(0.045)	(0.058)	(0.022)	(0.048)	(0.063)	(0.014)	(0.028)	(0.050)	(0.016)	(0.037)	(0.058)
Prior agrivoltaics preference	0.579***	0.529***	0.650***	0.646***	0.616***	0.664***	0.625***	0.614***	0.605***	0.608***	0.602***	0.664***	0.605***	0.579***	0.657***
	(0.023)	(0.055)	(0.052)	(0.024)	(0.054)	(0.050)	(0.027)	(0.053)	(0.054)	(0.022)	(0.039)	(0.054)	(0.022)	(0.049)	(0.053)
Political orientation	−0.037**	−0.108*	−0.062	−0.076***	−0.197***	−0.026	−0.083***	−0.182***	0.012	−0.032**	−0.110***	−0.044	−0.059***	−0.111***	−0.017
	(0.014)	(0.047)	(0.038)	(0.016)	(0.044)	(0.041)	(0.020)	(0.047)	(0.044)	(0.012)	(0.028)	(0.033)	(0.014)	(0.033)	(0.039)
Observations	1545	345	255	1545	345	255	1545	345	255	1545	345	255	1545	345	255
R^2	0.434	0.402	0.540	0.448	0.400	0.484	0.351	0.333	0.393	0.516	0.593	0.584	0.466	0.475	0.533
Adj. R^2	0.430	0.384	0.521	0.444	0.383	0.463	0.347	0.313	0.368	0.513	0.581	0.567	0.463	0.460	0.514
RMSE	0.994	1.141	1.183	1.116	1.291	1.315	1.386	1.424	1.430	0.892	0.847	1.043	1.013	1.071	1.238

Table 23: DV3: Expansion <1ha, DV4: Expansion <5ha, DV5: Expansion <10ha, DV6: Expansion in Switzerland, DV7: Expanion in neighbourhood. Statistical significance was derived from linear least squares regression and two-sided tests. Respective standard errors are reported in brackets and significant effects are indicated with: *** for $p < 0.001$, ** for $p < 0.01$, * for $p < 0.05$ and $\cdot$ for $p < 0.1$.

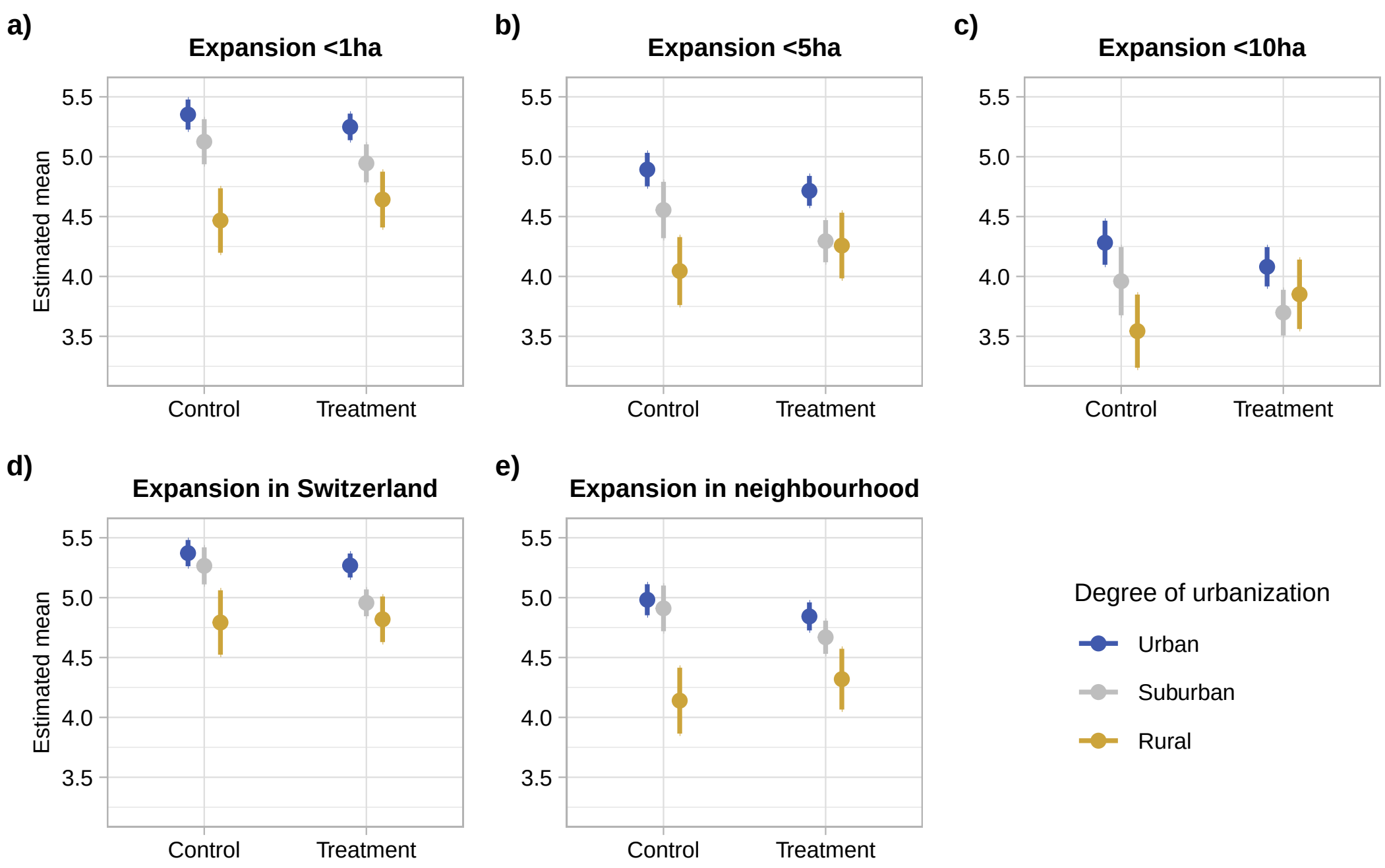

SI Figure 8: Estimation of attitudes towards the expansion of agrivoltaics by treatment group across levels of urbanization using marginal means. For all panels, data points represent the mean support estimates for all respondents (n = 2145) living in an urban (blue, n = 818), suburban (grey, n = 807), or rural (yellow, n = 520) environment in both the control (n = 707) and the information treatment groups (n = 1438) based on linear regressions and two-sided tests, with error bars indicating 95% confidence intervals.

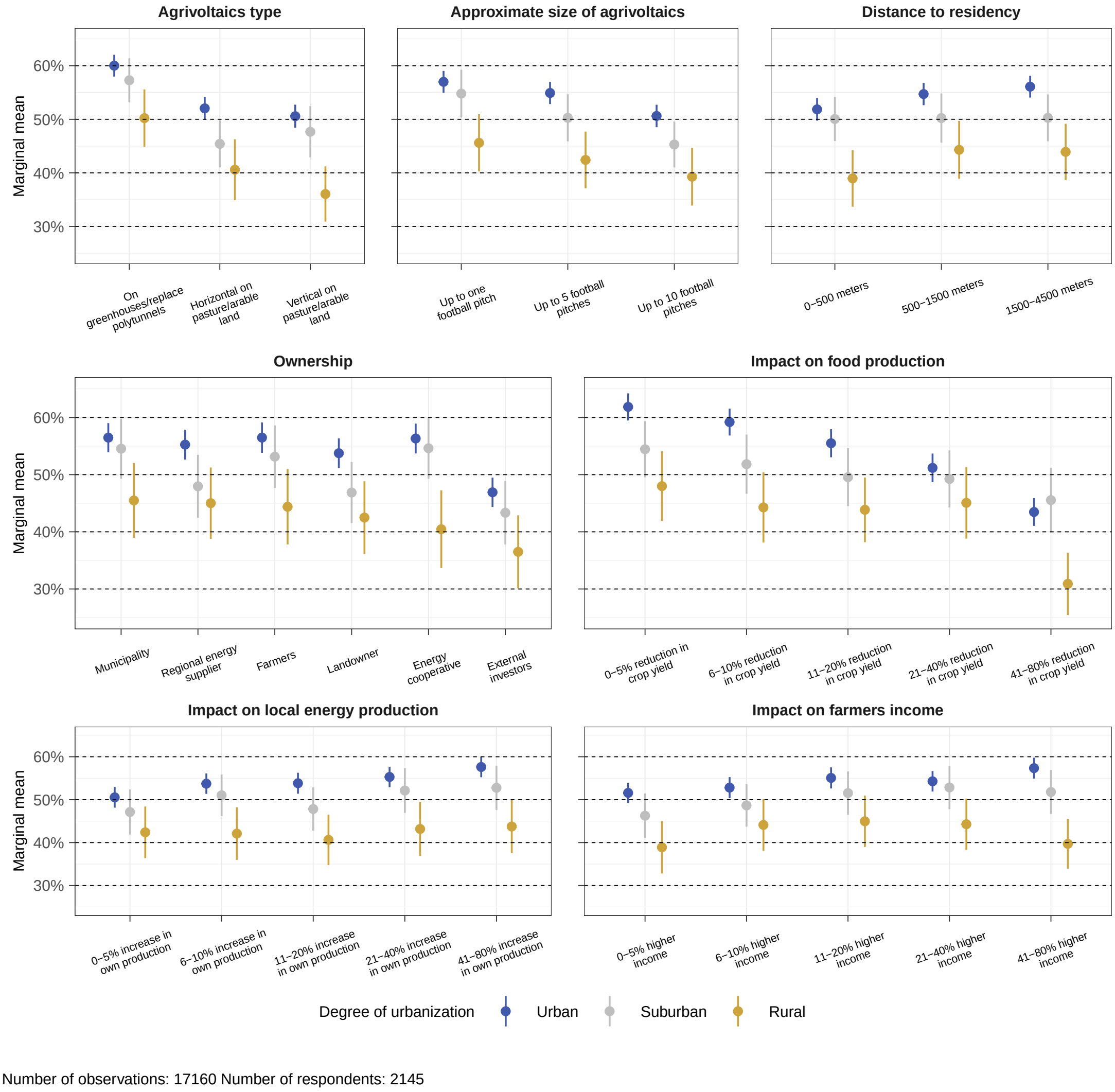

SI Figure 9: The figure displays the marginal means for clear support — defined as a support rating greater than 5 on a 7-point Likert scale — for differently designed agrivoltaics projects among residents in urban (blue, n = 1545), suburban (grey, n = 345), and rural (yellow, n = 255) environments in the overall sample (n = 2145). Here, the marginal means represent the predicted probability that a respondent from a particular subgroup expresses clear support for an agrivoltaics profile containing a given attribute level. Data points indicate the estimated marginal means for clear support (ranging between 0%-100%) for an agrivoltaics project with the respective design attribute. Vertical lines represent cluster-robust 95% confidence intervals derived from linear least squares regression and two-sided tests. Detailed results can be found in SI Table 24.

SI Table 24: The table displays the marginal means displayed in SI-Figure 9. The total N is 17'160 observations, i.e., 4 choice rounds between each 2 agrivoltaics design options for 2145 respondents (see further details in Methods)

Feature	Level	Degree of urbanization	Marginal mean	SE	Z-Value	p-Value	Significance
Agrivoltaics type	On greenhouses/replace polytunnels	Urban	0.600	0.010	57.836	<0.001	***
		Suburban	0.573	0.021	27.361	<0.001	***
		Rural	0.502	0.027	18.352	<0.001	***
	Horizontal on pasture/arable land	Urban	0.520	0.011	48.302	<0.001	***
		Suburban	0.454	0.022	20.287	<0.001	***
		Rural	0.406	0.029	13.987	<0.001	***
	Vertical on pasture/arable land	Urban	0.506	0.011	45.989	<0.001	***
		Suburban	0.477	0.024	19.483	<0.001	***
		Rural	0.360	0.026	13.701	<0.001	***
Approximate size of agrivoltaics	Up to one football pitch	Urban	0.570	0.010	54.738	<0.001	***
		Suburban	0.548	0.023	24.028	<0.001	***
		Rural	0.456	0.027	16.733	<0.001	***
	Up to 5 football pitches	Urban	0.549	0.011	51.990	<0.001	***
		Suburban	0.503	0.022	22.406	<0.001	***
		Rural	0.424	0.027	15.699	<0.001	***
	Up to 10 football pitches	Urban	0.506	0.011	47.337	<0.001	***
		Suburban	0.453	0.022	20.719	<0.001	***
		Rural	0.393	0.027	14.307	<0.001	***
Distance to residency	0-500 meters	Urban	0.519	0.011	48.025	<0.001	***

		Suburban	0.501	0.021	23.835	<0.001	***
		Rural	0.390	0.027	14.455	<0.001	***
	500-1500 meters	Urban	0.547	0.011	51.675	<0.001	***
		Suburban	0.502	0.023	21.419	<0.001	***
		Rural	0.443	0.028	16.046	<0.001	***
	1500-4500 meters	Urban	0.561	0.010	54.215	<0.001	***
		Suburban	0.503	0.022	22.523	<0.001	***
		Rural	0.439	0.027	16.390	<0.001	***
Ownership	Municipality	Urban	0.565	0.013	43.671	<0.001	***
		Suburban	0.545	0.027	20.383	<0.001	***
		Rural	0.455	0.033	13.623	<0.001	***
	Regional energy supplier	Urban	0.553	0.013	41.666	<0.001	***
		Suburban	0.480	0.028	17.060	<0.001	***
		Rural	0.450	0.032	14.144	<0.001	***
	Farmers	Urban	0.565	0.014	41.614	<0.001	***
		Suburban	0.531	0.028	19.069	<0.001	***
		Rural	0.444	0.034	13.195	<0.001	***
	Landowner	Urban	0.538	0.013	40.510	<0.001	***
		Suburban	0.469	0.027	17.220	<0.001	***
		Rural	0.425	0.032	13.137	<0.001	***
	Energy cooperative	Urban	0.563	0.013	42.154	<0.001	***
		Suburban	0.546	0.027	20.013	<0.001	***
		Rural	0.405	0.035	11.671	<0.001	***
	External investors	Urban	0.469	0.013	35.812	<0.001	***
		Suburban	0.433	0.028	15.278	<0.001	***

		Rural	0.365	0.033	11.204	<0.001	***
Impact on food production	0-5% reduction in crop yield	Urban	0.619	0.012	51.379	<0.001	***
		Suburban	0.544	0.025	21.705	<0.001	***
		Rural	0.480	0.031	15.454	<0.001	***
	6-10% reduction in crop yield	Urban	0.592	0.012	49.311	<0.001	***
		Suburban	0.518	0.026	19.614	<0.001	***
		Rural	0.443	0.031	14.113	<0.001	***
	11-20% reduction in crop yield	Urban	0.555	0.013	44.214	<0.001	***
		Suburban	0.496	0.026	19.169	<0.001	***
		Rural	0.438	0.029	15.170	<0.001	***
	21-40% reduction in crop yield	Urban	0.512	0.013	40.038	<0.001	***
		Suburban	0.492	0.025	19.332	<0.001	***
		Rural	0.451	0.032	14.117	<0.001	***
	41-80% reduction in crop yield	Urban	0.435	0.012	35.085	<0.001	***
		Suburban	0.455	0.029	15.853	<0.001	***
		Rural	0.309	0.028	11.114	<0.001	***
Impact on local energy production	0-5% increase in own production	Urban	0.505	0.012	41.125	<0.001	***
		Suburban	0.471	0.027	17.587	<0.001	***
		Rural	0.424	0.031	13.819	<0.001	***
	6-10% increase in own production	Urban	0.537	0.012	44.354	<0.001	***
		Suburban	0.510	0.025	20.557	<0.001	***

		Rural	0.421	0.031	13.504	<0.001	***
	11-20% increase in own production	Urban	0.538	0.012	43.242	<0.001	***
		Suburban	0.478	0.026	18.574	<0.001	***
		Rural	0.406	0.030	13.566	<0.001	***
	21-40% increase in own production	Urban	0.553	0.012	45.353	<0.001	***
		Suburban	0.521	0.027	19.627	<0.001	***
		Rural	0.432	0.032	13.427	<0.001	***
	41-80% increase in own production	Urban	0.576	0.012	47.278	<0.001	***
		Suburban	0.528	0.026	20.062	<0.001	***
		Rural	0.437	0.031	13.891	<0.001	***
Impact on farmers income	0-5% higher income	Urban	0.516	0.012	42.889	<0.001	***
		Suburban	0.463	0.026	17.517	<0.001	***
		Rural	0.389	0.031	12.503	<0.001	***
	6-10% higher income	Urban	0.528	0.012	42.263	<0.001	***
		Suburban	0.487	0.025	19.234	<0.001	***
		Rural	0.441	0.031	14.426	<0.001	***
	11-20% higher income	Urban	0.551	0.013	43.860	<0.001	***
		Suburban	0.515	0.026	19.968	<0.001	***
		Rural	0.450	0.031	14.715	<0.001	***
	21-40% higher income	Urban	0.543	0.012	44.681	<0.001	***
		Suburban	0.528	0.026	20.527	<0.001	***
		Rural	0.443	0.030	14.544	<0.001	***
	41-80% higher income	Urban	0.573	0.012	46.272	<0.001	***

Suburban	0.518	0.026	19.756	<0.001	***
Rural	0.397	0.030	13.438	<0.001	***

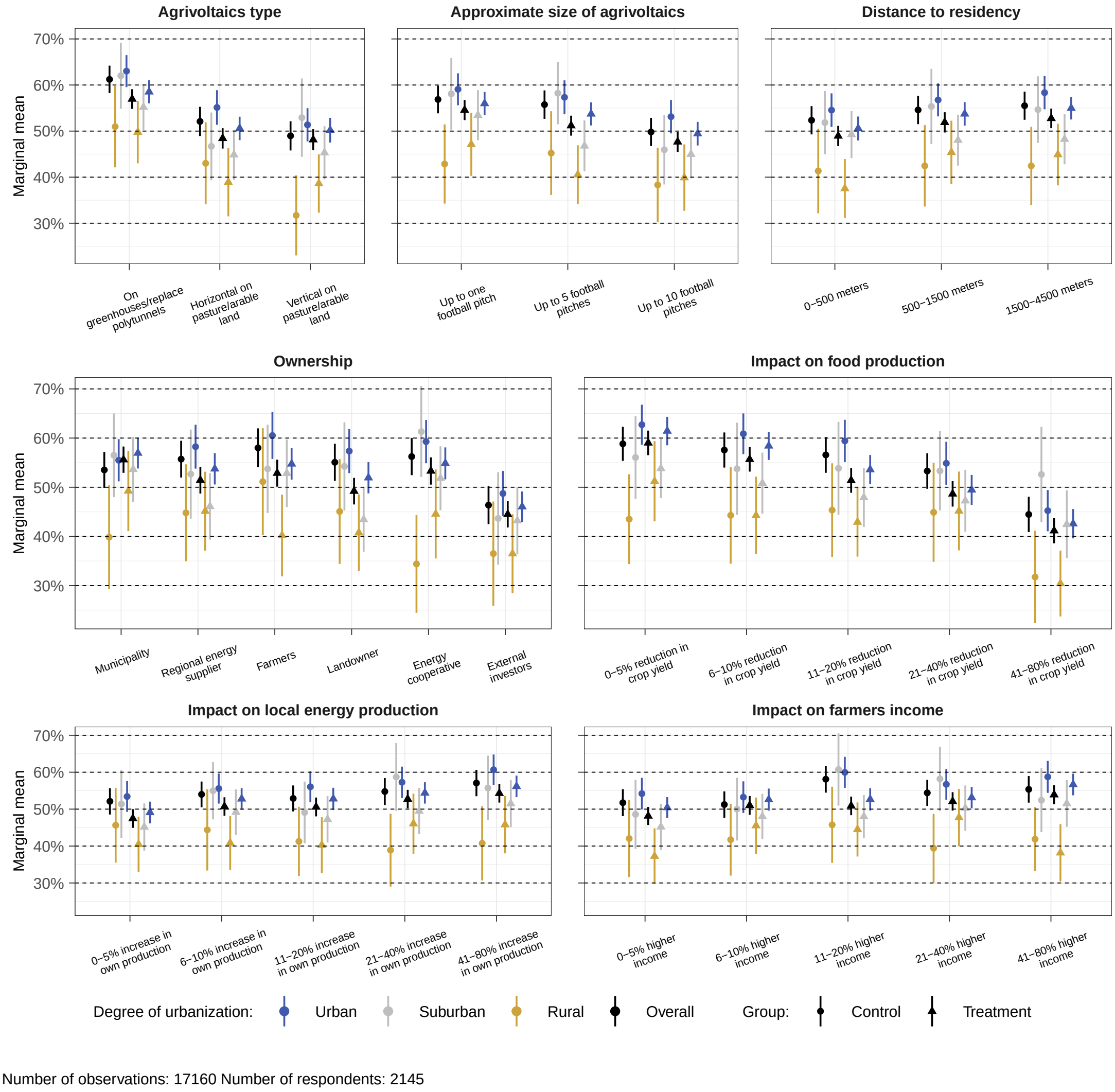

SI Figure 10: The figure displays the marginal means for clear support — defined as a support rating greater than 5 on a 7-point Likert scale — for differently designed agrivoltaics projects. Results are shown both for control (circles, n = 707) and treatment groups (triangles, n = 1438) across urban (blue, n = 1545), suburban (grey, n = 345), and rural (yellow, n = 255) environments, as well as for the overall control and treatment group, irrespective of the degree of urbanization (black). Here, the marginal means represent the predicted probability that a respondent from a particular subgroup expresses clear support for an agrivoltaics profile containing a given attribute level. Data points indicate the estimated marginal means for clear support (ranging between 0%-100%) for an agrivoltaics project with the respective design attribute. Vertical lines represent cluster-robust 95% confidence intervals derived from linear least squares regression and two-sided tests. Detailed results can be found in SI Table 25.

SI Table 25: The table displays the marginal means displayed in SI-Figure 10. The total N is 17'160 observations, i.e., 4 choice rounds between each 2 agrivoltaics design options for 2145 respondents (see further details in Methods)

Feature	Level	Group	Degree of urbanization	Marginal mean	SE	Z-Value	p-Value	Significance
Agrivoltaics type	On greenhouses/replace polytunnels	Control	Overall	0.612	0.015	40.313	<0.001	***
			Rural	0.510	0.045	11.304	<0.001	***
			Suburban	0.620	0.036	17.020	<0.001	***
			Urban	0.630	0.018	35.570	<0.001	***
		Treatment	Overall	0.570	0.011	52.381	<0.001	***
			Rural	0.498	0.035	14.407	<0.001	***
			Suburban	0.552	0.026	21.546	<0.001	***
			Urban	0.585	0.013	45.895	<0.001	***
	Horizontal on pasture/arable land	Control	Overall	0.521	0.016	32.274	<0.001	***
			Rural	0.430	0.045	9.476	<0.001	***
			Suburban	0.467	0.037	12.468	<0.001	***
			Urban	0.551	0.019	28.786	<0.001	***
		Treatment	Overall	0.484	0.011	42.898	<0.001	***
			Rural	0.389	0.038	10.289	<0.001	***
			Suburban	0.449	0.028	16.157	<0.001	***
			Urban	0.506	0.013	38.912	<0.001	***
	Vertical on pasture/arable land	Control	Overall	0.490	0.016	30.290	<0.001	***
			Rural	0.317	0.044	7.149	<0.001	***
			Suburban	0.529	0.043	12.214	<0.001	***
			Urban	0.514	0.018	27.936	<0.001	***
		Treatment	Overall	0.481	0.012	41.394	<0.001	***
			Rural	0.386	0.032	11.970	<0.001	***

			Suburban	0.453	0.029	15.399	<0.001	***
			Urban	0.502	0.014	36.697	<0.001	***
Approximate size of agrivoltaics	Up to one football pitch	Control	Overall	0.569	0.015	37.103	<0.001	***
			Rural	0.429	0.044	9.772	<0.001	***
			Suburban	0.581	0.040	14.632	<0.001	***
			Urban	0.591	0.018	33.334	<0.001	***
		Treatment	Overall	0.546	0.011	49.135	<0.001	***
			Rural	0.471	0.035	13.528	<0.001	***
			Suburban	0.535	0.028	19.250	<0.001	***
			Urban	0.560	0.013	43.627	<0.001	***
	Up to 5 football pitches	Control	Overall	0.557	0.016	35.384	<0.001	***
			Rural	0.452	0.046	9.757	<0.001	***
			Suburban	0.582	0.034	16.913	<0.001	***
			Urban	0.573	0.019	30.534	<0.001	***
		Treatment	Overall	0.512	0.011	46.308	<0.001	***
			Rural	0.405	0.033	12.459	<0.001	***
			Suburban	0.468	0.028	16.607	<0.001	***
			Urban	0.537	0.013	42.112	<0.001	***
	Up to 10 football pitches	Control	Overall	0.498	0.016	31.977	<0.001	***
			Rural	0.383	0.041	9.353	<0.001	***
			Suburban	0.459	0.038	11.956	<0.001	***
			Urban	0.531	0.019	28.692	<0.001	***
		Treatment	Overall	0.477	0.011	42.425	<0.001	***
			Rural	0.399	0.037	10.852	<0.001	***
			Suburban	0.450	0.027	16.876	<0.001	***

			Urban	0.494	0.013	37.791	<0.001	***
Distance to residency	0-500 meters	Control	Overall	0.524	0.016	33.433	<0.001	***
			Rural	0.413	0.047	8.829	<0.001	***
			Suburban	0.519	0.035	14.846	<0.001	***
			Urban	0.545	0.019	29.357	<0.001	***
		Treatment	Overall	0.489	0.011	43.654	<0.001	***
			Rural	0.375	0.033	11.488	<0.001	***
			Suburban	0.493	0.026	18.891	<0.001	***
			Urban	0.506	0.013	38.123	<0.001	***
	500-1500 meters	Control	Overall	0.546	0.016	34.661	<0.001	***
			Rural	0.425	0.045	9.423	<0.001	***
			Suburban	0.553	0.042	13.291	<0.001	***
			Urban	0.568	0.018	31.117	<0.001	***
		Treatment	Overall	0.519	0.011	46.269	<0.001	***
			Rural	0.454	0.035	12.948	<0.001	***
			Suburban	0.480	0.028	17.034	<0.001	***
			Urban	0.537	0.013	41.377	<0.001	***
	1500-4500 meters	Control	Overall	0.555	0.016	35.557	<0.001	***
			Rural	0.424	0.043	9.810	<0.001	***
			Suburban	0.547	0.037	14.845	<0.001	***
			Urban	0.583	0.018	31.631	<0.001	***
		Treatment	Overall	0.528	0.011	48.611	<0.001	***
			Rural	0.449	0.034	13.158	<0.001	***
			Suburban	0.482	0.028	17.368	<0.001	***
			Urban	0.550	0.012	44.060	<0.001	***

Ownership	Municipality	Control	Overall	0.535	0.019	28.794	<0.001	***
			Rural	0.398	0.054	7.415	<0.001	***
			Suburban	0.565	0.043	13.018	<0.001	***
			Urban	0.555	0.022	25.362	<0.001	***
		Treatment	Overall	0.556	0.014	40.713	<0.001	***
			Rural	0.492	0.042	11.810	<0.001	***
			Suburban	0.536	0.034	15.919	<0.001	***
			Urban	0.570	0.016	35.607	<0.001	***
	Regional energy supplier	Control	Overall	0.557	0.019	29.221	<0.001	***
			Rural	0.448	0.050	8.901	<0.001	***
			Suburban	0.527	0.046	11.401	<0.001	***
			Urban	0.582	0.023	25.575	<0.001	***
		Treatment	Overall	0.514	0.014	37.025	<0.001	***
			Rural	0.451	0.041	11.018	<0.001	***
			Suburban	0.461	0.034	13.368	<0.001	***
			Urban	0.537	0.016	33.076	<0.001	***
	Farmers	Control	Overall	0.580	0.020	28.745	<0.001	***
			Rural	0.511	0.055	9.214	<0.001	***
			Suburban	0.537	0.046	11.728	<0.001	***
			Urban	0.605	0.024	24.880	<0.001	***
		Treatment	Overall	0.529	0.014	37.733	<0.001	***
			Rural	0.402	0.042	9.474	<0.001	***
			Suburban	0.528	0.035	15.094	<0.001	***
			Urban	0.547	0.016	33.568	<0.001	***
	Landowner	Control	Overall	0.551	0.019	28.616	<0.001	***

			Rural	0.451	0.054	8.283	<0.001	***
			Suburban	0.542	0.046	11.847	<0.001	***
			Urban	0.574	0.023	25.103	<0.001	***
		Treatment	Overall	0.492	0.014	35.677	<0.001	***
			Rural	0.408	0.040	10.264	<0.001	***
			Suburban	0.434	0.033	12.980	<0.001	***
			Urban	0.519	0.016	31.982	<0.001	***
	Energy cooperative	Control	Overall	0.562	0.019	29.177	<0.001	***
			Rural	0.344	0.051	6.782	<0.001	***
			Suburban	0.613	0.047	13.007	<0.001	***
			Urban	0.593	0.022	26.401	<0.001	***
		Treatment	Overall	0.533	0.014	37.754	<0.001	***
			Rural	0.446	0.046	9.653	<0.001	***
			Suburban	0.518	0.033	15.615	<0.001	***
			Urban	0.549	0.017	33.092	<0.001	***
	External investors	Control	Overall	0.464	0.020	23.501	<0.001	***
			Rural	0.365	0.054	6.749	<0.001	***
			Suburban	0.437	0.048	9.095	<0.001	***
			Urban	0.487	0.023	20.821	<0.001	***
		Treatment	Overall	0.445	0.014	32.684	<0.001	***
			Rural	0.365	0.041	8.916	<0.001	***
			Suburban	0.432	0.035	12.349	<0.001	***
			Urban	0.460	0.016	29.134	<0.001	***
Impact on food production	0-5% reduction in crop yield	Control	Overall	0.588	0.018	33.321	<0.001	***
			Rural	0.435	0.047	9.341	<0.001	***

		Suburban	0.561	0.043	13.038	<0.001	***
		Urban	0.627	0.021	30.213	<0.001	***
	Treatment	Overall	0.590	0.013	46.301	<0.001	***
		Rural	0.512	0.042	12.314	<0.001	***
		Suburban	0.538	0.031	17.562	<0.001	***
		Urban	0.614	0.015	41.576	<0.001	***
6-10% reduction in crop yield	Control	Overall	0.576	0.018	31.584	<0.001	***
		Rural	0.443	0.050	8.824	<0.001	***
		Suburban	0.538	0.048	11.246	<0.001	***
		Urban	0.609	0.021	28.812	<0.001	***
	Treatment	Overall	0.557	0.013	43.775	<0.001	***
		Rural	0.443	0.040	11.007	<0.001	***
		Suburban	0.508	0.032	16.073	<0.001	***
		Urban	0.584	0.015	40.044	<0.001	***
11-20% reduction in crop yield	Control	Overall	0.566	0.019	30.486	<0.001	***
		Rural	0.453	0.049	9.333	<0.001	***
		Suburban	0.538	0.048	11.148	<0.001	***
		Urban	0.594	0.022	27.078	<0.001	***
	Treatment	Overall	0.514	0.013	40.189	<0.001	***
		Rural	0.429	0.036	11.995	<0.001	***
		Suburban	0.479	0.031	15.618	<0.001	***
		Urban	0.536	0.015	35.220	<0.001	***
21-40% reduction in crop yield	Control	Overall	0.533	0.018	28.936	<0.001	***
		Rural	0.449	0.051	8.746	<0.001	***
		Suburban	0.533	0.041	12.971	<0.001	***

			Urban	0.549	0.022	24.559	<0.001	***
		Treatment	Overall	0.487	0.013	36.771	<0.001	***
			Rural	0.452	0.041	11.048	<0.001	***
			Suburban	0.473	0.032	14.720	<0.001	***
			Urban	0.495	0.016	31.822	<0.001	***
	41-80% reduction in crop yield	Control	Overall	0.445	0.018	24.269	<0.001	***
			Rural	0.318	0.048	6.619	<0.001	***
			Suburban	0.526	0.050	10.625	<0.001	***
			Urban	0.452	0.021	21.160	<0.001	***
		Treatment	Overall	0.412	0.013	31.552	<0.001	***
			Rural	0.304	0.034	8.902	<0.001	***
			Suburban	0.425	0.035	12.049	<0.001	***
			Urban	0.426	0.015	28.003	<0.001	***
Impact on local energy production	0-5% increase in own production	Control	Overall	0.521	0.018	28.681	<0.001	***
			Rural	0.456	0.052	8.828	<0.001	***
			Suburban	0.514	0.047	10.941	<0.001	***
			Urban	0.534	0.021	25.127	<0.001	***
		Treatment	Overall	0.475	0.013	36.842	<0.001	***
			Rural	0.405	0.038	10.627	<0.001	***
			Suburban	0.452	0.033	13.841	<0.001	***
			Urban	0.491	0.015	32.663	<0.001	***
	6-10% increase in own production	Control	Overall	0.540	0.018	30.395	<0.001	***
			Rural	0.444	0.056	7.899	<0.001	***
			Suburban	0.550	0.040	13.887	<0.001	***
			Urban	0.556	0.021	26.821	<0.001	***

	Treatment	Overall	0.507	0.013	39.790	<0.001	***
		Rural	0.409	0.037	10.960	<0.001	***
		Suburban	0.492	0.031	15.667	<0.001	***
		Urban	0.528	0.015	35.390	<0.001	***
11-20% increase in own production	Control	Overall	0.529	0.018	29.493	<0.001	***
		Rural	0.412	0.048	8.629	<0.001	***
		Suburban	0.491	0.043	11.517	<0.001	***
		Urban	0.560	0.021	26.211	<0.001	***
	Treatment	Overall	0.506	0.013	38.811	<0.001	***
		Rural	0.402	0.039	10.429	<0.001	***
		Suburban	0.472	0.032	14.706	<0.001	***
		Urban	0.528	0.015	34.562	<0.001	***
21-40% increase in own production	Control	Overall	0.548	0.019	29.553	<0.001	***
		Rural	0.389	0.050	7.725	<0.001	***
		Suburban	0.587	0.047	12.496	<0.001	***
		Urban	0.573	0.022	26.448	<0.001	***
	Treatment	Overall	0.527	0.013	41.372	<0.001	***
		Rural	0.461	0.042	11.094	<0.001	***
		Suburban	0.495	0.032	15.486	<0.001	***
		Urban	0.544	0.015	36.890	<0.001	***
41-80% increase in own production	Control	Overall	0.571	0.018	31.316	<0.001	***
		Rural	0.407	0.051	7.941	<0.001	***
		Suburban	0.557	0.045	12.524	<0.001	***
		Urban	0.607	0.021	28.935	<0.001	***
	Treatment	Overall	0.543	0.013	42.318	<0.001	***

			Rural	0.458	0.040	11.561	<0.001	***
			Suburban	0.515	0.032	15.889	<0.001	***
			Urban	0.562	0.015	37.701	<0.001	***
Impact on farmers income	0-5% higher income	Control	Overall	0.517	0.019	27.749	<0.001	***
			Rural	0.420	0.053	7.901	<0.001	***
			Suburban	0.486	0.048	10.165	<0.001	***
			Urban	0.542	0.022	24.853	<0.001	***
		Treatment	Overall	0.481	0.012	38.608	<0.001	***
			Rural	0.373	0.038	9.721	<0.001	***
			Suburban	0.452	0.032	14.223	<0.001	***
			Urban	0.504	0.014	34.997	<0.001	***
	6-10% higher income	Control	Overall	0.512	0.018	28.116	<0.001	***
			Rural	0.417	0.050	8.409	<0.001	***
			Suburban	0.500	0.043	11.525	<0.001	***
			Urban	0.532	0.022	24.336	<0.001	***
		Treatment	Overall	0.510	0.013	39.462	<0.001	***
			Rural	0.455	0.039	11.724	<0.001	***
			Suburban	0.480	0.031	15.391	<0.001	***
			Urban	0.526	0.015	34.544	<0.001	***
	11-20% higher income	Control	Overall	0.581	0.019	31.295	<0.001	***
			Rural	0.458	0.053	8.695	<0.001	***
			Suburban	0.607	0.050	12.152	<0.001	***
			Urban	0.599	0.022	27.864	<0.001	***
		Treatment	Overall	0.509	0.013	39.533	<0.001	***
			Rural	0.445	0.037	11.895	<0.001	***

		Suburban	0.480	0.030	16.119	<0.001	***
		Urban	0.527	0.015	34.177	<0.001	***
21-40% higher income	Control	Overall	0.544	0.018	30.180	<0.001	***
		Rural	0.394	0.047	8.305	<0.001	***
		Suburban	0.582	0.045	13.063	<0.001	***
		Urban	0.567	0.021	26.621	<0.001	***
	Treatment	Overall	0.521	0.013	41.121	<0.001	***
		Rural	0.477	0.039	12.089	<0.001	***
		Suburban	0.503	0.031	16.035	<0.001	***
		Urban	0.531	0.015	35.940	<0.001	***
41-80% higher income	Control	Overall	0.554	0.018	30.142	<0.001	***
		Rural	0.419	0.044	9.448	<0.001	***
		Suburban	0.524	0.044	11.873	<0.001	***
		Urban	0.587	0.022	26.594	<0.001	***
	Treatment	Overall	0.539	0.013	41.363	<0.001	***
		Rural	0.382	0.040	9.662	<0.001	***
		Suburban	0.515	0.032	15.978	<0.001	***
		Urban	0.567	0.015	37.844	<0.001	***

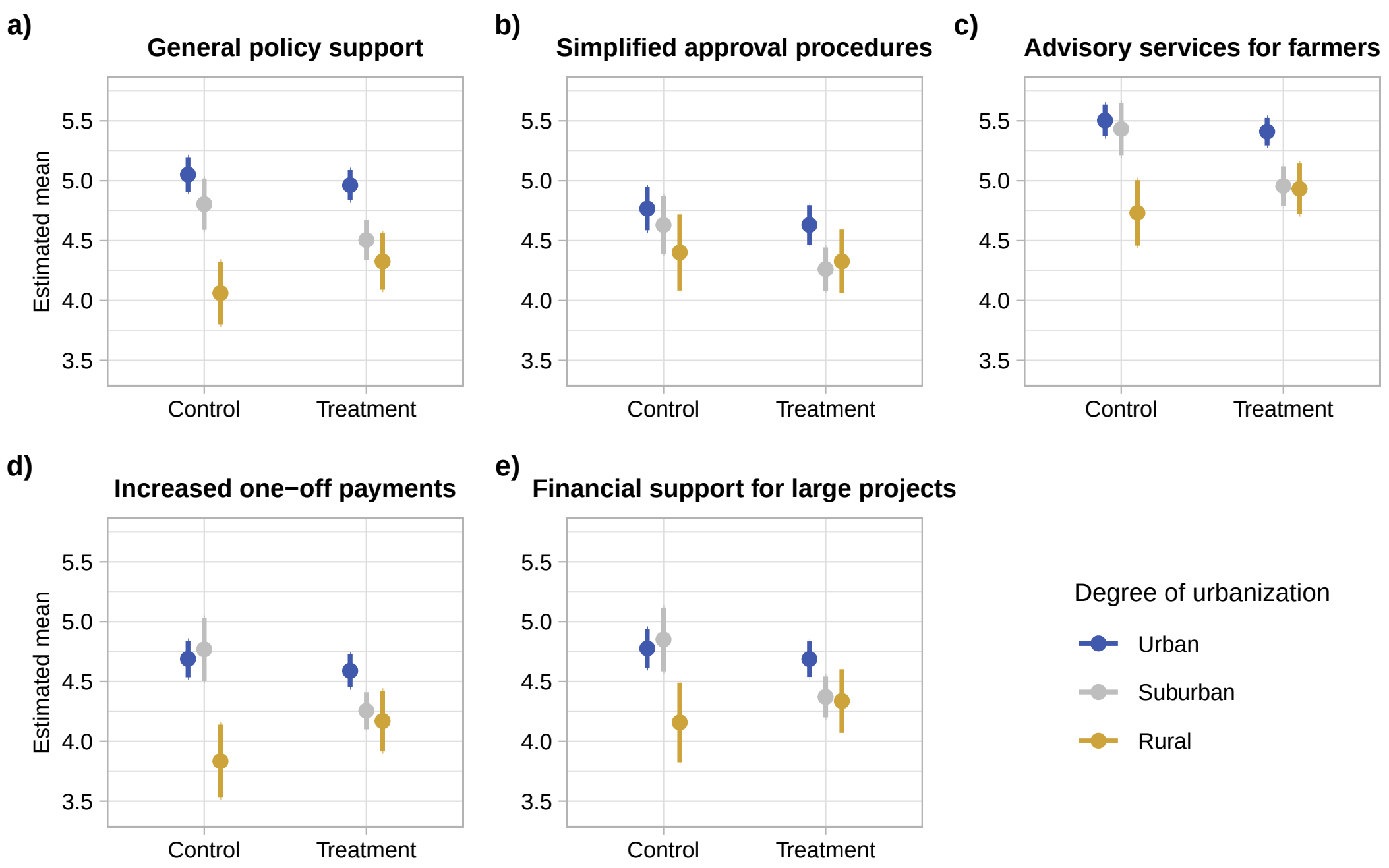

SI Figure 11: Estimation of policy support by treatment group across levels of urbanization using marginal means. For all panels, data points represent the mean support estimates for all respondents (n = 2145) living in an urban (blue, n = 818), suburban (grey, n = 807), or rural (yellow, n = 520) environment in both the control (n = 707) and the information treatment groups (n = 1438) based on linear regressions and two-sided tests, with error bars indicating 95% confidence intervals.

SI Table 26

	DV8, Urban	DV8, Suburban	DV8, Rural	DV9, Urban	DV9, Suburban	DV9, Rural	DV10, Urban	DV10, Suburban	DV10, Rural	DV11, Urban	DV11, Suburban	DV11, Rural	DV12, Urban	DV12, Suburban	DV12, Rural
Intercept	−0.085	0.471	−1.805**	0.737**	0.667	−0.922	1.716***	2.057***	−0.463	1.529***	2.162***	0.116	1.208***	2.355***	0.719
	(0.260)	(0.618)	(0.593)	(0.277)	(0.730)	(0.758)	(0.256)	(0.590)	(0.706)	(0.272)	(0.585)	(0.776)	(0.275)	(0.668)	(0.723)
Treatment	−0.088	−0.300*	0.265	−0.137*	−0.368*	−0.074	−0.093	−0.476***	0.200	−0.098	−0.512***	0.335·	−0.089	−0.480**	0.179
	(0.062)	(0.136)	(0.162)	(0.068)	(0.153)	(0.179)	(0.059)	(0.137)	(0.169)	(0.066)	(0.153)	(0.184)	(0.066)	(0.158)	(0.194)
Realistic agrivoltaics potential															
Potential in circle 1	−0.007	−0.188	−0.054	−0.047	−0.292·	−0.176	0.126	−0.001	−0.145	0.051	−0.311*	−0.106	0.079	−0.222	0.099
	(0.091)	(0.155)	(0.166)	(0.108)	(0.163)	(0.175)	(0.087)	(0.142)	(0.164)	(0.093)	(0.144)	(0.178)	(0.098)	(0.161)	(0.184)
Potential in circle 2	−0.058	0.053	0.289	−0.079	0.086	0.326	−0.092	0.109	0.498·	0.013	0.362·	0.109	0.021	0.191	0.257
	(0.120)	(0.189)	(0.530)	(0.155)	(0.209)	(0.350)	(0.118)	(0.180)	(0.288)	(0.138)	(0.204)	(0.335)	(0.141)	(0.195)	(0.392)
Potential in circle 3	0.124	−0.112	−0.071	0.030	−0.181	0.014	0.096	−0.141	−0.066	0.128	−0.124	0.290	−0.007	−0.220	−0.066
	(0.111)	(0.195)	(0.513)	(0.139)	(0.207)	(0.378)	(0.119)	(0.173)	(0.297)	(0.134)	(0.197)	(0.361)	(0.137)	(0.198)	(0.420)
Controls															
Age	0.004*	0.011*	0.005	0.001	0.010*	0.004	−0.000	0.003	0.007	0.003	0.005	0.009	−0.004*	−0.007	−0.003
	(0.002)	(0.005)	(0.005)	(0.002)	(0.005)	(0.006)	(0.002)	(0.004)	(0.005)	(0.002)	(0.005)	(0.006)	(0.002)	(0.005)	(0.006)
Gender: Male	0.124*	−0.031	−0.008	0.268***	0.249	0.313·	−0.014	0.017	0.217	0.019	−0.021	0.000	−0.017	−0.023	−0.135
	(0.062)	(0.143)	(0.172)	(0.067)	(0.157)	(0.181)	(0.057)	(0.143)	(0.158)	(0.063)	(0.140)	(0.179)	(0.065)	(0.151)	(0.179)
Environmental score	0.206***	0.189*	0.313***	0.115**	0.158·	0.192*	0.118***	0.132·	0.159·	0.098**	0.100	0.079	0.217***	0.082	0.126
	(0.035)	(0.076)	(0.077)	(0.035)	(0.087)	(0.092)	(0.032)	(0.074)	(0.086)	(0.033)	(0.071)	(0.095)	(0.035)	(0.082)	(0.089)
Familiarity with agrivoltaics	0.059**	0.055	0.062	0.074***	0.082·	−0.017	0.031·	0.060	−0.007	0.030	0.061	0.010	0.042*	0.081·	0.053
	(0.019)	(0.044)	(0.053)	(0.020)	(0.049)	(0.057)	(0.017)	(0.041)	(0.047)	(0.020)	(0.040)	(0.060)	(0.020)	(0.045)	(0.058)
Prior agrivoltaics preference	0.684***	0.635***	0.785***	0.562***	0.527***	0.735***	0.572***	0.539***	0.757***	0.465***	0.434***	0.551***	0.478***	0.542***	0.613***
	(0.025)	(0.060)	(0.045)	(0.026)	(0.059)	(0.053)	(0.027)	(0.053)	(0.051)	(0.027)	(0.054)	(0.056)	(0.028)	(0.058)	(0.057)
Political orientation	−0.062***	−0.136***	−0.042	−0.029	−0.085·	0.043	−0.047**	−0.120**	−0.003	−0.070***	−0.133***	−0.016	−0.057**	−0.135**	−0.072·
	(0.018)	(0.039)	(0.037)	(0.018)	(0.050)	(0.042)	(0.015)	(0.039)	(0.038)	(0.017)	(0.040)	(0.042)	(0.018)	(0.046)	(0.043)
Observations	1545	345	255	1545	345	255	1545	345	255	1545	345	255	1545	345	255
R^2	0.456	0.450	0.606	0.313	0.324	0.475	0.384	0.383	0.554	0.262	0.323	0.356	0.299	0.360	0.402
Adj. R^2	0.453	0.433	0.590	0.309	0.304	0.454	0.380	0.365	0.536	0.257	0.302	0.330	0.295	0.341	0.378
RMSE	1.169	1.245	1.224	1.269	1.366	1.365	1.091	1.229	1.230	1.221	1.232	1.374	1.239	1.316	1.408

Table 26: DV8: General policy support , DV9: Simplified approval procedures, DV10: Advisory services for farmers, DV11: Increased one-off payments, DV12: Financial support for large projects. Statistical significance was derived from linear least squares regression and two-sided tests. Respective standard errors are reported in brackets and significant effects are indicated with: *** for $p < 0.001$, ** for $p < 0.01$, * for $p < 0.05$ and · for $p < 0.1$.

SI Table 27

	CC1, Urban	CC1, Suburban	CC1, Rural	CC2, Urban	CC2, Suburban	CC2, Rural	CC3, Urban	CC3, Suburban	CC3, Rural
Intercept	0.061	0.269	−0.018	0.088	0.218	−0.123	0.250^{*}	0.128	−0.079
	(0.063)	(0.198)	(0.180)	(0.082)	(0.242)	(0.201)	(0.099)	(0.235)	(0.246)
Treatment	-0.036^{*}	−0.056	−0.071	-0.093^{***}	−0.134	$-0.107^{\cdot}$	-0.293^{***}	-0.283^{**}	-0.176^{*}
	(0.015)	(0.065)	(0.051)	(0.022)	(0.086)	(0.064)	(0.028)	(0.089)	(0.082)
Realistic agrivoltaics potential									
Potential in circle 1	$0.089^{\cdot}$	0.076	−0.011	0.194^{**}	0.047	0.100	−0.020	−0.149	−0.020
	(0.052)	(0.083)	(0.060)	(0.070)	(0.098)	(0.085)	(0.076)	(0.109)	(0.099)
Potential in circle 2	0.049	0.068	0.002	0.111	0.143	0.229	0.126	0.030	$0.456^{\cdot}$
	(0.058)	(0.129)	(0.350)	(0.084)	(0.134)	(0.333)	(0.098)	(0.136)	(0.262)
Potential in circle 3	−0.017	0.026	0.050	0.098	−0.078	−0.112	0.128	$0.230^{\cdot}$	−0.293
	(0.037)	(0.124)	(0.367)	(0.079)	(0.128)	(0.356)	(0.089)	(0.131)	(0.290)
Controls									
Age	-0.001^{**}	-0.003^{*}	0.000	-0.002^{**}	−0.002	-0.003^{*}	−0.001	−0.002	−0.002
	(0.000)	(0.001)	(0.001)	(0.001)	(0.002)	(0.001)	(0.001)	(0.002)	(0.002)
Gender: Male	0.017	−0.021	0.041	0.026	0.100^{*}	$0.085^{\cdot}$	0.031	0.150^{**}	0.149^{**}
	(0.014)	(0.044)	(0.043)	(0.018)	(0.050)	(0.048)	(0.024)	(0.052)	(0.057)
Environmental score	−0.006	$-0.040^{\cdot}$	0.030	−0.015	−0.010	$0.042^{\cdot}$	0.002	0.037	$0.054^{\cdot}$
	(0.008)	(0.023)	(0.022)	(0.010)	(0.026)	(0.022)	(0.012)	(0.026)	(0.029)
Familiarity with agrivoltaics	$0.008^{\cdot}$	0.007	−0.002	0.011^{*}	−0.007	0.027	0.014^{*}	−0.007	0.011
	(0.004)	(0.014)	(0.014)	(0.006)	(0.015)	(0.016)	(0.007)	(0.015)	(0.018)
Prior agrivoltaics preference	0.014^{**}	0.052^{**}	−0.004	0.029^{***}	0.055^{**}	0.036^{*}	0.038^{***}	0.064^{**}	0.041^{*}
	(0.005)	(0.016)	(0.014)	(0.007)	(0.020)	(0.015)	(0.008)	(0.020)	(0.018)
Political orientation	−0.001	−0.019	−0.005	0.007	−0.021	−0.012	$0.012^{\cdot}$	−0.013	−0.007
	(0.004)	(0.013)	(0.011)	(0.005)	(0.015)	(0.011)	(0.006)	(0.016)	(0.015)
Interactions									
NIMBY × Pot. in Circle1	0.291^{***}	0.208^{*}	0.214^{*}	−0.050	0.114	0.034	0.056	0.205	0.068
	(0.066)	(0.099)	(0.086)	(0.080)	(0.118)	(0.107)	(0.087)	(0.130)	(0.124)
NIMBY × Pot. in Circle2	−0.085	−0.029	0.029	0.181	0.068	0.148	−0.029	0.153	−0.130
	(0.086)	(0.154)	(0.365)	(0.111)	(0.166)	(0.360)	(0.124)	(0.168)	(0.314)
NIMBY × Pot. in Circle3	0.009	−0.041	0.094	−0.117	0.047	0.019	0.309^{**}	−0.141	0.424
	(0.058)	(0.151)	(0.390)	(0.095)	(0.162)	(0.389)	(0.108)	(0.164)	(0.342)
Observations	1545	345	255	1545	345	255	1545	345	255
R^2	0.156	0.153	0.120	0.104	0.133	0.198	0.136	0.179	0.189
Adj. R^2	0.149	0.119	0.072	0.096	0.098	0.154	0.129	0.147	0.146
RMSE	0.267	0.391	0.328	0.351	0.448	0.376	0.454	0.462	0.443

Table 27: CC1: Knowledge of potential in circle 1 (0m-500m, binary), CC2: Knowledge of potential in circle 2 (500m-1500m, binary), CC3: Knowledge of potential in circle 3 (1500m-4500m, binary). Statistical significance was derived from linear least squares regression and two-sided tests. Respective standard errors are reported in brackets and significant effects are indicated with: *** for $p < 0.001$, ** for $p < 0.01$, * for $p < 0.05$ and $\cdot$ for $p < 0.1$.

SI Table 28

	DV1, Urban	DV1, Suburban	DV1, Rural	DV2, Urban	DV2, Suburban	DV2, Rural
Intercept	1.327***	2.026***	−0.755	1.393***	2.112***	0.199
	(0.224)	(0.541)	(0.675)	(0.225)	(0.469)	(0.649)
Treatment	−0.131*	−0.110	0.282	−0.052	−0.177	−0.051
	(0.059)	(0.156)	(0.203)	(0.057)	(0.162)	(0.198)
Realistic agrivoltaics potential						
Potential in circle 1	−0.189	−0.016	0.556*	0.134	−0.145	0.302
	(0.174)	(0.196)	(0.256)	(0.145)	(0.207)	(0.247)
Potential in circle 2	−0.199	−0.151	0.830**	−0.216	−0.185	0.709**
	(0.205)	(0.216)	(0.290)	(0.198)	(0.232)	(0.260)
Potential in circle 3	0.042	0.056	−0.901**	−0.071	0.108	−0.911***
	(0.156)	(0.204)	(0.317)	(0.136)	(0.227)	(0.273)
Controls						
Age	−0.003·	−0.002	0.007	−0.002	−0.001	0.001
	(0.002)	(0.004)	(0.005)	(0.002)	(0.003)	(0.005)
Gender: Male	0.009	−0.002	0.174	0.004	0.078	−0.009
	(0.055)	(0.115)	(0.151)	(0.051)	(0.100)	(0.146)
Environmental score	0.128***	0.037	0.217**	0.125***	0.071	0.176*
	(0.029)	(0.058)	(0.079)	(0.027)	(0.053)	(0.077)
Familiarity with agrivoltaics	0.035*	0.069*	0.031	0.029·	0.034	−0.008
	(0.017)	(0.033)	(0.049)	(0.016)	(0.029)	(0.049)
Prior agrivoltaics preference	0.571***	0.603***	0.670***	0.577***	0.599***	0.718***
	(0.024)	(0.042)	(0.049)	(0.024)	(0.038)	(0.048)
Political orientation	0.005	−0.105**	0.007	−0.004	−0.128***	−0.001
	(0.014)	(0.035)	(0.039)	(0.014)	(0.029)	(0.035)
Interactions						
NIMBY × Pot. in Circle1	0.118	−0.139	−0.690*	−0.129	0.003	−0.253
	(0.196)	(0.247)	(0.314)	(0.167)	(0.246)	(0.307)
NIMBY × Pot. in Circle2	0.331	0.126	−1.435*	0.140	0.341	−1.183*
	(0.256)	(0.292)	(0.604)	(0.247)	(0.278)	(0.581)
NIMBY × Pot. in Circle3	0.023	−0.037	1.480*	0.297	−0.208	1.426*
	(0.214)	(0.287)	(0.621)	(0.185)	(0.283)	(0.570)
Observations	1545	345	255	1545	345	255
R^2	0.394	0.486	0.541	0.431	0.543	0.582
Adj. R^2	0.389	0.466	0.517	0.427	0.525	0.559
RMSE	1.041	1.005	1.129	0.970	0.905	1.085

Table 28: DV1: Personal advantage from agrivoltaics (1-7 Likert scale), DV2: Advantage for Switzerland (1-7 Likert scale). Statistical significance was derived from linear least squares regression and two-sided tests. Respective standard errors are reported in brackets and significant effects are indicated with: *** for $p < 0.001$, ** for $p < 0.01$, * for $p < 0.05$ and · for $p < 0.1$.

SI Table 29

	DV3, Urban	DV3, Suburban	DV3, Rural	DV4, Urban	DV4, Suburban	DV4, Rural	DV5, Urban	DV5, Suburban	DV5, Rural	DV6, Urban	DV6, Suburban	DV6, Rural	DV7, Urban	DV7, Suburban	DV7, Rural
Intercept	1.432***	1.543**	0.084	0.772**	1.968**	−0.030	0.439	1.753*	−0.422	0.906***	1.257**	0.497	0.821***	0.699	−1.398*
	(0.232)	(0.593)	(0.657)	(0.250)	(0.629)	(0.658)	(0.301)	(0.708)	(0.802)	(0.198)	(0.444)	(0.632)	(0.228)	(0.493)	(0.693)
Treatment	−0.138*	−0.083	0.250	−0.179**	−0.207	0.221	−0.162*	−0.322	0.503*	−0.143**	−0.197	0.208	−0.164**	−0.103	0.369$^{\cdot}$
	(0.059)	(0.202)	(0.206)	(0.064)	(0.243)	(0.241)	(0.082)	(0.259)	(0.248)	(0.051)	(0.165)	(0.181)	(0.059)	(0.192)	(0.206)
Realistic agrivoltaics potential															
Potential in circle 1	−0.019	−0.182	0.187	0.100	−0.081	−0.194	0.097	−0.191	−0.025	−0.128	−0.112	0.408$^{\cdot}$	−0.121	−0.062	−0.013
	(0.186)	(0.212)	(0.266)	(0.172)	(0.275)	(0.295)	(0.195)	(0.315)	(0.340)	(0.148)	(0.174)	(0.237)	(0.149)	(0.222)	(0.277)
Potential in circle 2	−0.055	0.514$^{\cdot}$	0.441	−0.197	0.719$^{\cdot}$	1.372**	−0.132	0.477	1.572*	0.041	−0.002	−0.222	0.033	0.039	1.423***
	(0.200)	(0.293)	(0.624)	(0.200)	(0.378)	(0.473)	(0.265)	(0.467)	(0.707)	(0.138)	(0.202)	(0.677)	(0.201)	(0.316)	(0.425)
Potential in circle 3	−0.140	−0.297	−0.518	−0.110	−0.781*	−1.129*	−0.063	−0.761$^{\cdot}$	−1.094	−0.190	0.044	−0.023	−0.299	0.019	−1.285**
	(0.170)	(0.270)	(0.620)	(0.179)	(0.370)	(0.493)	(0.278)	(0.460)	(0.665)	(0.172)	(0.210)	(0.677)	(0.183)	(0.284)	(0.453)
Controls															
Age	0.000	0.005	0.005	−0.003	0.003	−0.003	−0.007**	0.004	−0.007	0.004*	0.008*	0.005	0.000	0.008$^{\cdot}$	0.010$^{\cdot}$
	(0.002)	(0.004)	(0.005)	(0.002)	(0.004)	(0.005)	(0.002)	(0.005)	(0.006)	(0.001)	(0.003)	(0.005)	(0.002)	(0.004)	(0.005)
Gender: Male	0.018	0.134	0.168	0.354***	0.248$^{\cdot}$	0.242	0.520***	0.356*	0.274	0.080$^{\cdot}$	0.092	−0.025	0.268***	0.098	0.160
	(0.052)	(0.131)	(0.153)	(0.059)	(0.145)	(0.176)	(0.073)	(0.160)	(0.196)	(0.047)	(0.100)	(0.137)	(0.053)	(0.122)	(0.170)
Environmental score	0.132***	0.191**	0.245**	0.135***	0.017	0.218*	0.143***	−0.071	0.211*	0.153***	0.129*	0.155*	0.158***	0.176**	0.323***
	(0.027)	(0.072)	(0.077)	(0.030)	(0.074)	(0.087)	(0.037)	(0.080)	(0.089)	(0.024)	(0.050)	(0.069)	(0.029)	(0.062)	(0.081)
Familiarity with agrivoltaics	0.032*	0.011	−0.062	0.040*	0.015	−0.054	0.055*	0.013	−0.048	0.051***	0.065*	−0.020	0.051**	0.069$^{\cdot}$	0.011
	(0.016)	(0.040)	(0.049)	(0.018)	(0.045)	(0.058)	(0.022)	(0.048)	(0.063)	(0.014)	(0.028)	(0.049)	(0.016)	(0.037)	(0.057)
Prior agrivoltaics preference	0.579***	0.525***	0.656***	0.646***	0.610***	0.667***	0.625***	0.612***	0.609***	0.608***	0.605***	0.674***	0.605***	0.582***	0.663***
	(0.023)	(0.055)	(0.051)	(0.024)	(0.055)	(0.050)	(0.027)	(0.054)	(0.055)	(0.022)	(0.040)	(0.054)	(0.022)	(0.049)	(0.053)
Political orientation	−0.037**	−0.116*	−0.071$^{\cdot}$	−0.076***	−0.203***	−0.040	−0.084***	−0.180***	−0.000	−0.032**	−0.111***	−0.050	−0.059***	−0.109**	−0.033
	(0.014)	(0.047)	(0.038)	(0.016)	(0.045)	(0.042)	(0.020)	(0.049)	(0.045)	(0.012)	(0.028)	(0.033)	(0.014)	(0.034)	(0.039)
Interactions															
NIMBY × Pot. in Circle1	0.118	0.155	−0.326	−0.109	0.133	−0.082	−0.212	0.020	−0.253	0.091	−0.141	−0.698*	0.029	−0.399	−0.369
	(0.207)	(0.289)	(0.314)	(0.202)	(0.353)	(0.355)	(0.233)	(0.390)	(0.406)	(0.168)	(0.218)	(0.280)	(0.178)	(0.285)	(0.357)
NIMBY × Pot. in Circle2	0.063	−0.490	−0.429	0.232	−0.569	−1.169*	0.015	−0.071	−1.507$^{\cdot}$	−0.016	0.178	0.247	−0.042	0.432	−1.604*
	(0.246)	(0.366)	(0.685)	(0.257)	(0.467)	(0.559)	(0.346)	(0.538)	(0.891)	(0.188)	(0.256)	(0.723)	(0.270)	(0.366)	(0.790)
NIMBY × Pot. in Circle3	0.194	0.063	0.909	−0.011	0.296	1.514*	−0.177	0.207	1.352	0.363$^{\cdot}$	−0.290	0.485	0.295	−0.277	1.779*
	(0.216)	(0.355)	(0.714)	(0.241)	(0.461)	(0.630)	(0.330)	(0.539)	(0.897)	(0.202)	(0.266)	(0.740)	(0.243)	(0.335)	(0.800)
Observations	1545	345	255	1545	345	255	1545	345	255	1545	345	255	1545	345	255
R^2	0.435	0.406	0.545	0.448	0.404	0.491	0.352	0.333	0.402	0.518	0.595	0.597	0.467	0.480	0.545
Adj. R^2	0.430	0.382	0.520	0.443	0.380	0.463	0.346	0.307	0.370	0.514	0.580	0.575	0.463	0.460	0.521
RMSE	0.994	1.142	1.184	1.117	1.293	1.315	1.387	1.430	1.428	0.891	0.849	1.033	1.014	1.071	1.229

Table 29: DV3: Expansion <1ha, DV4: Expansion <5ha, DV5: Expansion <10ha, DV6: Expansion in Switzerland, DV7: Expanion in neighbourhood. Statistical significance was derived from linear least squares regression and two-sided tests. Respective standard errors are reported in brackets and significant effects are indicated with: *** for $p < 0.001$, ** for $p < 0.01$, * for $p < 0.05$ and $\cdot$ for $p < 0.1$.

SI Table 30

	DV8, Urban	DV8, Suburban	DV8, Rural	DV9, Urban	DV9, Suburban	DV9, Rural	DV10, Urban	DV10, Suburban	DV10, Rural	DV11, Urban	DV11, Suburban	DV11, Rural	DV12, Urban	DV12, Suburban	DV12, Rural
Intercept	−0.060	0.310	−1.601**	0.739**	0.310	−0.935	1.721***	1.934**	−0.403	1.548***	2.062***	0.046	1.206***	2.335***	0.686
	(0.260)	(0.637)	(0.591)	(0.278)	(0.637)	(0.747)	(0.257)	(0.603)	(0.714)	(0.273)	(0.620)	(0.807)	(0.276)	(0.679)	(0.740)
Treatment	−0.144*	−0.143	0.266	−0.147*	−0.143	0.052	−0.113$^{\cdot}$	−0.349$^{\cdot}$	0.326	−0.122$^{\cdot}$	−0.442$^{\cdot}$	0.349	−0.099	−0.339	0.193
	(0.067)	(0.196)	(0.211)	(0.073)	(0.196)	(0.248)	(0.064)	(0.210)	(0.208)	(0.072)	(0.245)	(0.251)	(0.072)	(0.249)	(0.261)
Realistic agrivoltaics potential															
Potential in circle 1	−0.046	−0.173	−0.114	0.270	−0.173	0.008	0.190	0.112	−0.142	0.076	−0.116	−0.059	0.216	−0.070	0.195
	(0.195)	(0.223)	(0.289)	(0.203)	(0.223)	(0.331)	(0.188)	(0.238)	(0.339)	(0.186)	(0.284)	(0.359)	(0.197)	(0.290)	(0.362)
Potential in circle 2	−0.140	−0.091	1.560***	−0.265	−0.091	0.714	−0.243	0.005	1.562***	0.171	0.189	−0.279	−0.170	0.453	0.010
	(0.224)	(0.260)	(0.459)	(0.251)	(0.260)	(0.636)	(0.195)	(0.327)	(0.279)	(0.198)	(0.360)	(0.939)	(0.220)	(0.334)	(1.178)
Potential in circle 3	−0.181	0.326	−1.383**	−0.326	0.326	−0.477	−0.020	0.054	−0.923***	−0.213	−0.081	0.670	−0.110	−0.456	0.042
	(0.214)	(0.272)	(0.473)	(0.262)	(0.272)	(0.668)	(0.203)	(0.308)	(0.222)	(0.222)	(0.342)	(0.899)	(0.265)	(0.347)	(1.156)
Controls															
Age	0.004*	0.011*	0.005	0.001	0.011*	0.005	−0.000	0.004	0.007	0.003	0.005	0.009	−0.004*	−0.006	−0.003
	(0.002)	(0.005)	(0.005)	(0.002)	(0.005)	(0.006)	(0.002)	(0.004)	(0.005)	(0.002)	(0.005)	(0.006)	(0.002)	(0.005)	(0.006)
Gender: Male	0.127*	−0.028	0.019	0.272***	−0.028	0.341$^{\cdot}$	−0.013	0.016	0.242	0.023	−0.027	−0.005	−0.016	−0.026	−0.131
	(0.062)	(0.143)	(0.172)	(0.067)	(0.143)	(0.186)	(0.057)	(0.143)	(0.158)	(0.063)	(0.141)	(0.184)	(0.065)	(0.151)	(0.186)
Environmental score	0.207***	0.193*	0.296***	0.115**	0.193*	0.180$^{\cdot}$	0.119***	0.136$^{\cdot}$	0.145$^{\cdot}$	0.097**	0.107	0.083	0.218***	0.077	0.126
	(0.035)	(0.076)	(0.077)	(0.035)	(0.076)	(0.093)	(0.032)	(0.074)	(0.087)	(0.033)	(0.073)	(0.096)	(0.035)	(0.081)	(0.088)
Familiarity with agrivoltaics	0.059**	0.056	0.056	0.074***	0.056	−0.020	0.031$^{\cdot}$	0.059	−0.005	0.030	0.056	0.011	0.043*	0.080$^{\cdot}$	0.050
	(0.019)	(0.045)	(0.053)	(0.020)	(0.045)	(0.056)	(0.017)	(0.042)	(0.047)	(0.020)	(0.041)	(0.062)	(0.020)	(0.046)	(0.059)
Prior agrivoltaics preference	0.685***	0.640***	0.786***	0.563***	0.640***	0.739***	0.572***	0.542***	0.758***	0.466***	0.436***	0.552***	0.479***	0.535***	0.615***
	(0.025)	(0.060)	(0.045)	(0.026)	(0.060)	(0.053)	(0.027)	(0.053)	(0.051)	(0.027)	(0.053)	(0.056)	(0.028)	(0.058)	(0.057)
Political orientation	−0.062***	−0.141***	−0.060	−0.030$^{\cdot}$	−0.141***	0.036	−0.047**	−0.122**	−0.014	−0.070***	−0.130**	−0.011	−0.057**	−0.140**	−0.071
	(0.018)	(0.039)	(0.037)	(0.018)	(0.039)	(0.042)	(0.015)	(0.040)	(0.039)	(0.017)	(0.040)	(0.044)	(0.018)	(0.047)	(0.045)
Interactions															
NIMBY × Pot. in Circle1	0.064	−0.007	0.074	−0.417$^{\cdot}$	−0.007	−0.296	−0.083	−0.162	−0.035	−0.018	−0.294	−0.066	−0.183	−0.226	−0.142
	(0.222)	(0.297)	(0.360)	(0.239)	(0.297)	(0.398)	(0.212)	(0.295)	(0.387)	(0.216)	(0.331)	(0.413)	(0.228)	(0.350)	(0.419)
NIMBY × Pot. in Circle2	0.128	0.262	−1.853*	0.276	0.262	−0.591	0.234	0.181	−1.552***	−0.255	0.271	0.562	0.295	−0.404	0.349
	(0.263)	(0.376)	(0.805)	(0.318)	(0.376)	(0.769)	(0.246)	(0.389)	(0.422)	(0.270)	(0.438)	(1.006)	(0.287)	(0.413)	(1.228)
NIMBY × Pot. in Circle3	0.482$^{\cdot}$	−0.685$^{\cdot}$	1.958*	0.529$^{\cdot}$	−0.685$^{\cdot}$	0.755	0.176	−0.304	1.232**	0.533$^{\cdot}$	−0.071	−0.562	0.150	0.386	−0.130
	(0.247)	(0.381)	(0.788)	(0.302)	(0.381)	(0.819)	(0.252)	(0.374)	(0.419)	(0.277)	(0.422)	(1.007)	(0.307)	(0.424)	(1.227)
Observations	1545	345	255	1545	345	255	1545	345	255	1545	345	255	1545	345	255
R^2	0.458	0.455	0.616	0.317	0.455	0.478	0.384	0.385	0.562	0.263	0.325	0.358	0.300	0.363	0.403
Adj. R^2	0.454	0.434	0.595	0.311	0.434	0.450	0.379	0.361	0.538	0.257	0.298	0.323	0.294	0.338	0.371
RMSE	1.168	1.245	1.216	1.267	1.245	1.370	1.091	1.232	1.227	1.221	1.235	1.381	1.239	1.319	1.415

Table 30: DV8: General policy support , DV9: Simplified approval procedures, DV10: Advisory services for farmers, DV11: Increased one-off payments, DV12: Financial support for large projects. Statistical significance was derived from linear least squares regression and two-sided tests. Respective standard errors are reported in brackets and significant effects are indicated with: *** for $p < 0.001$, ** for $p < 0.01$, * for $p < 0.05$ and $\cdot$ for $p < 0.1$.

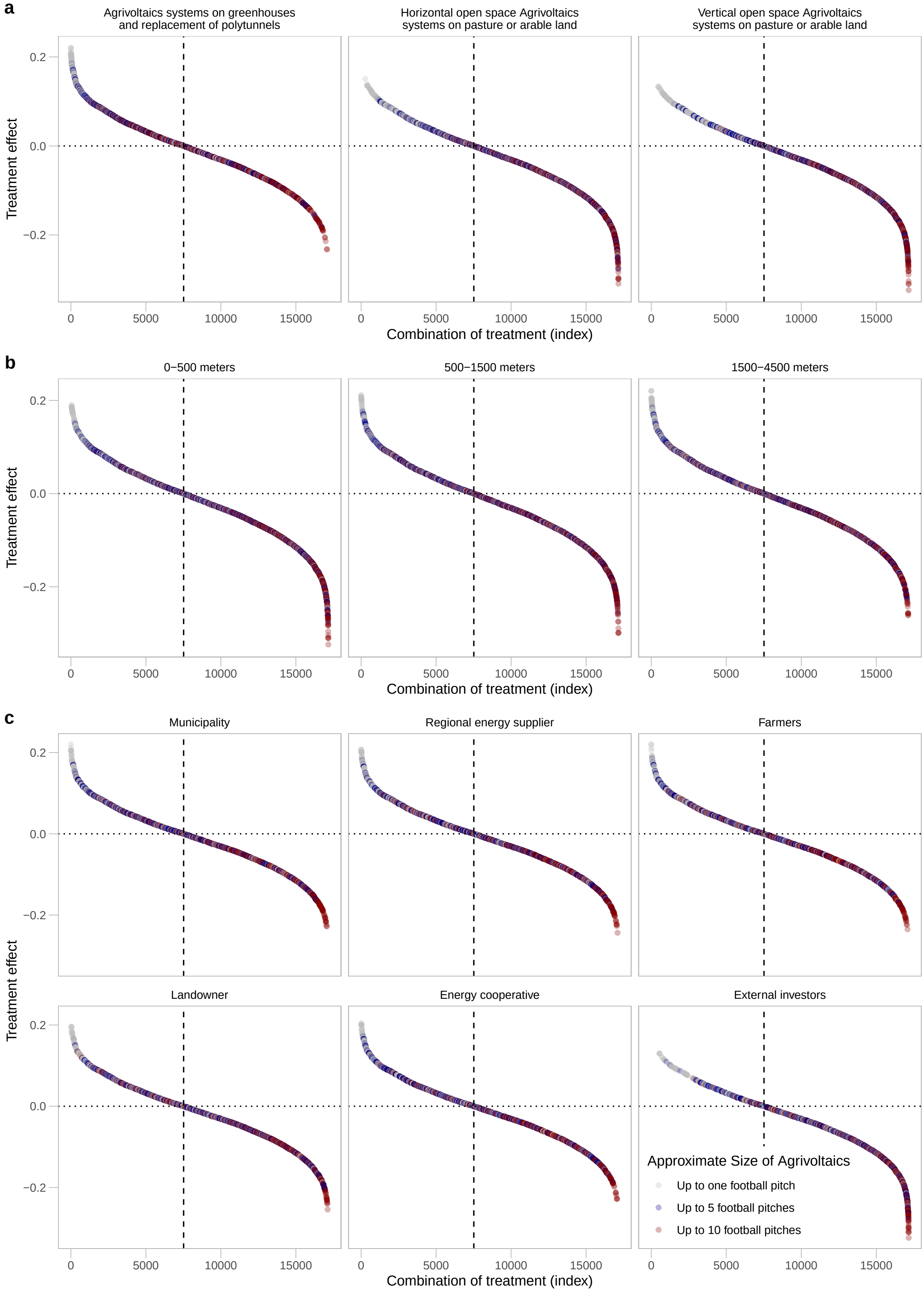

SI Figure 12: **Treatment-Treatment interactions with the highest and lowest treatment effects.** The treatment-treatment interactions are ordered by the size of the treatment effect in the conjoint survey experiment. Agrivoltaics type (in panel a), Distance to Residency (in b), and ownership (in c) are shown, where colors indicate the approximate size of the agrivoltaics project. SI Table 31 and SI Table 32 below show the largest and smallest treatment-treatment interactions with the corresponding attributes. The total N is 17'160 observations for 2145 respondents.

SI Table 31: 50 treatment combinations with the highest Average Marginal Interaction Effect (AMIE)

AMIE	Agrivoltaics Type	Approximate Size of Agrivoltaics	Distance to Residency	Ownership	Impact on Food Production	Impact on Local Energy Production	Impact on Farmers Income
0.2201832	Agrivoltaics systems on greenhouses and replacement of polytunnels	Up to one football pitch	1500-4500 meters	Farmers	0-5% reduction in crop yield	41-80% increase in own production	41-80% higher income
0.2201832	Agrivoltaics systems on greenhouses and replacement of polytunnels	Up to one football pitch	1500-4500 meters	Farmers	0-5% reduction in crop yield	41-80% increase in own production	41-80% higher income
0.2201675	Agrivoltaics systems on greenhouses and replacement of polytunnels	Up to one football pitch	1500-4500 meters	Municipality	0-5% reduction in crop yield	41-80% increase in own production	41-80% higher income
0.2112815	Agrivoltaics systems on greenhouses and replacement of polytunnels	Up to one football pitch	500-1500 meters	Farmers	0-5% reduction in crop yield	41-80% increase in own production	41-80% higher income
0.2112658	Agrivoltaics systems on greenhouses and replacement of polytunnels	Up to one football pitch	500-1500 meters	Municipality	0-5% reduction in crop yield	41-80% increase in own production	41-80% higher income
0.2071360	Agrivoltaics systems on greenhouses and replacement of polytunnels	Up to one football pitch	500-1500 meters	Regional energy supplier	0-5% reduction in crop yield	41-80% increase in own production	41-80% higher income
0.2071360	Agrivoltaics systems on greenhouses and replacement of polytunnels	Up to one football pitch	500-1500 meters	Regional energy supplier	0-5% reduction in crop yield	41-80% increase in own production	41-80% higher income
0.2071360	Agrivoltaics systems on greenhouses and replacement of polytunnels	Up to one football pitch	500-1500 meters	Regional energy supplier	0-5% reduction in crop yield	41-80% increase in own production	41-80% higher income
0.2052160	Agrivoltaics systems on greenhouses and replacement of polytunnels	Up to one football pitch	1500-4500 meters	Energy cooperative	0-5% reduction in crop yield	21-40% increase in own production	41-80% higher income
0.2049386	Agrivoltaics systems on greenhouses and replacement of polytunnels	Up to one football pitch	1500-4500 meters	Farmers	0-5% reduction in crop yield	41-80% increase in own production	11-20% higher income
0.2049230	Agrivoltaics systems on greenhouses and replacement of polytunnels	Up to one football pitch	1500-4500 meters	Municipality	0-5% reduction in crop yield	41-80% increase in own production	21-40% higher income
0.2049230	Agrivoltaics systems on greenhouses and replacement of polytunnels	Up to one football pitch	1500-4500 meters	Municipality	0-5% reduction in crop yield	41-80% increase in own production	21-40% higher income
0.2049230	Agrivoltaics systems on greenhouses and replacement of polytunnels	Up to one football pitch	500-1500 meters	Municipality	0-5% reduction in crop yield	41-80% increase in own production	21-40% higher income
0.2049230	Agrivoltaics systems on greenhouses and replacement of polytunnels	Up to one football pitch	500-1500 meters	Municipality	0-5% reduction in crop yield	41-80% increase in own production	6-10% higher income
0.2049230	Agrivoltaics systems on greenhouses and replacement of polytunnels	Up to one football pitch	1500-4500 meters	Municipality	0-5% reduction in crop yield	41-80% increase in own production	21-40% higher income
0.2049230	Agrivoltaics systems on greenhouses and replacement of polytunnels	Up to one football pitch	1500-4500 meters	Municipality	0-5% reduction in crop yield	41-80% increase in own production	11-20% higher income
0.2007932	Agrivoltaics systems on greenhouses and replacement of polytunnels	Up to one football pitch	1500-4500 meters	Energy cooperative	0-5% reduction in crop yield	41-80% increase in own production	11-20% higher income
0.2007932	Agrivoltaics systems on greenhouses and replacement of polytunnels	Up to one football pitch	1500-4500 meters	Energy cooperative	0-5% reduction in crop yield	41-80% increase in own production	11-20% higher income
0.2007932	Agrivoltaics systems on greenhouses and replacement of polytunnels	Up to one football pitch	1500-4500 meters	Regional energy supplier	0-5% reduction in crop yield	41-80% increase in own production	11-20% higher income
0.2007932	Agrivoltaics systems on greenhouses and replacement of polytunnels	Up to one football pitch	500-1500 meters	Regional energy supplier	0-5% reduction in crop yield	41-80% increase in own production	11-20% higher income
0.2007932	Agrivoltaics systems on greenhouses and replacement of polytunnels	Up to one football pitch	500-1500 meters	Regional energy supplier	0-5% reduction in crop yield	41-80% increase in own production	21-40% higher income
0.2007932	Agrivoltaics systems on greenhouses and replacement of polytunnels	Up to one football pitch	500-1500 meters	Regional energy supplier	0-5% reduction in crop yield	41-80% increase in own production	11-20% higher income
0.2007932	Agrivoltaics systems on greenhouses and replacement of polytunnels	Up to one football pitch	1500-4500 meters	Regional energy supplier	0-5% reduction in crop yield	41-80% increase in own production	11-20% higher income
0.2007932	Agrivoltaics systems on greenhouses and replacement of polytunnels	Up to one football pitch	500-1500 meters	Regional energy supplier	0-5% reduction in crop yield	41-80% increase in own production	6-10% higher income
0.2007932	Agrivoltaics systems on greenhouses and replacement of polytunnels	Up to one football pitch	500-1500 meters	Regional energy supplier	0-5% reduction in crop yield	41-80% increase in own production	6-10% higher income
0.2007932	Agrivoltaics systems on greenhouses and replacement of polytunnels	Up to one football pitch	1500-4500 meters	Energy cooperative	0-5% reduction in crop yield	41-80% increase in own production	11-20% higher income
0.2007932	Agrivoltaics systems on greenhouses and replacement of polytunnels	Up to one football pitch	1500-4500 meters	Energy cooperative	0-5% reduction in crop yield	41-80% increase in own production	11-20% higher income
0.2007932	Agrivoltaics systems on greenhouses and replacement of polytunnels	Up to one football pitch	500-1500 meters	Regional energy supplier	0-5% reduction in crop yield	41-80% increase in own production	21-40% higher income
0.2007932	Agrivoltaics systems on greenhouses and replacement of polytunnels	Up to one football pitch	500-1500 meters	Energy cooperative	0-5% reduction in crop yield	41-80% increase in own production	6-10% higher income
0.2007932	Agrivoltaics systems on greenhouses and replacement of polytunnels	Up to one football pitch	1500-4500 meters	Regional energy supplier	0-5% reduction in crop yield	41-80% increase in own production	6-10% higher income
0.2007932	Agrivoltaics systems on greenhouses and replacement of polytunnels	Up to one football pitch	500-1500 meters	Energy cooperative	0-5% reduction in crop yield	41-80% increase in own production	11-20% higher income
0.1963143	Agrivoltaics systems on greenhouses and replacement of polytunnels	Up to one football pitch	500-1500 meters	Energy cooperative	0-5% reduction in crop yield	21-40% increase in own production	41-80% higher income
0.1955652	Agrivoltaics systems on greenhouses and replacement of polytunnels	Up to one football pitch	1500-4500 meters	Landowner	0-5% reduction in crop yield	41-80% increase in own production	11-20% higher income
0.1955652	Agrivoltaics systems on greenhouses and replacement of polytunnels	Up to one football pitch	1500-4500 meters	Landowner	0-5% reduction in crop yield	41-80% increase in own production	6-10% higher income
0.1955652	Agrivoltaics systems on greenhouses and replacement of polytunnels	Up to one football pitch	1500-4500 meters	Landowner	0-5% reduction in crop yield	41-80% increase in own production	11-20% higher income
0.1955652	Agrivoltaics systems on greenhouses and replacement of polytunnels	Up to one football pitch	500-1500 meters	Landowner	0-5% reduction in crop yield	41-80% increase in own production	11-20% higher income
0.1954288	Agrivoltaics systems on greenhouses and replacement of polytunnels	Up to one football pitch	1500-4500 meters	Municipality	0-5% reduction in crop yield	41-80% increase in own production	0-5% higher income
0.1954288	Agrivoltaics systems on greenhouses and replacement of polytunnels	Up to one football pitch	1500-4500 meters	Municipality	0-5% reduction in crop yield	41-80% increase in own production	0-5% higher income
0.1954288	Agrivoltaics systems on greenhouses and replacement of polytunnels	Up to one football pitch	1500-4500 meters	Municipality	0-5% reduction in crop yield	41-80% increase in own production	0-5% higher income
0.1917668	Agrivoltaics systems on greenhouses and replacement of polytunnels	Up to one football pitch	1500-4500 meters	Farmers	0-5% reduction in crop yield	6-10% increase in own production	41-80% higher income
0.1917668	Agrivoltaics systems on greenhouses and replacement of polytunnels	Up to one football pitch	1500-4500 meters	Farmers	0-5% reduction in crop yield	6-10% increase in own production	41-80% higher income
0.1917511	Agrivoltaics systems on greenhouses and replacement of polytunnels	Up to one football pitch	1500-4500 meters	Municipality	0-5% reduction in crop yield	6-10% increase in own production	41-80% higher income
0.1912990	Agrivoltaics systems on greenhouses and replacement of polytunnels	Up to one football pitch	500-1500 meters	Regional energy supplier	0-5% reduction in crop yield	41-80% increase in own production	0-5% higher income
0.1912990	Agrivoltaics systems on greenhouses and replacement of polytunnels	Up to one football pitch	500-1500 meters	Regional energy supplier	0-5% reduction in crop yield	41-80% increase in own production	0-5% higher income
0.1899714	Agrivoltaics systems on greenhouses and replacement of polytunnels	Up to one football pitch	1500-4500 meters	Energy cooperative	0-5% reduction in crop yield	21-40% increase in own production	6-10% higher income
0.1899714	Agrivoltaics systems on greenhouses and replacement of polytunnels	Up to one football pitch	500-1500 meters	Energy cooperative	0-5% reduction in crop yield	21-40% increase in own production	6-10% higher income
0.1898109	Agrivoltaics systems on greenhouses and replacement of polytunnels	Up to one football pitch	0-500 meters	Farmers	0-5% reduction in crop yield	41-80% increase in own production	41-80% higher income
0.1898109	Agrivoltaics systems on greenhouses and replacement of polytunnels	Up to one football pitch	0-500 meters	Farmers	0-5% reduction in crop yield	41-80% increase in own production	41-80% higher income
0.1891762	Agrivoltaics systems on greenhouses and replacement of polytunnels	Up to one football pitch	1500-4500 meters	Farmers	0-5% reduction in crop yield	21-40% increase in own production	41-80% higher income
0.1891606	Agrivoltaics systems on greenhouses and replacement of polytunnels	Up to one football pitch	1500-4500 meters	Municipality	0-5% reduction in crop yield	21-40% increase in own production	41-80% higher income

SI Table 32: 50 treatment combinations with the lowest Average Marginal Interaction Effect (AMIE)

AMIE	Agrivoltaics Type	Approximate Size of Agrivoltaics	Distance to Residency	Ownership	Impact on Food Production	Impact on Local Energy Production	Impact on Farmers Income
-0.2520608	Vertical open space Agrivoltaics systems on pasture or arable land	Up to 10 football pitches	500-1500 meters	External investors	41-80% reduction in crop yield	0-5% increase in own production	21-40% higher income
-0.2527872	Horizontal open space Agrivoltaics systems on pasture or arable land	Up to 10 football pitches	0-500 meters	External investors	41-80% reduction in crop yield	6-10% increase in own production	21-40% higher income
-0.2527872	Horizontal open space Agrivoltaics systems on pasture or arable land	Up to 10 football pitches	0-500 meters	External investors	41-80% reduction in crop yield	6-10% increase in own production	11-20% higher income
-0.2542265	Vertical open space Agrivoltaics systems on pasture or arable land	Up to 10 football pitches	0-500 meters	Landowner	41-80% reduction in crop yield	0-5% increase in own production	6-10% higher income
-0.2548971	Vertical open space Agrivoltaics systems on pasture or arable land	Up to 10 football pitches	0-500 meters	External investors	41-80% reduction in crop yield	21-40% increase in own production	11-20% higher income
-0.2548971	Vertical open space Agrivoltaics systems on pasture or arable land	Up to 10 football pitches	0-500 meters	External investors	41-80% reduction in crop yield	21-40% increase in own production	11-20% higher income
-0.2555023	Horizontal open space Agrivoltaics systems on pasture or arable land	Up to 5 football pitches	500-1500 meters	External investors	41-80% reduction in crop yield	0-5% increase in own production	0-5% higher income
-0.2566873	Horizontal open space Agrivoltaics systems on pasture or arable land	Up to 10 football pitches	1500-4500 meters	External investors	41-80% reduction in crop yield	0-5% increase in own production	6-10% higher income
-0.2567105	Vertical open space Agrivoltaics systems on pasture or arable land	Up to 10 football pitches	1500-4500 meters	External investors	41-80% reduction in crop yield	0-5% increase in own production	0-5% higher income
-0.2567105	Vertical open space Agrivoltaics systems on pasture or arable land	Up to 10 football pitches	1500-4500 meters	External investors	41-80% reduction in crop yield	0-5% increase in own production	0-5% higher income
-0.2570597	Vertical open space Agrivoltaics systems on pasture or arable land	Up to one football pitch	0-500 meters	External investors	41-80% reduction in crop yield	0-5% increase in own production	6-10% higher income
-0.2570909	Vertical open space Agrivoltaics systems on pasture or arable land	Up to 10 football pitches	1500-4500 meters	External investors	41-80% reduction in crop yield	6-10% increase in own production	6-10% higher income
-0.2576801	Vertical open space Agrivoltaics systems on pasture or arable land	Up to 10 football pitches	500-1500 meters	External investors	41-80% reduction in crop yield	11-20% increase in own production	6-10% higher income
-0.2576801	Vertical open space Agrivoltaics systems on pasture or arable land	Up to 10 football pitches	500-1500 meters	External investors	41-80% reduction in crop yield	11-20% increase in own production	6-10% higher income
-0.2605496	Horizontal open space Agrivoltaics systems on pasture or arable land	Up to 10 football pitches	0-500 meters	External investors	41-80% reduction in crop yield	0-5% increase in own production	41-80% higher income
-0.2608072	Horizontal open space Agrivoltaics systems on pasture or arable land	Up to 10 football pitches	500-1500 meters	External investors	41-80% reduction in crop yield	11-20% increase in own production	0-5% higher income
-0.2608072	Horizontal open space Agrivoltaics systems on pasture or arable land	Up to 10 football pitches	1500-4500 meters	External investors	41-80% reduction in crop yield	11-20% increase in own production	0-5% higher income
-0.2608072	Horizontal open space Agrivoltaics systems on pasture or arable land	Up to 10 football pitches	1500-4500 meters	External investors	41-80% reduction in crop yield	6-10% increase in own production	0-5% higher income
-0.2609532	Vertical open space Agrivoltaics systems on pasture or arable land	Up to 10 football pitches	0-500 meters	External investors	41-80% reduction in crop yield	6-10% increase in own production	41-80% higher income
-0.2615315	Vertical open space Agrivoltaics systems on pasture or arable land	Up to 5 football pitches	0-500 meters	External investors	41-80% reduction in crop yield	11-20% increase in own production	6-10% higher income
-0.2615315	Vertical open space Agrivoltaics systems on pasture or arable land	Up to 5 football pitches	0-500 meters	External investors	41-80% reduction in crop yield	11-20% increase in own production	6-10% higher income
-0.2615423	Vertical open space Agrivoltaics systems on pasture or arable land	Up to 10 football pitches	0-500 meters	External investors	41-80% reduction in crop yield	11-20% increase in own production	41-80% higher income
-0.2615423	Vertical open space Agrivoltaics systems on pasture or arable land	Up to 10 football pitches	0-500 meters	External investors	41-80% reduction in crop yield	11-20% increase in own production	41-80% higher income
-0.2628200	Horizontal open space Agrivoltaics systems on pasture or arable land	Up to 5 football pitches	0-500 meters	External investors	41-80% reduction in crop yield	11-20% increase in own production	0-5% higher income
-0.2668925	Horizontal open space Agrivoltaics systems on pasture or arable land	Up to 10 football pitches	0-500 meters	External investors	41-80% reduction in crop yield	0-5% increase in own production	21-40% higher income
-0.2668925	Horizontal open space Agrivoltaics systems on pasture or arable land	Up to 10 football pitches	0-500 meters	External investors	41-80% reduction in crop yield	0-5% increase in own production	21-40% higher income
-0.2672960	Vertical open space Agrivoltaics systems on pasture or arable land	Up to 10 football pitches	0-500 meters	External investors	41-80% reduction in crop yield	6-10% increase in own production	21-40% higher income
-0.2672960	Vertical open space Agrivoltaics systems on pasture or arable land	Up to 10 football pitches	0-500 meters	External investors	41-80% reduction in crop yield	6-10% increase in own production	21-40% higher income
-0.2672960	Vertical open space Agrivoltaics systems on pasture or arable land	Up to 10 football pitches	0-500 meters	External investors	41-80% reduction in crop yield	6-10% increase in own production	11-20% higher income
-0.2678852	Vertical open space Agrivoltaics systems on pasture or arable land	Up to 10 football pitches	0-500 meters	External investors	41-80% reduction in crop yield	11-20% increase in own production	11-20% higher income
-0.2716147	Vertical open space Agrivoltaics systems on pasture or arable land	Up to 10 football pitches	0-500 meters	External investors	41-80% reduction in crop yield	21-40% increase in own production	0-5% higher income
-0.2716147	Vertical open space Agrivoltaics systems on pasture or arable land	Up to 10 football pitches	0-500 meters	External investors	41-80% reduction in crop yield	21-40% increase in own production	0-5% higher income
-0.2746939	Horizontal open space Agrivoltaics systems on pasture or arable land	Up to 10 football pitches	500-1500 meters	External investors	41-80% reduction in crop yield	0-5% increase in own production	6-10% higher income
-0.2746939	Horizontal open space Agrivoltaics systems on pasture or arable land	Up to 10 football pitches	500-1500 meters	External investors	41-80% reduction in crop yield	0-5% increase in own production	6-10% higher income
-0.2750584	Vertical open space Agrivoltaics systems on pasture or arable land	Up to 10 football pitches	0-500 meters	External investors	41-80% reduction in crop yield	0-5% increase in own production	41-80% higher income
-0.2769483	Horizontal open space Agrivoltaics systems on pasture or arable land	Up to 5 football pitches	0-500 meters	External investors	41-80% reduction in crop yield	0-5% increase in own production	0-5% higher income
-0.2814013	Vertical open space Agrivoltaics systems on pasture or arable land	Up to 10 football pitches	0-500 meters	External investors	41-80% reduction in crop yield	0-5% increase in own production	21-40% higher income
-0.2814013	Vertical open space Agrivoltaics systems on pasture or arable land	Up to 10 football pitches	0-500 meters	External investors	41-80% reduction in crop yield	0-5% increase in own production	11-20% higher income
-0.2814013	Vertical open space Agrivoltaics systems on pasture or arable land	Up to 10 football pitches	0-500 meters	External investors	41-80% reduction in crop yield	0-5% increase in own production	21-40% higher income
-0.2833429	Horizontal open space Agrivoltaics systems on pasture or arable land	Up to 10 football pitches	0-500 meters	External investors	41-80% reduction in crop yield	41-80% increase in own production	0-5% higher income
-0.2892028	Vertical open space Agrivoltaics systems on pasture or arable land	Up to 10 football pitches	500-1500 meters	External investors	41-80% reduction in crop yield	0-5% increase in own production	6-10% higher income
-0.2957554	Horizontal open space Agrivoltaics systems on pasture or arable land	Up to 10 football pitches	0-500 meters	External investors	41-80% reduction in crop yield	21-40% increase in own production	0-5% higher income
-0.2988577	Horizontal open space Agrivoltaics systems on pasture or arable land	Up to 10 football pitches	500-1500 meters	External investors	41-80% reduction in crop yield	0-5% increase in own production	0-5% higher income
-0.2988577	Horizontal open space Agrivoltaics systems on pasture or arable land	Up to 10 football pitches	500-1500 meters	External investors	41-80% reduction in crop yield	0-5% increase in own production	0-5% higher income
-0.2988577	Horizontal open space Agrivoltaics systems on pasture or arable land	Up to 10 football pitches	500-1500 meters	External investors	41-80% reduction in crop yield	0-5% increase in own production	0-5% higher income
-0.3040576	Vertical open space Agrivoltaics systems on pasture or arable land	Up to 10 football pitches	0-500 meters	External investors	41-80% reduction in crop yield	0-5% increase in own production	0-5% higher income
-0.3093771	Horizontal open space Agrivoltaics systems on pasture or arable land	Up to 10 football pitches	0-500 meters	External investors	41-80% reduction in crop yield	0-5% increase in own production	6-10% higher income
-0.3097807	Vertical open space Agrivoltaics systems on pasture or arable land	Up to 10 football pitches	0-500 meters	External investors	41-80% reduction in crop yield	6-10% increase in own production	6-10% higher income
-0.3103699	Vertical open space Agrivoltaics systems on pasture or arable land	Up to 10 football pitches	0-500 meters	External investors	41-80% reduction in crop yield	11-20% increase in own production	6-10% higher income
-0.3238860	Vertical open space Agrivoltaics systems on pasture or arable land	Up to 10 football pitches	0-500 meters	External investors	41-80% reduction in crop yield	0-5% increase in own production	6-10% higher income

SI Section D - Codebook and survey instrument

Codebook for «Agrivoltaics can reduce political polarization and local opposition to solar energy on land»

Contents

1 Control variables 3
2 Treatment variable 5
3 Comprehension/Manipulation check 6
4 Dependent variables 8
5 Conjoint analysis 10
6 Other variables 16

1 Control variables

1.1 age

Variable name	age
Dataset	data
Question text	How old are you (in years)?
Item text	
Variable type	Numeric
Variable range	18 - 79

1.2 gender

Variable name	gender
Dataset	data
Question text	Which gender do you feel you belong to?
Variable type	Numeric
Item text	
Variable range	1 (Male), 2 (Female)

Variable name	gender_f
Dataset	data
Question text	Which gender do you feel you belong to?
Variable type	Factor
Item text	
Variable range	Male, Female

1.3 environment_score

Average of three different variables (1.3.1-1.3.3) measuring the respondent's attitude towards the environment.

Variable name	environment_score
Dataset	data
Question text	None
Item text	
Variable type	Numeric
Variable range	1-7

1.3.1 environ_behaviour

Variable name	environ_behaviour
Dataset	data
Question text	Please indicate whether you agree or disagree with the following statements
Item text	Environmentally friendly behavior is an important part of my being.
Variable type	Numeric
Variable range	1 (Completely disagree)-7 (Completely agree)

1.3.2 crisis_exag

The scale has been manually inversed compared to the coding in the survey to ensure the same direction as the other two variables.

Variable name	crisis_exag
Dataset	data
Question text	Please indicate whether you agree or disagree with the following statements
Item text	The so-called "environmental crisis" facing humanity is greatly exaggerated.
Variable type	Numeric
Variable range	1 (Completely agree)-7 (Completely disagree)

1.3.3 environment_disaster

Variable name	environment_disaster
Dataset	data
Question text	Please indicate whether you agree or disagree with the following statements
Item text	If things continue as they are, we will soon experience a major environmental disaster.
Variable type	Numeric
Variable range	1 (Completely disagree)-7 (Completely agree)

1.4 familiar_agri_pv

Variable name	familiar_agri_pv
Dataset	data
Question text	How familiar are you, if at all, with agri-photovoltaics?
Item text	
Variable type	Numeric
Variable range	1 (Not familiar)-7 (Very familiar)

1.5 like_energy_agri_pv

Variable name	like_energy_agri_pv
Dataset	data
Introduction text	The following questions continue to ask your opinion on agri-photovoltaics, a dual land use concept that combines the cultivation of agricultural crops with the installation of solar panels on land.
Question text	I find energy from agri-photovoltaics
Item txt	
Variable type	Numeric
Variable range	1 (Very bad)-7 (Very good)

1.6 urban_rural

The degree of urbanization of the place a respondent is living in is based on the real spatial typology defined by the Swiss Federal Statistical Office

Variable name	urban_rural
Dataset	data
Introduction text	
Question text	
Item text	
Variable type	Numeric
Variable range	1 (Urban), 2 (Suburban), 3 (Rural)

Variable name	urban_rural_f
Dataset	data
Introduction text	
Question text	
Item text	
Variable type	Factor
Variable range	Urban, Suburban, Rural

2 Treatment variable

Variable name	NIMBY
Dataset	data
Introduction text	
Question text	
Item text	
Variable type	Numeric
Variable range	0 (Control), 1 (Treatment)

The treatment is designed as follows:

English

Agri-photovoltaics (Agri-PV) is a method of producing solar power on agricultural land and growing crops at the same time. Depending on the area and solar radiation, the potential for building Agri-PV systems varies in Switzerland. To produce sufficient solar power, only some of the areas in Switzerland - those with high electricity yield potential - actually need to be developed.

Now please take a close look at the following map.

The map shows your place of residence as well as the realistic potential (large or small) for building Agri-PV systems around your place of residence.

- 0-500 meters (inner circle) around your residence is the potential for building Agri-PV systems **small**
- 500-1500 meters (middle circle) around your residence is the potential for building Agri-PV systems **small**
- 1500-4500 meters (outer circle) around your residence is the potential for building Agri-PV systems **large**

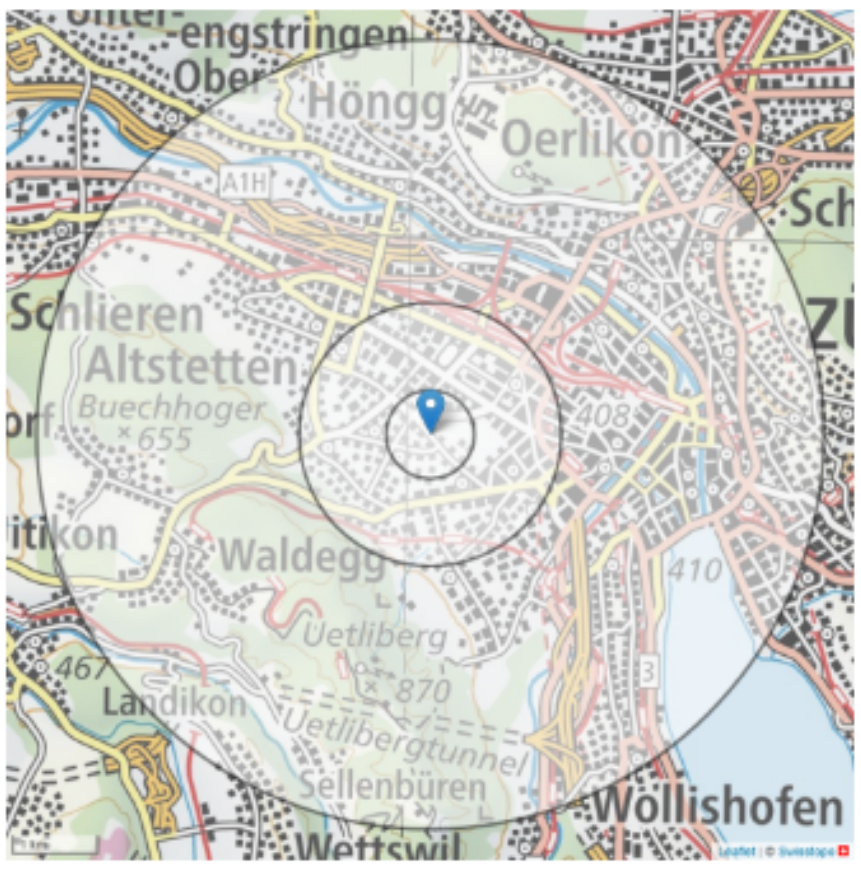

© Data: swisstopo

The control group was presented with the following:

English

Agri-photovoltaics (Agri-PV) is a method of producing solar power on agricultural land and growing crops at the same time. The potential for building agri-PV systems in Switzerland varies depending on the area and the amount of sunlight. To produce sufficient solar power, only part of the land in Switzerland - that with high electricity yield potential - actually needs to be built on.

→

3 Comprehension/Manipulation check

3.1 potential_known_1

Variable name	potential_known_1
Dataset	data
Introduction text	
Question text	To your knowledge, how big or small is the potential for building agri-PV systems in the area around your apartment/house?
Item text	0-500 meters around your residence
Variable type	Numeric

Variable range	0 (Small), 1 (Large)

3.2 potential_known_2

Variable name	potential_known_2
Dataset	data
Introduction text	
Question text	To your knowledge, how big or small is the potential for building agri-PV systems in the area around your apartment/house?
Item text	500-1500 meters around your residence
Variable type	Numeric
Variable range	0 (Small), 1 (Large)

3.3 potential_known_3

Variable name	potential_known_3
Dataset	data
Introduction text	
Question text	To your knowledge, how big or small is the potential for building agri-PV systems in the area around your apartment/house?
Item text	1500-4500 meters around your residence
Variable type	Numeric
Variable range	0 (Small), 1 (Large)

3.4 personal_advantage

Variable name	personal_advantage
Dataset	data
Introduction text	
Question text	For you personally: Do you see the construction of agri-photovoltaic projects in Switzerland as an advantage or a disadvantage?
Item text	
Variable type	Numeric
Variable range	1 (Very disadvantageous) – 7 (Very advantageous)

3.5 ch_advantage

Variable name	ch_advantage
Dataset	data
Introduction text	
Question text	For Switzerland and its population as a whole: Do you consider the construction of agri-photovoltaic projects in Switzerland to be an advantage or a disadvantage?
Item text	
Variable type	Numeric
Variable range	1 (Very disadvantageous) – 7 (Very advantageous)

4 Dependent variables

4.1 attitude_expansion_small

Variable name	attitude_expansion_small
Dataset	data
Introduction text	
Question text	What is your personal attitude towards the expansion of the following agri-photovoltaic systems?
Item text	Small agri-PV system (up to 1 football field, approx. 1ha)
Variable type	Numeric
Variable range	1 (I completely oppose) – 7 (I completely support)

4.2 attitude_expansion_medium

Variable name	attitude_expansion_medium
Dataset	data
Introduction text	
Question text	What is your personal attitude towards the expansion of the following agri-photovoltaic systems?
Item text	Medium-sized agri-PV plant (up to 5 football fields, approx. 5ha)
Variable type	Numeric
Variable range	1 (I completely oppose) – 7 (I completely support)

4.3 attitude_expansion_large

Variable name	attitude_expansion_large
Dataset	data
Introduction text	
Question text	What is your personal attitude towards the expansion of the following agri-photovoltaic systems?
Item text	Large agri-PV plant (up to 10 football fields, approx. 10ha)
Variable type	Numeric
Variable range	1 (I completely oppose) – 7 (I completely support)

4.4 attitude_expansion_nearby

Variable name	attitude_expansion_nearby
Dataset	data
Introduction text	Imagine that an agri-photovoltaic project is planned near where you live. Together with other people from your neighborhood, you are asked to give your opinion on the project.
Question text	What is your personal attitude to the expansion of agri-photovoltaic systems in your immediate neighborhood?
Item text	
Variable type	Numeric
Variable range	1 (I completely reject) – 7 (I completely support)

4.5 attitude_expansion

Variable name	attitude_expansion

Dataset	data
Introduction text	
Question text	What is your personal attitude towards the expansion of agri-photovoltaic systems in Switzerland?
Item text	
Variable type	Numeric
Variable range	1 (I completely oppose) – 7 (I completely support)

4.6 support_policies

Variable name	support_policies
Dataset	data
Introduction text	Imagine that the government is planning to change its renewable energy policy. Part of this policy is to increase the number of agri-photovoltaic projects in the country. You are asked to give your opinion on this policy.
Question text	
Item text	I intend to support policies aimed at increasing the number of agri-photovoltaic projects in the country.
Variable type	Numeric
Variable range	1 (Completely disagree) – 7 (Completely agree)

4.7 Policies

4.7.1 support_policy_1

Variable name	support_policy_1
Dataset	data
Introduction text	
Question text	What is your personal attitude to the following concrete political instruments for the expansion of agri-photovoltaic systems in Switzerland?
Item text	Simplified approval procedures
Variable type	Numeric
Variable range	1 (I completely oppose) – 7 (I completely support)

4.7.2 support_policy_2

Variable name	support_policy_2
Dataset	data
Introduction text	
Question text	What is your personal attitude to the following concrete political instruments for the expansion of agri-photovoltaic systems in Switzerland?
Item text	State financial support for agri-PV projects (support with a one-off payment of up to 60 percent of the eligible investment costs for large-scale systems)
Variable type	Numeric
Variable range	1 (I completely oppose) – 7 (I completely support)

4.7.3 support_policy_4

Variable name	support_policy_4

Dataset	data
Introduction text	
Question text	What is your personal attitude to the following concrete political instruments for the expansion of agri-photovoltaic systems in Switzerland?
Item text	Advisory services for farmers to promote the expansion of agri-PV systems on their fields
Variable type	Numeric
Variable range	1 (I completely oppose) – 7 (I completely support)

4.7.4 support_policy_6

Variable name	support_policy_6
Dataset	data
Introduction text	
Question text	What is your personal attitude to the following concrete political instruments for the expansion of agri-photovoltaic systems in Switzerland?
Item text	Increase in the one-off payment for the construction of agri-PV systems
Variable type	Numeric
Variable range	1 (I completely oppose) – 7 (I completely support)

5 Conjoint analysis

5.1 Introduction

The next part of the survey will now look at possible concrete projects for the expansion of solar energy in Switzerland. In particular, we are interested in your opinion on agri-photovoltaics, a dual land use concept that combines the cultivation of agricultural crops with the installation of photovoltaic systems on land. Specifically, we will conduct a thought experiment with you in which we ask you to compare and evaluate alternative projects for the expansion of agri-photovoltaics in Switzerland. Each project has different aspects that can influence your evaluation.

We will now start the thought experiment on the construction of possible agri-PV systems in Switzerland. In four different voting rounds, we will present you with two alternative investment options side by side. In each round, please compare the two options for agri-PV systems carefully and indicate which option you prefer and to what extent you support or reject the respective systems. Thank you. Now let's get started.

ETH zürich

English

Below you will find the most important aspects and characteristics that distinguish possible agri-photovoltaic (agri-PV) systems in Switzerland.

- Type of agri-PV system

Agri-PV systems on greenhouses and replacement of polytunnels

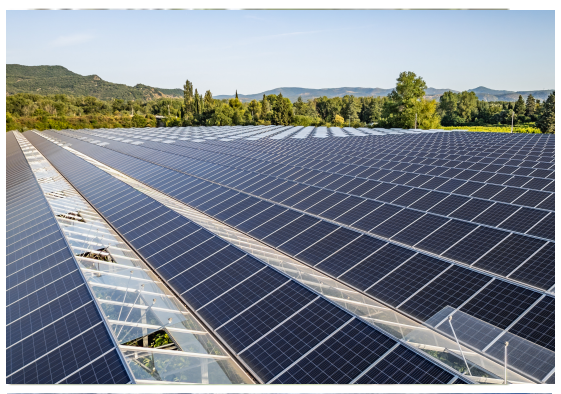

©Daniel–stock.adobe.com

Vertical open space agri-PV systems on pasture or arable land

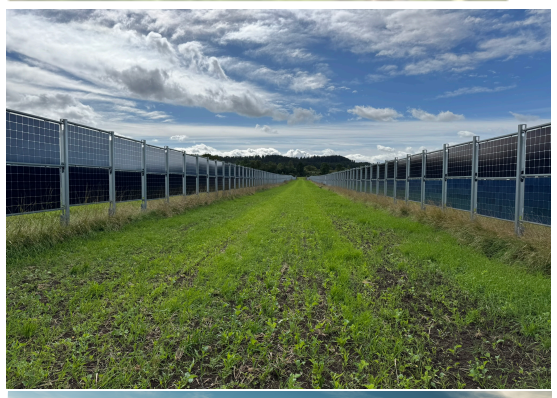

©Jürg Rohrer

Horizontal open space agri-PV system on pasture or arable land

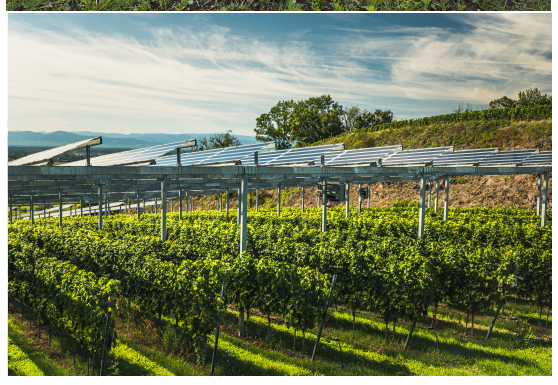

©Conny Pokorny–stock.adobe.com

- Size of the agri-PV system
- Distance of the agri-PV system from your own place of residence
- Ownership structure of the agri-PV system
- Effect of the agri-PV system on the crop yield of the agricultural area
- Effect of the agri-PV system on the electricity production of the municipality
- Effect of the agri-PV system on the income of the farmers

ETH zürich

English

Round 1 of the thought experiment: Please carefully compare the following two options for agri-PV systems in Switzerland.

	Agri-PV Option A	Agri-PV Option B
Type of agri-PV system:	Horizontal open space Agri-PV systems on pasture or arable land	Vertical open space Agri-PV systems on pasture or arable land
System size:	Up to one football pitch (approx. 1ha)	Up to 10 football pitches (approx. 10ha)
Distance from your own place of residence:	1500-4500 meters	0-500 meters
Ownership structure:	External (non-local) investors	Energy cooperative (e.g. local population)
Effect on crop yield of the agricultural area:	21-40% reduction in crop yield	41-80% reduction in crop yield
Effect on electricity production of the municipality:	21-40% increase in own production	21-40% increase in own production
Income of the farmers:	0-5% higher income	6-10% higher income

Which of the two agri-PV investment options do you prefer?

Agri-PV Option A	Agri-PV Option B
○	○

How much would you support or oppose the construction of the two agri-PV plant options?

	Completely oppose	Oppose	Rather oppose	Neither nor	Rather support	Support	Completely support
Agri-PV Option A	○	○	○	○	○	○	○
Agri-PV Option B	○	○	○	○	○	○	○

5.2 Conjoint Attributes

5.2.1 attrib1_lab

Variable name	attrib1_lab
Dataset	exp_dat
Introduction text	
Question text	
Item text	Type of Agri-PV system
Variable type	Factor
Variable range	Agri-PV systems on greenhouses and replacement of polytunnels, Vertical open space agri-PV systems on pasture or arable land, Horizontal open space agri-PV system on pasture or arable land

5.2.2 attrib2_lab

Variable name	attrib2_lab
Dataset	exp_dat

Introduction text	
Question text	
Item text	Size of the Agri-PV system
Variable type	Factor
Variable range	Up to one football pitch, Up to 5 football pitches, Up to 10 football pitches

5.2.3 attrib3_lab

Variable name	attrib3_lab
Dataset	exp_dat
Introduction text	
Question text	Distance of the agri-PV system from your own place of residence
Item text	
Variable type	Factor
Variable range	0-500 meters, 500-1500 meters, 1500-4500 meters

5.2.4 attrib4_lab

Variable name	attrib4_lab
Dataset	exp_dat
Introduction text	
Question text	Ownership structure of the agri-PV system
Item text	
Variable type	Factor
Variable range	Municipality, Regional energy supplier, Farmers, Landowner, Energy cooperative, External investors

5.2.5 attrib5_lab

Variable name	attrin5_lab
Dataset	exp_dat
Introduction text	
Question text	Effect of the agri-PV system on the crop yield of the agricultural area
Item text	
Variable type	Factor
Variable range	0-5% reduction in crop yield, 6-10% reduction in crop yield, 11-20% reduction in crop yield, 21-40% reduction in crop yield, 41-80% reduction in crop yield

5.2.6 attrib6_lab

Variable name	attrin6_lab
Dataset	exp_dat
Introduction text	
Question text	Effect of the agri-PV system on the electricity production of the municipality
Item text	
Variable type	Factor
Variable range	0-5% increase in own production, 6-10% increase in own production, 11-20% increase in own production, 21-40% increase in own production, 41-80% increase in own production

5.2.7 attrib7_lab

Variable name	attrin7_lab
Dataset	exp_dat
Introduction text	
Question text	Effect of the agri-PV system on the income of the farmers
Item text	
Variable type	Factor
Variable range	0-5% higher income, 6-10% higher income, 11-20% higher income, 21-40% higher income, 41-80% higher income

5.3 Conjoint Choice Variables

5.3.1 Round 1

5.3.1.1 Q15.8

Variable name	Q15.8
Dataset	data
Introduction text	
Question text	Which of the two agri-PV investment options do you prefer?
Item text	
Variable type	Numeric
Variable range	1 (Agri-PV Option A), 2 (Agri-PV Option B)

5.3.1.2 Q15.9_1

Variable name	Q15.9_1
Dataset	data
Introduction text	
Question text	How much would you support or oppose the construction of the two agri-PV plant options?
Item text	Agri-PV Option A
Variable type	Numeric
Variable range	1 (Completely oppose) – 7 (Completely support)

5.3.1.3 Q15.9_2

Variable name	Q15.9_2
Dataset	data
Introduction text	
Question text	How much would you support or oppose the construction of the two agri-PV plant options?
Item text	Agri-PV Option B
Variable type	Numeric
Variable range	1 (Completely oppose) – 7 (Completely support)

5.3.2 Round 2

5.3.2.1 Q15.14

Variable name	Q15.14
Dataset	data

Introduction text	
Question text	Which of the two agri-PV investment options do you prefer?
Item text	
Variable type	Numeric
Variable range	1 (Agri-PV Option A), 2 (Agri-PV Option B)

5.3.2.2 Q15.15_1

Variable name	Q15.15_1
Dataset	data
Introduction text	
Question text	How much would you support or oppose the construction of the two agri-PV plant options?
Item text	Agri-PV Option A
Variable type	Numeric
Variable range	1 (Completely oppose) – 7 (Completely support)

5.3.2.3 Q15.15_2

Variable name	Q15.15_2
Dataset	data
Introduction text	
Question text	How much would you support or oppose the construction of the two agri-PV plant options?
Item text	Agri-PV Option B
Variable type	Numeric
Variable range	1 (Completely oppose) – 7 (Completely support)

5.3.3 Round 3

5.3.3.1 Q15.20

Variable name	Q15.20
Dataset	data
Introduction text	
Question text	Which of the two agri-PV investment options do you prefer?
Item text	
Variable type	Numeric
Variable range	1 (Agri-PV Option A), 2 (Agri-PV Option B)

5.3.3.2 Q15.21_1

Variable name	Q15.21_1
Dataset	data
Introduction text	
Question text	How much would you support or oppose the construction of the two agri-PV plant options?
Item text	Agri-PV Option A
Variable type	Numeric
Variable range	1 (Completely oppose) – 7 (Completely support)

5.3.3.3 Q15.21_2

Variable name	Q15.21_2

Dataset	data
Introduction text	
Question text	How much would you support or oppose the construction of the two agri-PV plant options?
Item text	Agri-PV Option B
Variable type	Numeric
Variable range	1 (Completely oppose) – 7 (Completely support)

5.3.4 Round 4

5.3.4.1 Q15.26

Variable name	Q15.26
Dataset	data
Introduction text	
Question text	Which of the two agri-PV investment options do you prefer?
Item text	
Variable type	Numeric
Variable range	1 (Agri-PV Option A), 2 (Agri-PV Option B)

5.3.4.2 Q15.27_1

Variable name	Q15.27_1
Dataset	data
Introduction text	
Question text	How much would you support or oppose the construction of the two agri-PV plant options?
Item text	Agri-PV Option A
Variable type	Numeric
Variable range	1 (Completely oppose) – 7 (Completely support)

5.3.4.3 Q15.27_2

Variable name	Q15.27_2
Dataset	data
Introduction text	
Question text	How much would you support or oppose the construction of the two agri-PV plant options?
Item text	Agri-PV Option B
Variable type	Numeric
Variable range	1 (Completely oppose) – 7 (Completely support)

6 Other variables

6.1 circle

Binary variable is 1 when the potential for Agri-PV is high in any of the three circles that are depicted during the NIMBY-treatment.

Variable name	circle
Dataset	data
Introduction text	
Question text	

Item text	
Variable type	numeric
Variable range	0 (low potential in all circles), 1 (high potential in at least one circle)

6.2 circle1

Variable name	circle1
Dataset	data
Introduction text	
Question text	
Item text	
Variable type	numeric
Variable range	0 (low potential 0m – 500m), 1 (high potential 0-500m)

6.3 circle2

Variable name	circle2
Dataset	data
Introduction text	
Question text	
Item text	
Variable type	numeric
Variable range	0 (low potential 500m – 1500m), 1 (high potential 500-1500m)

6.4 circle3

Variable name	circle3
Dataset	data
Introduction text	
Question text	
Item text	
Variable type	numeric
Variable range	0 (low potential 1500m – 4500m), 1 (high potential 1500-4500m)

6.5 solar_roofs

Variable name	solar_roofs
Dataset	data
Introduction text	
Question text	To what extent do you agree with the following statements: To ensure the Swiss electricity supply in the future, ...
Item text	…more solar power systems should be installed on buildings
Variable type	numeric
Variable range	1 (Completely disagree)-7 (Completely agree)

6.6 solar_open_space

Variable name	solar_open_space
Dataset	data
Introduction text	

Question text	To what extent do you agree with the following statements: To ensure the Swiss electricity supply in the future, ...
Item text	…more solar systems should be installed in open spaces (e.g. meadows, fields, etc.).
Variable type	numeric
Variable range	1 (Completely disagree)-7 (Completely agree)

6.7 Political orientation

Variable name	left_right
Dataset	data
Introduction text	
Question text	Where would you place your own political views on this scale?
Item text	
Variable type	numeric
Variable range	1 (left) – 10 (right)

The variable left_right_centre is created from left_right by splitting into left-leaning (1-4), centric (5-6), and right-leaning (7-10) respondents.

Variable name	left_right_centre
Dataset	data
Introduction text	
Question text	
Item text	
Variable type	Factor
Variable range	Left, Centre, Right

6.8 UserLanguage

Variable name	UserLanguage
Dataset	data
Introduction text	
Question text	
Item text	
Variable type	Character
Variable range	“DE”, “FR”, “EN”
