# Supplementary material for: Agrivoltaics can reduce political polarization and local opposition to solar energy on land
Source: Nat Commun. 2026 Aug 27;17:10263. doi: 10.1038/s41467-026-77141-8 (PMC13616971; doi:10.1038/s41467-026-77141-8)
Supplement: Supplementary file 2 [file 41467_2026_77141_MOESM2_ESM.pdf]

nature portfolio

Corresponding author(s): Lukas Fesenfeld

Last updated by author(s): May 23, 2026

Reporting Summary

Nature Portfolio wishes to improve the reproducibility of the work that we publish. This form provides structure for consistency and transparency in reporting. For further information on Nature Portfolio policies, see our Editorial Policies and the Editorial Policy Checklist.

Statistics

For all statistical analyses, confirm that the following items are present in the figure legend, table legend, main text, or Methods section.

n/a	Confirmed	
☐	☒	The exact sample size (*n*) for each experimental group/condition, given as a discrete number and unit of measurement
☐	☒	A statement on whether measurements were taken from distinct samples or whether the same sample was measured repeatedly
☐	☒	The statistical test(s) used AND whether they are one- or two-sided<br>*Only common tests should be described solely by name; describe more complex techniques in the Methods section.*
☐	☒	A description of all covariates tested
☐	☒	A description of any assumptions or corrections, such as tests of normality and adjustment for multiple comparisons
☐	☒	A full description of the statistical parameters including central tendency (e.g. means) or other basic estimates (e.g. regression coefficient) AND variation (e.g. standard deviation) or associated estimates of uncertainty (e.g. confidence intervals)
☐	☒	For null hypothesis testing, the test statistic (e.g. *F*, *t*, *r*) with confidence intervals, effect sizes, degrees of freedom and *P* value noted<br>*Give P values as exact values whenever suitable.*
☒	☐	For Bayesian analysis, information on the choice of priors and Markov chain Monte Carlo settings
☒	☐	For hierarchical and complex designs, identification of the appropriate level for tests and full reporting of outcomes
☒	☐	Estimates of effect sizes (e.g. Cohen's *d*, Pearson's *r*), indicating how they were calculated

Our web collection on statistics for biologists contains articles on many of the points above.

Software and code

Policy information about availability of computer code

Data collection

The respondents in our survey are drawn from the population register of the Federal Statistical Office (BfS) comprising Swiss residents. The BfS population register mirrors, besides random error and uneven response rates, the Swiss resident population. We used stratified random sampling with the strata language region (i.e. German or French speaking parts of Switzerland), gender (i.e. male or female), and urbanization level (i.e., urban, semi-urban, rural). The ETH Decision Sciences Laboratory invited a random sample of Swiss residents in the French and German speaking part of Switzerland above the age of 18 years via letter (plus one reminder) to participate in our online survey via the survey platform Qualtrics. Anonymized data supporting this study are openly available from 10.5281/zenodo.20266276

Data analysis

Code and data supporting this study (including replication code for analyses, figures and tables) is openly available from 10.5281/zenodo.20266276 The analysis for this paper has been conducted using R version 4.5.2. The following packages have been used:
- cregg 0.3.7
- tidyverse 2.0.0
- ggpubr 0.6.2
- kableExtra 1.4.0
- stringi 1.8.7
- cowplot 1.2.0
- FindIt 1.3.0
- estimatr 1.0.6
- patchwork 1.3.2
- texreg 1.39.5
- emmeans 2.0.1
- scales 1.4.0

- vtable 1.4.8
- openxlsx 4.2.8.1
- dplyr 1.1.4

For manuscripts utilizing custom algorithms or software that are central to the research but not yet described in published literature, software must be made available to editors and reviewers. We strongly encourage code deposition in a community repository (e.g. GitHub). See the Nature Portfolio guidelines for submitting code & software for further information.

Data

Policy information about availability of data

All manuscripts must include a data availability statement. This statement should provide the following information, where applicable:

- Accession codes, unique identifiers, or web links for publicly available datasets
- A description of any restrictions on data availability
- For clinical datasets or third party data, please ensure that the statement adheres to our policy

Anonymized data supporting this study are openly available from 10.5281/zenodo.20266276

Research involving human participants, their data, or biological material

Policy information about studies with human participants or human data. See also policy information about sex, gender (identity/presentation), and sexual orientation and race, ethnicity and racism.

Reporting on sex and gender	Self-reported gender was measured as part of the survey after informed consent by respondents. Respondents had the option to state their gender as female, male or other. The information was used as covariate and descriptive statistics in the analyses. You can find a detailed description of these statistics in the Supplementary Information of the paper.
Reporting on race, ethnicity, or other socially relevant groupings	Race or ethnicity was not measured as part of the survey.
Population characteristics	As shown in SI Section A of the papers' Supplementary Information , our random sample is a close representative approximation of the Swiss resident population in the French and German speaking part of Switzerland above the age of 18 years in terms of age, gender, degree of urbanization, and language region.
Recruitment	The respondents in our survey are drawn from the population register of the Federal Statistical Office (BfS) comprising Swiss residents. The BfS population register mirrors, besides random error and uneven response rates, the Swiss resident population. We used stratified random sampling with the strata language region (i.e. German or French speaking parts of Switzerland), gender (i.e. male or female), and urbanization level (i.e., urban, semi-urban, rural). The ETH Decision Sciences Laboratory invited a random sample of Swiss residents in the French and German speaking part of Switzerland above the age of 18 years via letter (plus one reminder) to participate in our online survey via the survey platform Qualtrics. Self-selection into the survey is given but the final survey is a close representative approximation of the Swiss resident population in the French and German speaking part of Switzerland above the age of 18. Due to randomization of the treatment, self-selection into the treatment was not possible.
Ethics oversight	The study has been pre-registered (AEA RCT Registry https://doi.org/10.1257/rct.13540-1.0) and approved by the ETH Zurich Ethics Commission (EK 2024-N-84, Zurich, 24 April 2024)

Note that full information on the approval of the study protocol must also be provided in the manuscript.

Field-specific reporting

Please select the one below that is the best fit for your research. If you are not sure, read the appropriate sections before making your selection.

☐ Life sciences ☒ Behavioural & social sciences ☐ Ecological, evolutionary & environmental sciences

For a reference copy of the document with all sections, see nature.com/documents/nr-reporting-summary-flat.pdf

Behavioural & social sciences study design

All studies must disclose on these points even when the disclosure is negative.

Study description	We used quantitative data in this experimental study. We conducted a field-embedded, pre-registered, geolocated information and conjoint experiment to analyze how personalized information about realistic local agrivoltaics building potential and the specific technology and project design of agrivoltaics affect public support across ideological groups and urban–rural populations. We fielded the experiment with a representative sample of 2,145 Swiss adult residents in the French- and German-speaking cantons just before a public renewable energy referendum in June 2024. For further details, please see the Method section of the manuscript.
Research sample	The respondents in our survey are drawn from the population register of the Federal Statistical Office (BfS) comprising Swiss

Research sample	residents. The BfS population register mirrors, besides random error and uneven response rates, the Swiss resident population. We used stratified random sampling with the strata language region (i.e. German or French speaking parts of Switzerland), gender (i.e. male or female), and urbanization level (i.e., urban, semi-urban, rural). The ETH Decision Sciences Laboratory invited a random sample of Swiss residents in the French and German speaking part of Switzerland above the age of 18 years via letter (plus one reminder) to participate in our online survey via the survey platform Qualtrics. Self-selection into the survey is given but the final survey is a close representative approximation of the Swiss resident population in the French and German speaking part of Switzerland above the age of 18. The rationale for choosing this sample is to have a high-quality representative sample of the Swiss esident population in the French and German speaking part of Switzerland above the age of 18, relevant for answering the key research questions.
Sampling strategy	We used stratified random sampling with the strata language region (i.e. German or French speaking parts of Switzerland), gender (i.e. male or female), and urbanization level (i.e., urban, semi-urban, rural). Statistical power analyses were conducted prior to the treatment design and study pre-registration to determine the minmum sample size. The sample size in our study has sufficient statistical power.
Data collection	The ETH Decision Sciences Laboratory invited a random sample of 16.500 Swiss residents in the French and German speaking part of Switzerland above the age of 18 years via letter (plus one reminder) to participate in our online survey via the survey platform Qualtrics. Self-selection into the survey is given but the final survey is a close representative approximation of the Swiss resident population in the French and German speaking part of Switzerland above the age of 18. Due to randomization of the treatment, self-selection into the treatment was not possible. Respondents recorded their responses in the online survey via the survey plattform Qualtrics. Researchers were not present during the data collection process and respondents provided their informed consent to participate. The treatment condition was completely randomized and randomization happened automatically through an algorithm. The survey instrument is provided in the replication material and SI.
Timing	The data collection occurred between May 3 and June 8, 2024, in the run-up to a publicly salient national referendum on the Electricity Law.
Data exclusions	We received a total of 3,384 responses, of which 445 responses were incomplete, resulting in a response rate of around 20.5%. After excluding respondents who completed the survey in less than 40 percent of the median response time (44 minutes), as well as unclear responses and non-completes, our final sample consisted of 2,145 Swiss residents.
Non-participation	Our response rate was approx. 20,5%.
Randomization	Respondents were randomly selected for the treatment and control group.

Reporting for specific materials, systems and methods

We require information from authors about some types of materials, experimental systems and methods used in many studies. Here, indicate whether each material, system or method listed is relevant to your study. If you are not sure if a list item applies to your research, read the appropriate section before selecting a response.

Materials & experimental systems

n/a	Involved in the study
☒	☐ Antibodies
☒	☐ Eukaryotic cell lines
☒	☐ Palaeontology and archaeology
☒	☐ Animals and other organisms
☒	☐ Clinical data
☒	☐ Dual use research of concern
☒	☐ Plants

Methods

n/a	Involved in the study
☒	☐ ChIP-seq
☒	☐ Flow cytometry
☒	☐ MRI-based neuroimaging

Plants

Seed stocks	*Report on the source of all seed stocks or other plant material used. If applicable, state the seed stock centre and catalogue number. If plant specimens were collected from the field, describe the collection location, date and sampling procedures.*
Novel plant genotypes	*Describe the methods by which all novel plant genotypes were produced. This includes those generated by transgenic approaches, gene editing, chemical/radiation-based mutagenesis and hybridization. For transgenic lines, describe the transformation method, the number of independent lines analyzed and the generation upon which experiments were performed. For gene-edited lines, describe the editor used, the endogenous sequence targeted for editing, the targeting guide RNA sequence (if applicable) and how the editor was applied.*
Authentication	*Describe any authentication procedures for each seed stock used or novel genotype generated. Describe any experiments used to assess the effect of a mutation and, where applicable, how potential secondary effects (e.g. second site T-DNA insertions, mosiacism, off-target gene editing) were examined.*
