# Supplementary material for: Agrivoltaics can reduce political polarization and local opposition to solar energy on land
Source: Nat Commun. 2026 Aug 27;17:10263. doi: 10.1038/s41467-026-77141-8 (PMC13616971; doi:10.1038/s41467-026-77141-8)
Supplement: Supplementary file 3 [file 41467_2026_77141_MOESM3_ESM.pdf]

nature portfolio

Peer Review File

Agrivoltaics can reduce political polarization and local opposition to solar energy on land

Corresponding Author: Professor Lukas Fesenfeld

This file contains all reviewer reports in order by version, followed by all author rebuttals in order by version.

Version 0:

Reviewer comments:

Reviewer #1

(Remarks to the Author)
This is a very insightful paper based on an original and well-conducted survey experiment addressing a substantively interesting question: the acceptance of solar plants on land. This is a valuable contribution to the vast literature on the acceptance of renewable energy and therefore also interesting to policymakers. I recommend proceeding with the manuscript, but I have the following suggestions for revision.

Importantly, in terms of structure, the manuscript needs significant improvement: First, it should follow a typical research article structure: after the introduction should come the research design and method, followed by the results. At the moment, we learn about the method and survey details far too late – most of it appears only at the very end. Moreover, the results section currently includes methodological information, which is rather unusual. Second, the introduction needs to be substantially rewritten to prepare the readers about what's coming, without already including presentation of data.

The notion of "bipartisanship" in the paper does not make sense given that the study deals with a multiparty context. "Bipartisanship" may be meaningful in the U.S., but certainly not in the context of the case study at hand. Similarly, and more substantively, I wonder whether the analysis should not distinguish simply between "left" and "right," but rather between clearly left-wing voters, centrist voters, and clearly right-wing voters. This would make the analysis more nuanced and avoid lumping together radical leftists (righists) with individuals just slightly left (right) of center. Substantively, it would make more sense to structure the analysis around these categories. This would help to show which type of right-wing voters (more moderate ones?) can be reached by a different design of agrivoltaics.

The paper also lacks substance in some areas, as it does not provide enough information on the political contentious subjects. In particular:

1. Food security and land use conflict: There should be more detail on the tension between land for solar installations and land for food production, in Switzerland and more generally.

2. Ownership questions: The same applies here. The authors include multiple ownership structures in their research design, but the manuscript does not clearly explain how these were analyzed or which specific ownership types are most consequential.

3. Urban/rural divide: This is an issue of obvious importance but hardly explicitly discussed in the paper. I would expect much more on this, also with respect to the empirical analysis – as well as theory. An interesting recent reference is: "Far-right against green: The re-emergence of geographically defined voting patterns and the new environment cleavage in Western Europe" in the European Political Science Review (2025).

Finally, regarding case selection, direct democracy and the referendum is mentioned but never clearly justified as relevant for case selection. Why should we care? Presumably, this is due to issue salience – but this should be made explicit and discussed more clearly. The question of issue salience of solar energy in comparison to other renewables, particularly wind power, merits more discussion more generally. It might be less symbolically charged than wind power and therefore more prone to attitude shift. An interesting recent reference is: "Climate action versus environmental protection? How far-right and environmentalist messengers shape public attitudes towards renewable energy infrastructure in forests" in Environmental

Politics (2025).

The writing would benefit from careful proofreading. For instance, already the second sentence reads oddly, with the word “land” used three times in a single sentence.

P. 3: „To address 82 this research gap, we experimentally analyze how personalized geolocated information 83 about the realistic Agri-PV building potential and different technology and project 84 design characteristics of Agri-PV jointly affect public support across ideological groups.“: Why „jointly“ here?

Finally, I want to emphasize that this is really a very interesting piece of research – all my comments are made with the intention to further improve this potentially highly insightful manuscript.

(Remarks on code availability)

Reviewer #2

(Remarks to the Author)
This paper presents an exciting research design and methodology for examining public support for agrivoltaics. However, I believe the framing of the paper currently limits its contribution. I find the NIMBY framing outdated and problematic (see, for example, Wolsink, M. (2006). Invalid theory impedes our understanding: a critique on the persistence of the language of NIMBY. Transactions of the Institute of British Geographers, 31(1), 85-91). What you call "NIMBY" (which is often framed as an illegitimate opposition) could be called "local opposition" or "local probjects" without loss of any meaning in the paper but with a more accurate and contemporary presentation. Further, even your data seem to suggest that NIMBY isn't empirically validated, so it's unlcear to me why the paper is framed around it and the term is used so frequently when actually what yor data seem to show is that the NIMBY concept isn't useful in the sense of empirically explanatory. I also don't understand the use of Agri-PV as opposed to the more common agrivoltaics. I also think the presentation of the data could be improved so as to clearly describe the effect sizes and make more direct claims about what actually matters in shaping support. In my read, the specific factors related to design and ownership seem to matter much more than local/non-local framings or partisanship of participant. If that's the case, that could be made much clearer throughout. I'm also a bit confused by the "personal advantage" variable and why it is included. The paper is about public support/public preferences. The "personal advantage" variable seems like it could only be intelligible if you present something about the employment of the respondents (are they farmers?). Seems like that could perhaps be left out of the paper entirely to help build a more coherent, consistent, and clear argument. The variables including "local energy production" and "own production" (Fig 5) are also unclear to me (local to the farm, or a local utility? who's "own" production?) and the lack of clarity raises questions about response validity for me. In sum, I believe this paper is a methodologically novel and important contribution to understanding public support for solar energy development, but I believe the paper can be significantly improved by changing the framing (get rid of the NIMBY language entirely, as it is outdated and not empirically validated in your analysis) and increasing clarity and precision in the results section (clarity what is actually most important for influencing support, and how do you know, and what are you actually trying to predict - public support?).

(Remarks on code availability)

Reviewer #4

(Remarks to the Author)
Introduction
Para 2 – it is not clear which country’s far-right voters are being discussed here? Switzerland or in general?
Why is rooftop solar, alone, not enough to achieve climate change mitigation and renewable energy targets? The authors do not provide any prior studies/references for the claim.
Methods
Supplementary information does not provide the breakup of respondents from each canton? How was it ensured that each canton was adequately represented in the survey?
Why were the young respondents less representative in the survey (between the ages 18-33) as compared to older individuals? Would that result in any bias of the findings of the survey?
What is the significance of including agricultural land not more than 1000 m from the building zone?
Why were grazing areas and standard fruit crop areas not considered?
It is unclear how the differentiation between “small” and “large” potential is done?
Any data regarding the profession of the respondents analysed?
What was the proportion of farmers in the survey?
Results
What does term “CH” represent in Figure 3b?
What was the criteria to qualify greenhouse integrated AV and scenario of 0-5% crop yield reduction could yield bipartisan support? How is it concluded?
Indicating left leaning as blue and right leaning as yellow should be done at the first mention in the results section.
Since the study is broadly comparing agri-PV with standard open space PV support, how did the authors both through the survey? Is there an analysis done showing the increase in support for agriv pv as compared to open space/traditional solar?
A chart doing side-by-side comparison of open space pv and agri-pv support for both right and left leaning individuals would

help establish the claim.
Discussion
What is the difference between the first and the second point you have highlighted from the findings of the study? Ostensibly, both elaborate the same point of left-leaning individuals updating their preferences after becoming knowledgeable of the location of Agri-PV.
Figure 6, tables 18 and 19 indicates Agri-PV systems – not the conventional PV systems. The difference in public support apparently lies within the Agri PV systems in itself.
Future research direction points two and three appear similar.

(Remarks on code availability)

Reviewer #5

(Remarks to the Author)
I co-reviewed this manuscript with one of the reviewers who provided the listed reports. This is part of the Nature Communications initiative to facilitate training in peer review and to provide appropriate recognition for Early Career Researchers who co-review manuscripts.

(Remarks on code availability)

Version 1:

Reviewer comments:

Reviewer #1

(Remarks to the Author)
Thanks for the careful revisions, which have further improved the manuscript. As far as I go, this is almost ready to be published. Four comments:

1.) The article portrays a relatively hopeful message, which is fine and backed by the empirics. However, there is one caveat: Large agrivoltaic installations do not seem to attract much support. However, exactly large projects might be most effective in producing renewable energy. If that's the case, I expect the manuscript do stress this problem more: support is rather there for small projects, not for big ones - even though from a climate action perspective, we might prefer large projects.

2.) "Arguably, however, not all types of solar energy technologies are symbolically charged to the same extent." -> It makes more sense to replace "symbolically charged" with "politically contested" here.

3.) The authors might double-check the appendix carefully. A major problem, at least in the PDF I received, is that many tables are barely readable because they are too big for the format of the pages.

4.) The authors discuss the limitations of the Swiss case in the conclusion. Also, the authors find little evidence for the effect of financial incentives. The might add a sentence that this might also be particularly in case in a high-income country such as Switzerland.

Again, thanks for this interesting piece of research!

(Remarks on code availability)

Reviewer #2

(Remarks to the Author)
I commend the authors on their careful and substantive revisions to the manuscript, which have greatly improved its clarity and contribution.

(Remarks on code availability)

Open Access This Peer Review File is licensed under a Creative Commons Attribution 4.0 International License, which permits use, sharing, adaptation, distribution and reproduction in any medium or format, as long as you give appropriate credit to the original author(s) and the source, provide a link to the Creative Commons license, and indicate if changes were made.
In cases where reviewers are anonymous, credit should be given to 'Anonymous Referee' and the source.
The images or other third party material in this Peer Review File are included in the article's Creative Commons license, unless indicated otherwise in a credit line to the material. If material is not included in the article's Creative Commons license and your intended use is not permitted by statutory regulation or exceeds the permitted use, you will need to obtain permission directly from the copyright holder.
To view a copy of this license, visit https://creativecommons.org/licenses/by/4.0/

Response Letter

Dear Reviewers,

We would like to thank you for your thoughtful and constructive feedback on our manuscript titled "Agrivoltaics can reduce political polarization and local opposition to solar energy on land." Your comments have significantly helped us improve the manuscript, and we have carefully considered each point. Below, we provide a detailed response to each of your comments, with specific revisions made in the manuscript. We have also copied examples of relevant track changes from the revised manuscript to demonstrate how we have addressed your comments.

Reviewer 1 (R1)

R1a: This is a very insightful paper based on an original and well-conducted survey experiment addressing a substantively interesting question: the acceptance of solar plants on land. This is a valuable contribution to the vast literature on the acceptance of renewable energy and therefore also interesting to policymakers. I recommend proceeding with the manuscript, but I have the following suggestions for revision.

Authors: Many thanks for the supportive and very constructive feedback!

R1b: Importantly, in terms of structure, the manuscript needs significant improvement: First, it should follow a typical research article structure: after the introduction should come the research design and method, followed by the results. At the moment, we learn about the method and survey details far too late – most of it appears only at the very end. Moreover, the results section currently includes methodological information, which is rather unusual. Second, the introduction needs to be substantially rewritten to prepare the readers about what's coming, without already including presentation of data.

Authors: We very much appreciate this feedback and have restructured the article—especially the Introduction (see track changes in the revised manuscript) – accordingly. In the Introduction, we removed the presentation of data, for example by moving the original Figure 1 to the Results section (now labeled Figure 2 in the revised manuscript). We also better prepare the reader for what follows in the subsequent sections. For example, see the following changes in the revised manuscript (lines 159ff.):

> *"In sum, our study underscores the importance of technology- and project-specific factors – especially the type and size of an agrivoltaics project, its ownership structure, and its impact on food production – in reducing local and right-leaning opposition to land-based solar energy. Furthermore, it highlights that realistic geolocated information about the potential of expanding agrivoltaics close to residents' homes is a less important factor in shaping public support than technology- and project-specific factors. Our study thus demonstrates the political potential of well-integrated agrivoltaics systems that minimize agricultural yield losses in overcoming local and right-leaning opposition against solar energy on land. In the following, we outline the key results of our study and discuss their implications for future research and policymaking. Please refer to the Methods section (below the Discussion) and the Supplementary Information (SI) for further details."*

In addition, following [Nature Communications' article format guidelines] (excerpted below), we retained the detailed Methods section at the end of the article:

> *"The main text of an Article should begin with a section headed Introduction of referenced text that expands on the background of the work (some overlap with the abstract is acceptable), followed by sections headed Results, Discussion (if appropriate) and Methods (if appropriate). The Results and Methods sections should be divided by topical subheadings; the Discussion should be succinct and may not contain subheadings. Methods are typically less than 3000 words. Figure legends are limited to 350 words each. As a guide, references should not exceed 70. Footnotes are not used."*

R1c: The notion of "bipartisanship" in the paper does not make sense given that the study deals with a multiparty context. "Bipartisanship" may be meaningful in the U.S., but certainly not in the context of the case study at hand. Similarly, and more substantively, I wonder whether the analysis should not distinguish simply between "left" and "right," but rather between clearly left-wing voters, centrist voters, and clearly right-wing voters. This would make the analysis more nuanced and avoid lumping together radical leftists (rightists) with individuals just slightly left (right) of center. Substantively, it would make more sense to structure the analysis around these categories. This would help to show which type of right-wing voters (more moderate ones?) can be reached by a different design of agrivoltaics.

Authors: Thank you very much. We fully agree with your suggestion and have implemented it accordingly in the revised manuscript. Throughout the paper and in all figures of the revised manuscript, we now distinguish clearly between left-leaning, centrist, and right-leaning respondents. This distinction allows us to provide a more nuanced analysis of how different types of voters respond to agrivoltaics design.

R1d: Food security and land use conflict: There should be more detail on the tension between land for solar installations and land for food production, in Switzerland and more generally.

Authors: In the revised manuscript, we elaborate on the tension between land for solar installations and land for food production, particularly in Switzerland, where agricultural land is limited and traditional landscapes and agriculture carry strong cultural value. This land-use trade-off is a salient issue in both political and public discourse, and we now highlight its relevance for agrivoltaics as a potential way to mitigate land-use conflicts. Please see, for instance, the following revisions in the revised manuscript:

> *Lines 53ff: However, while rooftop solar PV receives more support – even among right-leaning and local constituencies – its costs and implementation hurdles (e.g., grid connection, small project sizes) are higher than for solar energy projects on land [24,25]. Therefore, globally, rooftop solar PV is likely to cover only a share of the total demand for renewable energy needed to meet climate change mitigation and renewable energy targets, implying the need to expand land-based solar projects to meet these goals [25]. Yet, solar energy projects on land require space and can create important cultural, environmental, and economic land-use trade-offs, for example in the context of food and energy security [24,26,27]. Especially when agricultural land is limited, such as in countries like Switzerland, these land-use trade-offs become particularly salient. Moreover, solar energy projects on land can create other land-use*

trade-offs, such as spatial friction in biodiversity hotspots, human settlements, or recreational landscapes [24,26,27].

This constitutes an important political dilemma. On the one hand, to achieve climate change mitigation and renewable energy targets and become more energy independent, policymakers cannot rely only on rooftop solar PV and need to expand solar energy projects on land. On the other hand, these land-based solar energy projects face important political risks – especially in regions where renewable energy is polarized and where land competition is high and trade-offs are particularly salient.

Lines 126ff: "Switzerland offers a particularly relevant and informative research case due to its direct-democratic political system, its geographically inherent land-use trade-offs, and the polarized discussion about agriculture and the expansion of renewable energy on land [18,51,53]. Given Switzerland's scarce land availability, its strong cultural value of nature, traditional landscapes, and agriculture, and its increasing energy demand, land-use trade-offs are a salient topic – especially concerning energy and food production.

R1e: Ownership questions: The same applies here. The authors include multiple ownership structures in their research design, but the manuscript does not clearly explain how these were analyzed or which specific ownership types are most consequential.

Authors: We have provided a more detailed explanation of how different ownership structures were included and analyzed in our study. We also discuss the importance of local ownership in garnering public support.

For instance, we added the following paragraph to the revised manuscript:

Lines 93ff: "In addition, research on public support for other renewable energy technologies highlights the importance of local ownership, increasing local energy production, and higher incomes for local communities [2, 18]. Such local co-benefits of renewable energy projects – for instance, ownership by local municipalities or local businesses that allows economic revenues to remain in the region rather than being channeled to outside investors and energy companies – are key for local support [2, 18]."

R1f: Urban/rural divide: This is an issue of obvious importance but hardly explicitly discussed in the paper. I would expect much more on this, also with respect to the empirical analysis – as well as theory. An interesting recent reference is: 'Far-right against green: The re-emergence of geographically defined voting patterns and the new environment cleavage in Western Europe' in the European Political Science Review (2025).

Authors: Thank you for this suggestion. We agree that the urban–rural divide is central to understanding support for land-based solar projects. In the revised manuscript, we substantially expand the discussion of geographically structured cleavages in climate and energy politics, and we incorporate the suggested reference on the re-emergence of geographically defined voting patterns and a new environmental cleavage. We also strengthen the empirical analysis of the urban-rural divide. In addition to the ideological subgroup analysis, we now report and discuss subgroup effects for urban, suburban, and rural residents more explicitly in the

Results section (with additional analyses and robustness checks provided in the Supplementary Information):

Lines 99ff: "Local support is particularly important given the re-emergence of geographically defined – often rural – "policy losers," who might be more likely to bear, or perceive to bear, the concentrated costs of renewable energies compared to urban residents [6,7,44,45]. Nevertheless, rigorous quantitative and experimental research on political polarization and local opposition to different types of solar energy technologies on land – particularly agrivoltaics – remains scarce [36]. Especially, we lack knowledge on how realistic information about the local building potential of agrivoltaics projects and their design shape partisan support and local opposition to solar energy projects on land [1,14–19,21,46–49]."

Lines 264ff: "These findings suggest that key design elements – specifically, system type and visual integration, implications for agricultural output, and ownership structure – are central to securing majority support across the political spectrum. We find similar results when studying these design effects for urban, suburban, and rural residents (see SI Figure 10 and SI Table 25). Generally, support among urban and suburban residents is higher for agrivoltaics than among rural residents. Yet, local opposition from rural residents is unlikely if agrivoltaics projects are well integrated, create minimal crop yield reductions, and have local ownership."

Lines 272ff: "Interestingly, across ideological groups and urban–rural residents, the effect of agrivoltaics projects on local food production emerges as a more critical factor than their impact on local energy output. As discussed above and shown in Figure 4, minimal decreases in food production (0–5%) attract majority support across ideological and urban–rural respondents, whereas large reductions (41–80%) lead to widespread rejection. By contrast, agrivoltaics projects' substantial increases in electricity generation in the local municipality positively affect support, but less strongly than agrivoltaics projects that minimize the reduction of domestic food production. This suggests that concerns related to domestic food production and security weigh more heavily than considerations related to domestic energy production in shaping cross-ideological and urban-rural attitudes toward agrivoltaics deployment. In addition, these results underscore opposition to open-space PV on agricultural land and buttress the political importance of the dual land-use function of agrivoltaics projects."

Lines 352ff: "Comparing the effect sizes of the geolocated information treatment with those of project-technology design across ideological groups and urban–rural populations, it is evident that design effects are larger. Our results thus show that while partisan polarization, urban–rural divides, and negative effects from information about local expansion potential are present, these effects can be mitigated through smart project and technology design."

R1g: Finally, regarding case selection, direct democracy and the referendum is mentioned but never clearly justified as relevant for case selection. Why should we care? Presumably, this is due to issue salience – but this should be made explicit and discussed more clearly. The question of issue salience of solar energy in comparison to other renewables, particularly wind power, merits more discussion more generally. It might be less symbolically charged than wind power and therefore more prone to attitude shift. An interesting recent reference is: 'Climate action versus environmental protection? How far-right and environmentalist messengers shape public attitudes towards renewable energy infrastructure in forests' in *Environmental Politics* (2025).

Authors: In the revised manuscript, we make a clearer case for the relevance of Switzerland's direct-democratic system in the context of agrivoltaics. Direct democracy provides an important setting for testing public opinion on energy policies

because issue salience is heightened during referendums and voters have a direct say in decision-making. Switzerland is therefore an externally valid case for studying public support for solar PV on land. In addition, we now emphasize that solar energy may be less symbolically charged than other low-carbon technologies and therefore more prone to attitude shifts.

> *Lines 435ff (Methods): "The data collection occurred between May 3 and June 8, 2024, in the run-up to a publicly salient national referendum on the Electricity Law, which aimed to accelerate renewable energy deployment, including open-space PV. The unique timing and context of the direct-democratic referendum provide strong external validity given the increased issue salience and voters' direct say in political decision-making.*
>
> *In addition, Switzerland is a particularly relevant study case due to its direct-democratic political system, geographic conditions, and cultural values. Citizens routinely vote on land-use and energy issues, such as the Electricity Law, amplifying the role of public support in actual political decision-making. The country's mountainous geography and scarcity of arable land create tensions between agriculture and renewable energy infrastructure. These tensions are often amplified in polarized political discussions between right-leaning and left-leaning parties and by influential lobby groups, such as the powerful Swiss farmer association. Moreover, Swiss citizens place high value on landscape preservation as well as food and energy self-sufficiency [50]. Additionally, most research on local opposition to renewable energy has focused on the United States and United Kingdom [14], while Switzerland's increasingly polarized political climate around climate policy and renewable energy [51,53] makes it an ideal context for studying ideological and urban-rural variation in renewable energy, especially agrivoltaics, support."*

R1h: The writing would benefit from careful proofreading. For instance, already the second sentence reads oddly, with the word "land" used three times in a single sentence. P. 3: „To address 82 this research gap, we experimentally analyze how personalized geolocated information 83 about the realistic Agri-PV building potential and different technology and project 84 design characteristics of Agri-PV jointly affect public support across ideological groups.": Why „jointly" here?

Authors: Thank you very much. We carefully proofread the full manuscript to remove redundancies and to improve wording, grammar, and overall readability (see track changes in revised manuscript). We also revised the specific sentences marked by the reviewer and deleted the term "jointly" from the respective sentence.

R1i: Finally, I want to emphasize that this is really a very interesting piece of research – all my comments are made with the intention to further improve this potentially highly insightful manuscript.

Authors: We very much appreciate your positive feedback!

Reviewer 2 (R2)

R2a: This paper presents an exciting research design and methodology for examining public support for agrivoltaics. However, I believe the framing of the paper currently limits its contribution. I find the NIMBY framing outdated and problematic (see, for example, Wolsink, M. (2006). Invalid theory impedes our understanding: a critique on the persistence of the language of NIMBY. Transactions of the Institute of British Geographers, 31(1), 85-91). What you call "NIMBY" (which is often framed as an illegitimate opposition) could be called "local opposition" or "local probjects" without loss of any meaning in the paper but with a more accurate and contemporary presentation. Further, even your data seem to suggest that NIMBY isn't empirically validated, so it's unlcear to me why the paper is framed around it and the term is used so frequently when actually what your data seem to show is that the NIMBY concept isn't useful in the sense of empirically explanatory.

Authors: Thank you very much for the constructive and helpful comments. We agree and have removed the term “NIMBY” entirely, replacing it with the more accurate and contemporary term “local opposition.” We also cited the suggested reference, Wolsink, M. (2006). Please see all track-changes throughout the revised manuscript, such as:

> *Revised abstract, Lines 9ff: “As solar energy deployment on land intensifies, political polarization and local opposition increasingly challenge its social acceptance. We propose that agrivoltaics – a dual land-use technology that combines solar power generation with agricultural production – can help bridge ideological divides and reduce local opposition by minimizing land-use conflicts and offering additional benefits to rural communities. To test this hypothesis, we conducted a pre-registered, field-embedded experiment with a representative sample of 2,132 Swiss residents just prior to a national renewable energy referendum in June 2024. The study combined a personalized geolocated information treatment – highlighting the realistic local potential for agrivoltaics deployment – with a conjoint experiment varying key technology and project design attributes. We find that agrivoltaics garners broad public support across ideological groups and reduces local opposition, especially when projects are well integrated into agricultural landscapes, do not significantly reduce agricultural yields, and are locally owned. These findings underscore the importance of technology and project design in addressing local and right-leaning opposition to land-based solar energy.”*

> *Lines 93ff: “In addition, research on public support for other renewable energy technologies highlights the importance of local ownership, increasing local energy production, and higher incomes for local communities [2, 18]. Such local co-benefits of renewable energy projects – for instance, ownership by local municipalities or local businesses that allows economic revenues to remain in the region rather than being channeled to outside investors and energy companies – are key for local support [2, 18]. Local support is particularly important given the re-emergence of geographically defined – often rural – “policy losers,” who might be more likely to bear, or perceive to bear, the concentrated costs of renewable energies compared to urban residents [6,7,44,45]. Nevertheless, rigorous quantitative and experimental research on political polarization and local opposition to different types of solar energy technologies on land – particularly agrivoltaics – remains scarce [36]. Especially, we lack knowledge on how realistic information about the local building potential of agrivoltaics projects and their design shape partisan support and local opposition to solar energy projects on land [1,14–19,21,46–49].”*

R2b: I also don't understand the use of Agri-PV as opposed to the more common agrivoltaics.

Authors: Thanks. We have replaced the term "Agri-PV" with "agrivoltaics" throughout the entire revised manuscript to align with common terminology in the field.

R2c: I also think the presentation of the data could be improved so as to clearly describe the effect sizes and make more direct claims about what actually matters in shaping support. In my read, the specific factors related to design and ownership seem to matter much more than local/non-local framings or partisanship of participant. If that's the case, that could be made much clearer throughout.

Authors: We have restructured the revised manuscript, especially the Results and Discussion sections, to clearly highlight the most important factors influencing public support for agrivoltaics. We have also provided more precise effect sizes throughout the revised manuscript. For instance, see the following exemplary revisions:

> *Lines 159ff: "In sum, our study underscores the importance of technology- and project-specific factors – especially the type and size of an agrivoltaics project, its ownership structure, and its impact on food production – in reducing local and right-leaning opposition to land-based solar energy. Furthermore, it highlights that realistic geolocated information about the potential of expanding agrivoltaics close to residents' homes is a less important factor in shaping public support than technology- and project-specific factors. Our study thus demonstrates the political potential of well-integrated agrivoltaics systems that minimize agricultural yield losses in overcoming local and right-leaning opposition against solar energy on land."*
>
> *Lines 186ff: "Figures 3a to 3c reveal that respondents across the ideological spectrum – i.e., left-leaning (blue), centric (grey), and right-leaning (yellow) respondents – express significantly lower support for the expansion of medium- to large-scale agrivoltaics installations (up to ten football pitches, ≈10 ha) compared to small-scale (≈1 ha) projects. Large-scale agrivoltaics projects receive substantially lower support among all groups, in particular among right-leaning residents. For right-leaning residents, the estimated mean support rating for large-scale projects on a 7-point scale is 3.5 (see Fig. 3c), while for small-scale projects the estimated mean rating is 4.5 (see Fig. 3a). Overall, a clear ideological divide persists: left-leaning and centric respondents consistently express greater support for agrivoltaics expansion across all project sizes, whereas support among right-leaning individuals remains comparatively lower.*
>
> *Notably, the geolocated treatment providing respondent-tailored information on the realistic local potential of expanding agrivoltaics close to their homes has only a weak negative support effect for large agrivoltaics projects (but not for smaller projects).*
>
> *Figures 3d and 3e reinforce the observed pattern, showing that left-leaning and centric respondents express higher support for expanding agrivoltaics in Switzerland and in respondents' neighborhoods compared to right-leaning individuals. Again, the geolocated information treatment has only weak dampening support effects (i.e., −0.123 to −0.155 points on a 7-point Likert scale; see details in SI Section B, Table 10)."*
>
> *Lines 337ff: "Our findings show that – unlike open-space solar energy projects on land (see Figure 1) – agrivoltaics as a dual land-use technology can reduce political opposition and increase cross-ideological and urban–rural support for solar energy on land.*
>
> *A first key takeaway from our study is that technology and project design attributes of agrivoltaics – such as their integration with existing agricultural infrastructure, their size,*

ownership structure, and the impact on crop production – play a crucial role in shaping support across ideological groups and urban–rural populations. Left-leaning and urban residents consistently show stronger support for agrivoltaics, but majorities – even among right-leaning and rural populations – emerge when installations are landscape integrated, not large scale, locally owned, and do not result in substantial yield reductions. This highlights the potential of agrivoltaics – compared to open-space solar energy – as a politically viable renewable energy solution, especially in polarized political contexts.

A second key takeaway from our experiment is that the personalized geolocated information has modest support effects and only interacts with some agrivoltaics technology and project design attributes. Comparing the effect sizes of the geolocated information treatment with those of project-technology design across ideological groups and urban–rural populations, it is evident that design effects are larger. Our results thus show that while partisan polarization, urban–rural divides, and negative effects from information about local expansion potential are present, these effects can be mitigated through smart project and technology design."

R2d: I'm also a bit confused by the "personal advantage" variable and why it is included. The paper is about public support/public preferences. The "personal advantage" variable seems like it could only be intelligible if you present something about the employment of the respondents (are they farmers?). Seems like that could perhaps be left out of the paper entirely to help build a more coherent, consistent, and clear argument.

Authors: We agree with the reviewer and have removed the "advantage" variables and respective figures from the revised manuscript, as it did not contribute to our central argument on public support for agrivoltaics.

R2e: The variables including "local energy production" and "own production" (Fig 5) are also unclear to me (local to the farm, or a local utility? who's "own" production?) and the lack of clarity raises questions about response validity for me.

Authors: We have clarified the meanings of "local energy production" and "own production." Specifically, "local energy production" refers to the energy produced within the municipality in which the respective agrivoltaics project is located, and "own food production" refers to the project's local agricultural crop yield. In addition, we note that all attributes presented in the conjoint experiment were clearly defined and explained to respondents prior to the choice tasks, helping ensure high response validity. To reflect this more explicitly, we revised the manuscript – especially the Results, Discussion, and Methods sections (see also Method Table 1) – accordingly. For example, we made the following revisions:

Lines 272ff: "Interestingly, across ideological groups and urban–rural residents, the effect of agrivoltaics projects on local food production emerges as a more critical factor than their impact on local energy output. As discussed above and shown in Figure 4, minimal decreases in food production (0–5%) attract majority support across ideological and urban–rural respondents, whereas large reductions (41–80%) lead to widespread rejection. By contrast, agrivoltaics projects' substantial increases in electricity generation in the local municipality positively affect support, but less strongly than agrivoltaics projects that minimize the reduction of local food production. This suggests that concerns related to local food production and security weigh more heavily than considerations related to local energy production in shaping cross-ideological and urban-rural attitudes toward agrivoltaics deployment. In addition, these results underscore opposition to open-space PV on agricultural land and buttress the political importance of the dual land-use function of agrivoltaics projects."

Lines 362ff: "In addition, we find that citizens are particularly sensitive to the impact of land-based solar projects on local food production, whereas they are less concerned about impacts on local energy production. This underscores the importance for policymakers of promoting agrivoltaics projects that minimize crop yield losses to garner cross-ideological and local support."

Lines 507ff: "After the personalized geolocated information treatment, we fielded a conjoint experiment to assess the support effects of the information treatment for differently designed agrivoltaics projects across ideological and rural–urban populations. First, we introduced to respondents all agrivoltaics' technology and project design attributes used in the conjoint experiment (see Method Table 1). We selected these design attributes based on a literature review [e.g., 36–41,46] and expert knowledge of key renewable and solar energy project design attributes. Specifically, we included those agrivoltaics project and technology design attributes that we expected to affect support across ideological groups and local residents. Second, we then implemented four successive choice rounds in which respondents were shown two agrivoltaics project profiles side by side and asked to indicate both their preferred option and the degree to which they supported or opposed each option on a 7-point Likert scale (see exact wording in SI Section C). Each agrivoltaics project option was described using the seven design attributes, with attributes fully randomized within and across agrivoltaics project options. This allows the non-parametric identification of causal effects of the attributes. respondents then Conjoint experiments work by randomly combining different levels of attributes (in our case related to differently designed agrivoltaics projects) into full-profile options and then asking respondents to choose between two such options [55–57]. Because participants weigh all attributes simultaneously, the exercise mirrors the multidimensional trade-offs people make in real-world decisions more closely than single-attribute rating tasks [55–57]. This advantage has led to a rapid rise in its use to study public preferences across the social sciences in recent years [55–60], including in the context of renewable energy [2]. Evidence shows that conjoint designs deliver findings with strong external and ecological validity and are less prone to social desirability bias than many alternative survey techniques [55,56]."

R2f: In sum, I believe this paper is a methodologically novel and important contribution to understanding public support for solar energy development, but I believe the paper can be significantly improved by changing the framing (get rid of the NIMBY language entirely, as it is outdated and not empirically validated in your analysis) and increasing clarity and precision in the results section (clarity what is actually most important for influencing support, and how do you know, and what are you actually trying to predict - public support?).

Authors: We greatly appreciate the reviewer's valuable feedback and hope that our revisions adequately address the suggestions raised. In particular, we removed the NIMBY framing from the manuscript and sought to improve clarity and precision in the results and discussion sections, particularly highlighting the key factors shaping public support for agrivoltaics projects.

<u>Reviewer 3 (R3)</u>

R3a: Para 2 – it is not clear which country's far-right voters are being discussed here? Switzerland or in general?

Authors: We greatly appreciate the reviewer's constructive comments. Our paper is empirically focused on public support for land-based solar energy in Switzerland. At the beginning of the article, however, we situate this discussion in a broader comparative context and describe parallel trends in far-right and local opposition across countries, including Italy and the United States. To address the reviewer's comment, we revised the second paragraph to clarify both the case selection and the empirical focus on Switzerland. In addition, we moved all empirical results on the Swiss case – for example, the original Figure 1, now labeled Figure 2 in the revised manuscript – into the Results section. Please see for instance the following exemplary revisions:

> *Lines 26ff: "Many renewable energy technologies have seen remarkable cost reductions and diffusion, but the expansion of these technologies has become a highly contested and polarized political issue [1–11]. This is particularly the case for large renewable energy projects on land, such as open-space solar photovoltaic (PV) projects, and it puts the expansion of such land-based solar energy initiatives at risk [1,2]. Right-wing populist parties across many countries have increasingly politicized solar energy, with legal initiatives to limit solar projects, such as in Italy [12] or the United States [13]. However, even right-wing populist parties differ in how they position themselves toward the technology: some, like Germany's Alternative für Deutschland (AfD), oppose it outright, while others, including Italy's Lega and Fratelli d'Italia, express conditional support [1]. These divergent stances challenge the notion of a uniform ideological opposition to solar energy and underscore the importance of political strategy, technology- and project-specific factors in shaping public and party positions on renewable energy. At the same time, local opposition to renewable energy projects near individuals' homes challenges their expansion [14–17], and research indicates that the specific characteristics of these projects can be pivotal for generating support among local policymakers and residents [1,2,7,9–11,14,15,18–20].*
>
> *Compared to other renewable energy technologies, such as wind power plants, solar energy has only more recently become salient in public discourse and for right-wing parties [1,21–23]. Arguably, however, not all types of solar energy technologies are symbolically charged to the same extent. As a result, voter and party support for solar energy is likely to depend on information about local building potential and on specific project and technology design. In fact, support for solar energy in many countries is not homogeneous across different types of solar energy technologies [1,9,19]. For instance, right-leaning voters and parties across various countries tend to welcome rooftop solar projects [9] but oppose large-scale solar projects on land, which are perceived to threaten rural aesthetics, agricultural production, or local control [1,19]."*
>
> *Lines 126ff: "Switzerland offers a particularly relevant and informative research case due to its direct-democratic political system, its geographically inherent land-use trade-offs, and the polarized discussion about agriculture and the expansion of renewable energy on land [18,51,53]. Given Switzerland's scarce land availability, its strong cultural value of nature, traditional landscapes, and agriculture, and its increasing energy demand, land-use trade-offs are a salient topic – especially concerning energy and food production. The data collection occurred between 3 May and 8 June 2024, in the run-up to a publicly salient national referendum on 9 June 2024 on the Electricity Law, which aimed to accelerate renewable*

energy deployment, including open-space PV. This unique timing and context provide strong internal and external validity."

Lines 173ff: "Figure 2 shows that around 60 percent of right-leaning residents in Switzerland and more than 50 percent of residents with centric political views do not support open-space solar projects. In contrast, similar to rooftop solar PV, agrivoltaics projects generally enjoy broad political support among left-, centric, and right-leaning residents in Switzerland. For instance, more than 60 percent of right-leaning residents support agrivoltaics (see Figure 2).

Moreover, we find similar results for rural areas that are most affected by such projects. While open-space PV projects receive less than 40 percent support among rural residents, agrivoltaics projects receive almost 60 percent support in rural areas (see SI Figure 5)."

R3b: Why is rooftop solar, alone, not enough to achieve climate change mitigation and renewable energy targets? The authors do not provide any prior studies/references for the claim.

Authors: We fully agree. We revised the manuscript and added references to support the claim that rooftop solar alone is unlikely to meet renewable energy and climate change mitigation targets, given its higher costs and implementation hurdles. For example, we made the following changes in the revised manuscript:

Lines 53ff: "However, while rooftop solar PV receives more support – even among right-leaning and local constituencies – its costs and implementation hurdles (e.g., grid connection, small project sizes) are higher than for solar energy projects on land [24,25]. Therefore, globally, rooftop solar PV is likely to cover only a share of the total demand for renewable energy needed to meet climate change mitigation and renewable energy targets, implying the need to expand land-based solar projects to meet these goals [25]. Yet, solar energy projects on land require space and can create important cultural, environmental, and economic land-use trade-offs, for example in the context of food and energy security [24,26,27]. Especially when agricultural land is limited, such as in countries like Switzerland, these land-use trade-offs become particularly salient. Moreover, solar energy projects on land can create other land-use trade-offs, such as spatial friction in biodiversity hotspots, human settlements, or recreational landscapes [24,26,27].

This constitutes an important political dilemma. On the one hand, to achieve climate change mitigation and renewable energy targets and become more energy independent, policymakers cannot rely only on rooftop solar PV and need to expand solar energy projects on land. On the other hand, these land-based solar energy projects face important political risks – especially in regions where renewable energy is polarized and where land competition is high and trade-offs are particularly salient."

R3c: Supplementary information does not provide the breakup of respondents from each canton? How was it ensured that each canton was adequately represented in the survey?

Authors: Thank you. We worked with the Swiss Federal Statistical Office (BfS), which, through a restricted data-access agreement available only to Swiss Federal Research Institutes, provided a representative sample of the Swiss resident population in all German- and French-speaking cantons. Our final sample is representative of these cantons. We revised the Methods section accordingly to address this comment:

Lines 420ff: "The respondents in our survey are drawn from the population register of the Federal Statistical Office (BfS), comprising Swiss residents. The BfS population register mirrors, aside from random error and uneven response rates, the Swiss resident population. We used stratified random sampling with the strata language region (i.e., German- or French-speaking cantons of Switzerland), gender (i.e., male or female), and urbanization level (i.e., urban, semi-urban, rural). We invited a random sample of Swiss residents in the French- and German-speaking cantons of Switzerland above the age of 18 years via letter (plus one reminder) to participate in our online survey via the survey platform Qualtrics. After excluding respondents who completed the survey in less than 40 percent of the median response time (44 minutes), as well as unclear responses and non-completes, our final sample consisted of 2,132 Swiss residents. As shown in SI Section A, our random sample is a close approximation of the Swiss resident population in the French- and German-speaking cantons of Switzerland above the age of 18 years. Younger respondents (age 18-33) are slightly underrepresented, while older respondents (age 58-78) are slightly overrepresented in your survey."

R3d: Why were the young respondents less representative in the survey (between the ages 18-33) as compared to older individuals? Would that result in any bias of the findings of the survey?

Authors: Thanks for noting this point. Indeed, younger respondents (ages 18–33) are slightly underrepresented, while older respondents (ages 58–78) are slightly overrepresented in our survey. We now state this explicitly in the Methods section (see the revised paragraph in our previous response, lines 420ff). Respondents were invited by postal mail, which likely contributed to a higher response rate among older residents. This pattern is consistent with prior surveys conducted in collaboration with the Swiss Federal Statistical Office (BfS).

If anything, the underrepresentation of younger respondents would likely lead us to underestimate overall population support for agrivoltaics, given that younger residents in Switzerland generally – and also in our sample (see the SI regression tables) – express slightly higher support for solar energy than older residents, albeit with a small marginal effect. Importantly, this slight age underrepresentation does not threaten the internal validity of our experimental estimates: we (a) control for age in the regression models, and (b) our balance checks indicate no statistically significant differences in age between treatment and control groups.

R3e: What is the significance of including agricultural land not more than 1000 m from the building zone? Why were grazing areas and standard fruit crop areas not considered?

Authors: Thank you. We have clarified the significance of this criterion and how it aligns with the current Swiss regulatory framework and grid. For instance, please see the following revisions in our revised Method section:

Lines 455ff: "For our pre-registered experiment (see pre-registration details [54]), we developed a GIS-based dataset that maps the fine-grained, geolocated building and solar power generation potential of agrivoltaics installations in Switzerland [52].

The dataset is based on [52], which shows the geolocated technical potential for agrivoltaics in Switzerland. It includes agricultural land located no more than 1,000 m from a building zone and potentially suitable for agrivoltaics. This is consistent with the current regulatory framework in Switzerland and accounts for grid connection. Areas with low horizontal irradiation (less than 1,000 kWh/m2) were excluded, as were summer grazing areas and areas that are obviously unsuitable for agrivoltaics. Furthermore, areas in national protected zones such as BLN, national parks, or moorlands were classified as unusable. The remaining technical potential is available as a polygon dataset and can thus be narrowed down to any geographical region."

R3f: It is unclear how the differentiation between "small" and "large" potential is done?

Authors: As outlined in the manuscript, we clarify the methodology for distinguishing small and large potential areas for agrivoltaics. Please see the revised Method section:

Lines 467ff: "This polygon dataset was combined with participants' home address data from the Swiss Federal Statistical Office, allowing us to construct an individualized information treatment condition. The randomly selected control group of participants received generic information about agrivoltaics, while the treatment group was additionally shown a personalized map highlighting the realistic potential for building agrivoltaics plants around their home within a defined radius (0–500 m, 500–1,500 m, and 1,500–4,500 m) [52]. As shown in the example treatment information below, the realistic potential (GWh/a) within each pre-defined radius was either displayed as large (upper 1/3 of the distribution of solar power generation potential of all respondents in the respective radius) or small (lower 2/3 of the distribution of solar power generation potential of all respondents in the respective radius). Our balance checks (see SI Section B) indicate that the treatment randomization worked as expected and the control and treatment samples are well balanced."

R3g: Any data regarding the profession of the respondents analysed? What was the proportion of farmers in the survey?

Authors: Unfortunately, we did not collect data on the profession of the respondents. Future research may consider incorporating this variable to enhance understanding of profession-related preferences towards solar energy on land, such as agrivoltaics.

R3h: What does term "CH" represent in Figure 3b?

Authors: "CH" denotes Switzerland. We have removed Figure 3b in the revised manuscript; please see our response to Reviewer 2, comment (R2d), for details.

R3i: What was the criteria to qualify greenhouse integrated AV and scenario of 0-5% crop yield reduction could yield bipartisan support? How is it concluded?

Authors: As outlined in the Methods section, we base this finding on nonparametrically estimated Average Marginal Interaction Effects (AMIEs) using an ANOVA regression with weighted zero-sum constraints, following Egami and Imai [61]. This approach estimates support for complex, multi-attribute profiles by accounting for higher-order interactions among attribute levels, independent of baseline category choices. It also allows us to identify the most and least preferred agrivoltaics project configurations across the full factorial design space (see SI Section B, Figure 11 and Tables 31 and 32).

R3j: Indicating left leaning as blue and right leaning as yellow should be done at the first mention in the results section.

Authors: We have added a note at the beginning of the Results section clarifying that blue denotes left-leaning respondents and yellow denotes right-leaning respondents. See, for instance, the following revision:

> *Lines 186ff: "Figures 3a to 3c reveal that respondents across the ideological spectrum – i.e., left-leaning (blue), centric (grey), and right-leaning (yellow) respondents – express significantly lower support for the expansion of medium- to large-scale agrivoltaics installations (up to ten football pitches, ≈10 ha) compared to small-scale (≈1 ha) projects."*

R3k: Since the study is broadly comparing agri-PV with standard open space PV support, how did the authors both through the survey? Is there an analysis done showing the increase in support for agriv pv as compared to open space/traditional solar? A chart doing side-by-side comparison of open space pv and agri-pv support for both right and left leaning individuals would help establish the claim.

Authors: Thank you for this important suggestion. We have updated Figure 2 (previously Figure 1) in the revised manuscript to present a side-by-side comparison of support for open-space PV and agrivoltaics among both right- and left-leaning individuals. Please see the following revisions:

> *Lines 173ff: "Figure 2 shows that around 60 percent of right-leaning residents in Switzerland and more than 50 percent of residents with centric political views do not support open-space solar projects. In contrast, similar to rooftop solar PV, agrivoltaics projects generally enjoy broad political support among left-, centric, and right-leaning residents in Switzerland. For instance, more than 60 percent of right-leaning residents support agrivoltaics (see Figure 2).*
>
> *Moreover, we find similar results for rural areas that are most affected by such projects. While open-space PV projects receive less than 40 percent support among rural residents, agrivoltaics projects receive almost 60 percent support in rural areas (see SI Figure 5)."*

R3l: What is the difference between the first and the second point you have highlighted from the findings of the study? Ostensibly, both elaborate the same point of left-leaning individuals updating their preferences after becoming knowledgeable of the location of Agri-PV.

Authors: Thanks. We agree and deleted these parts from the revised manuscript.

R3m: Figure 6, tables 18 and 19 indicates Agri-PV systems – not the conventional PV systems. The difference in public support apparently lies within the Agri PV systems in itself.

Authors: In addition to the difference in public support between open-space PV and agrivoltaics (see our response to Comment R3k), we observe substantial variation in support across different agrivoltaics system types. This is a central finding of the study, and we have made it much more explicit throughout the revised manuscript. Thank you very much. Please see for instance:

> *Revised abstract, Lines 9ff: "As solar energy deployment on land intensifies, political polarization and local opposition increasingly challenge its social acceptance. We propose that agrivoltaics – a dual land-use technology that combines solar power generation with agricultural production – can help bridge ideological divides and reduce local opposition by minimizing land-use conflicts and offering additional benefits to rural communities. To test this hypothesis, we conducted a pre-registered, field-embedded experiment with a representative sample of 2,132 Swiss residents just prior to a national renewable energy referendum in June 2024. The study combined a personalized geolocated information treatment – highlighting the realistic local potential for agrivoltaics deployment – with a conjoint experiment varying key technology and project design attributes. We find that agrivoltaics garners broad public support across ideological groups and reduces local opposition, especially when projects are well integrated into agricultural landscapes, do not significantly reduce agricultural yields, and are locally owned. These findings underscore the importance of technology and project design in addressing local and right-leaning opposition to land-based solar energy."*

> *Lines 159ff: "In sum, our study underscores the importance of technology- and project-specific factors – especially the type and size of an agrivoltaics project, its ownership structure, and its impact on food production – in reducing local and right-leaning opposition to land-based solar energy. Furthermore, it highlights that realistic geolocated information about the potential of expanding agrivoltaics close to residents' homes is a less important factor in shaping public support than technology- and project-specific factors. Our study thus demonstrates the political potential of well-integrated agrivoltaics systems that minimize agricultural yield losses in overcoming local and right-leaning opposition against solar energy on land."*

R3n: Future research direction points two and three appear similar.

Authors: We agree and have revised the future research section accordingly.

We hope these revisions meet your expectations and enhance the clarity and quality of the manuscript. Thank you once again for your constructive feedback.

<u>Response Letter “Agrivoltaics can reduce political polarization and local opposition to solar energy on land”</u>

Reviewer #1 (Remarks to the Author):

R1: Thanks for the careful revisions, which have further improved the manuscript. As far as I go, this is almost ready to be published.

Authors: Thank you very much for the supportive and very constructive feedback!

R1: 1.) The article portrays a relatively hopeful message, which is fine and backed by the empirics. However, there is one caveat: Large agrivoltaic installations do not seem to attract much support. However, exactly large projects might be most effective in producing renewable energy. If that's the case, I expect the manuscript do stress this problem more: support is rather there for small projects, not for big ones - even though from a climate action perspective, we might prefer large projects.

Authors: Thank you very much for this comment. We agree that this result requires a more detailed and nuanced discussion. To address this important point, we revised the discussion section accordingly, for instance the following part.

Lines 368ff in revised manuscript:

> *“Finally, policymakers may face a trade-off between minimizing political opposition and containing economic costs when expanding land-based solar energy. As this study demonstrates, political risks can be mitigated by promoting agrivoltaics, particularly when systems are smaller scale and carefully integrated into the landscape – for instance, by replacing existing structures such as greenhouses or polytunnels –, and when they do not significantly reduce agricultural yields. However, levelized electricity costs for agrivoltaics vary depending on design, ranging from approximately 10% higher than conventional open-space PV for low-mounted horizontal systems, to as much as 50% higher for elevated structures positioned more than 4 meters above the ground [52]. Especially, large low-mounted horizontal systems can benefit from economies of scale and less customization costs than smaller, more integrated agrivoltaics systems. Landscape-integrated agrivoltaics systems positioned up to 2.5 meters above ground (see Figure 1a) typically incur a 30–40% cost premium [52]. This constitutes a potential trade-off for policymakers between efficiency and feasibility: Large-scale open-space PV and low-mounted horizontal agrivoltaics systems are more cost-efficient to reach renewable energy targets but are publicly less accepted – especially among right-wing and rural residents – than smaller, more integrated agrivoltaics systems. Yet these more-integrated designs can offset part of their higher costs by reducing yield losses from extreme weather events (e.g., hail) and substituting for other farm infrastructure (e.g., polytunnels). Moreover, while well-integrated systems do raise electricity generation costs, they typically lead to lower yield losses and deliver clear political co-benefits. It is also likely that, due to learning effects, costs – especially for well-integrated systems – may fall further over time. Notably, we find that public support for well-integrated designs with minimal impact on crop yield is up to 40–50 percentage points higher than for large-scale open-space PV, vertical, or horizontal agrivoltaics installations that lead to higher yield losses (see SI Section B). These results suggest that, depending on sociopolitical conditions, the political advantages of well-integrated agrivoltaics designs may outweigh their higher economic costs compared to open-space PV, particularly if such projects help forge new coalitions, including skeptical right-leaning voters and farmers.”*

R1: 2.) "Arguably, however, not all types of solar energy technologies are symbolically charged to the same extent." -> It makes more sense to replace "symbolically charged" with "politically contested" here.

Authors: We fully agree and adapted the wording in the revised manuscript accordingly.

R1: 3.) The authors might double-check the appendix carefully. A major problem, at least in the PDF I received, is that many tables are barely readable because they are too big for the format of the pages.

Authors: Many thanks. We have double-checked the appendix and tried to improve the readability of the tables.

R1: 4.) The authors discuss the limitations of the Swiss case in the conclusion. Also, the authors find little evidence for the effect of financial incentives. The might add a sentence that this might also be particularly in case in a high-income country such as Switzerland.

Authors: To address this valuable feedback, we revised the discussion section. Please see changes in Lines 406ff in the revised manuscript:

> *First, the study is based on the Swiss context, which is characterized by direct-democratic institutions, strong land competition, and polarized political dynamics around climate policies and renewable energy [18, 51,53]. Moreover, Switzerland is a high-income country with – compared to other countries – large subsidies to farmers [63]. This context might explain some of the results, for instance, the limited public support for additional financial support to farmers. Therefore, the extent to which our findings can be generalized to other countries with different institutional structures, land-use pressures, economic and political environments remains an open question.*

R1: Again, thanks for this interesting piece of research!

Authors: Many thanks.

<u>Reviewer #2 (Remarks to the Author):</u>

R2: I commend the authors on their careful and substantive revisions to the manuscript, which have greatly improved its clarity and contribution.

Authors: Many thanks for the careful review, very constructive feedback and positive evaluation.
